# Genome-wide identification and expression analysis of the *VQ* gene family in soybean (*Glycine max*)

**DOI:** 10.7717/peerj.7509

**Published:** 2019-08-21

**Authors:** Yongbin Wang, Zhenfeng Jiang, Zhenxiang Li, Yuanling Zhao, Weiwei Tan, Zhaojun Liu, Shaobin Cui, Xiaoguang Yu, Jun Ma, Guangjin Wang, Wenbin Li

**Affiliations:** 1 Key Laboratory of Soybean Biology in Chinese Ministry of Education, Key Laboratory of Soybean Biology and Breeding/Genetics of Chinese Agriculture Ministry, Northeast Agricultural University, Harbin, Heilongjiang, China; 2 Biotechnology Research Institute, Heilongjiang Academy of Agricultural Sciences, Harbin, Heilongjiang, China; 3 Harbin Normal University, Harbin, Heilongjiang, China; 4 Heilongjiang Academy of Agricultural Sciences, Harbin, Heilongjiang, China; 5 Soybean Research Institute, Heilongjiang Academy of Agricultural Sciences, Harbin, Heilongjiang, China

**Keywords:** VQ gene family, Glycine max, Gene expression, Phylogenetic analysis, Bioinformatics

## Abstract

**Background:**

VQ proteins, the plant-specific transcription factors, are involved in plant development and multiple stresses; however, only few articles systematic reported the *VQ* genes in soybean.

**Methods:**

In total, we identified 75 *GmVQ* genes, which were classified into 7 groups (I-VII). Conserved domain analysis indicated that *VQ* gene family members all contain the *VQ* domains. *VQ* genes from the same evolutionary branches of soybean shared similar motifs and structures. Promoter analysis revealed that *cis*-elements related to stress responses, phytohormone responses and controlling physical as well as reproductive growth. Based on the RNA-seq and qRT-PCR analysis, *GmVQ* genes were showed expressing in nine tissues, suggesting their putative function in many aspects of plant growth and development as well as response to stress in *Glycine max*.

**Results:**

This study aims to understand the roles of *VQ* genes in various development processes and their expression patterns in responses to stimuli. Our results provide basic information in identification and classification of *GmVQ* genes. Further experimental analysis will allows us to know the functions of *GmVQs* participation in plant growth and stress responses.

## Introduction

*VQ* genes are plant specific genes, which involved in plant development and multiple stress responses (Cheng et al., 2012). A conserved amino acid region has been identified within them, which composed of approximately 50–60 amino acids with a highly conserved the FxxhVQxhTG motif (Jing & Lin, 2015). The *VQ* domain possesses multiple biological functions in *VQ* proteins, such as the mutant strain of *AtVQ14* (changes from IVQQ to EDLE) in the *VQ* domain result in producing small seeds, nevertheless the mutations in other locations does not have this characteristic (Wang et al., 2010). Furthermore, studies have reported that *VQ* genes are different in plants and do not have any intron in higher plants, whereas most *VQ* genes contain one or more introns in moss (Li et al., 2014; Jiang, Sevugan & Ramachandran, 2018; Dong et al., 2018).*VQ* proteins can interact with the WRKY proteins, for example, SIB1 and SIB2 are also *VQ* proteins, they were interacted with WRKY33 by recognizing the *WRKY* domain in C-terminal to activating the defense of plants (Lai et al., 2011).

*VQ* proteins were reported in dicotyledon such as *Arabidopsis thaliana* (Cheng et al., 2012), *Vitis vinifera* (Wang et al., 2015), *Camellia sinensis* (Guo et al., 2018), and monocotyledon such as *Oryza sativa* (Kim et al., 2013a; Kim et al., 2013b), *Zea mays* (Song et al., 2016)*. VQ* proteins perform a variety of functions in plant development. For example, IKU1 (AT2G35230) is one of the VQ protein, it involved in regulating endosperm development and affect the seed formation during plant growth (Garcia & Berger, 2003). Under the far-red and low intensity of white light conditions, over expression of *AtVQ29* can reduces the hypocotyl growth and it has higher expression in stem cells (Perruc et al., 1999). Furthermore, *VQ* genes regulate varying functions under abiotic and biotic stresses. *AtCaMBP25* (also named *AtVQ15*) overexpression in transgenic plants had highly sensitive to osmotic stress in germination and early growth of seeds (Perruc et al., 1999). *AtVQ9* alleviated the activity of *WRKY8* under salt stress** (Hu et al., 2013). The transcript levels of *AtVQ23* and *AtVQ16* are strongly induced by *Botrytis cinerea* infection and SA stress (Lai et al., 2011).

*Glycine max* is an important economic crop, widely cultivated in a number of countries. They are often subjected to abiotic stresses during the growth process, such as drought, high salinity, and other abiotic stresses were severely influenced on soybean production (Liu & Li, 2010). Therefore, identification of resistance genes has great significance for improving the yield and quality of soybean through molecular breeding. In this study, we identified 75 *VQ* genes of the soybean genome, and analyzed their phylogenetic, evolutionary motif, structure, promoter, and expression pattern. In addition, we analyzed the *GmVQs*’s expression level in different multiple abiotic stresses. Our results provide a basic information on identification and classification of *GmVQ* genes, and further experimental analysis allows us to comprehend the functions of *GmVQs* participate in plant growth and stress responses.

## Materials & Methods

### Identification of *VQ* genes

The Hidden Markov Model (HMM) profiles of the *VQ* motif PF05678 were downloaded from the Pfam database (Punta et al., 2012). HMM searched VQ motif (PF05678) from the *G. max* proteins database with the values (e-value) cut-off at 0.1 (Punta et al., 2012). The integrity of the *VQ* motif was determined using the online program SMART (http://smart.embl-heidelberg.de/) with an e-value < 0.1 (Letunic, Doerks & Bork, 2012). In addition, the three fields (length, molecular weight, and isoelectric point) of each VQ protein were predicted by the online ExPasy program (http://www.expasy.org/tools/) (Rueda et al., 2015).

### Phylogenetic analysis

To investigate the phylogenetic relationship of the *VQ* gene families among *A. thaliana*, *O. sativa*, and *G. max*, AtVQ and OsVQ proteins were downloaded from phytozomes (http://www.phytozome.org) based on the previous studies (Cheng et al., 2012; Li et al., 2014; Goodstein et al., 2012). VQ proteins were aligned using the BioEdit program. A neighbor-joining (NJ) phylogenetic tree was constructed using these proteins through MEGA7.0 software (Tamura et al., 2011). Bootstrapping was performed with 1,000 replications. Genes were classified according to the distance homology with *A. thaliana* and *O. sativa* genes (Cheng et al., 2012; Li et al., 2014).

### Sequence alignment, motif prediction and gene structure of *GmVQ* genes

Multiple alignments of the VQ full length proteins were conducted using Jalview software with default parameter settings. The online MEME analysis used to identify the unknown conserved motifs (http://meme.ebi.edu.au/meme/intro.html) using the following parameters: site distribution: zero or one occurrence (of a contributing motif site) per sequence, maximum number of motifs: 20, and optimum motif width ≥6 and ≤200 (Bailey et al., 2015). A gene structure displaying server program (http://gsds.cbi.pku.edu.cn/index.php) was used to show the structure of *Glycine max VQ* gene.

### Gene duplication and collinearity analysis

The physical locations of the *GmVQ* genes on the soybean chromosomes were mapped by using MG2C website (http://mg2c.iask.in/mg2c_v2.0/). The analysis of synteny among the soybean genomes was conducted locally using a method similar to the one developed for the PGDD (http://chibba.agtec.uga.edu/duplication/) (Krzywinski et al., 2009). First, BLASTP, OrthoMCL software (http://orthomcl.org/orthomcl/about.do#release) and MCScanX software (Wang et al., 2012) were used to search for potential homologous gene pairs (E < 1 e^−5^, top five matches) across multiple genomes. Then, these homologous pairs were used as the input for the PGDD database (http://chibba.agtec.uga.edu/duplication/). Ideograms were created using Circos (Krzywinski et al., 2009).

### Calculating *Ka* and *Ks*

The *Ka* and *Ks* were used to assess the selection history and divergence time of gene families (Li, Gojobori & Nei, 1981). The number of synonymous (*Ks*) and nonsynonymous (*Ka*) substitutions of duplicated *VQ* genes was computed by using the KaKs_Calculator 2.0 with the NG method (Xu et al., 2018). The divergence time (*T*) was calculated using the formula *T* = *Ks*∕(2 × 6.1 × 10^−9^) × 10^−6^ million years ago (MYA) (Kim et al., 2013a; Kim et al., 2013b).

### *VQ* genes expression analysis of soybean

The expression data of *VQ* genes in different tissues, including seed, pod, SAM, stem, flower, leaf, root, root hair and nodule, is available in Phytozome V12.1 database (https://phytozome.jgi.doe.gov/pz/portal.html). The expression profile for *VQ* genes was utilized for generating the heatmap and k-means clustering using R 3.2.2 software (Gentleman et al., 2004).

### Plant material and treatments

*Glycine max* (Williams 82) was used in this study. Seeds were planted in a 3:1 (w/w) mixture of soil and sand, germinated, and irrigated with half-strength Hoagland solution once every 2 days. The seedlings were grown in a night temperature of 20 °C and day temperature of 22 °C, relative humidity of 60 %, and a 16/8 h photoperiod (daytime: 05:00–21:00). After 4 weeks, the germinated seedlings were treated with 20% PEG6000 (drought), 250 mM NaCl solution (salt), 4 °C (cold), 100 µM abscisic acid (ABA), 100 µM salicylic acid (SA) solutions. Control and treated seedlings were harvested 1 h, 6 h, 12 h, and 24 h after treatment. All samples were frozen in liquid nitrogen and stored at −80 °C until use.

### RNA extraction and Quantitative real-time PCR (qRT-PCR)

Total RNA was extracted from *G. max* using RNAiso Plus (TaKaRa, Tokyo, Japan) according to manufacturer’s instructions. The cDNA synthesis was carried out with approximately 2 µg RNA using PrimeScript RT reagent Kit with gDNA Eraser (TaKaRa, Tokyo, Japan). Quantitative Real-time PCR (qRT-PCR) was performed using SYBR *Premix Ex Taq* II (TaKaRa, Tokyo, Japan) on an ABI Prism 7000 sequence detection system (Applied Biosystems, USA) with the primers listed in Table S1. PCR amplification was performed in accordance with SYBR *Premix Ex Taq* (TaKaRa, Tokyo, Japan) response system. For each sample, three technical replicates were conducted to calculate the averaged Ct values. Relative expression was calculated by the 2^−ΔΔ*Ct*^ method (Livak & Schmittgen, 2001). The actin and GAPDH genes were used as internal control.

### Gene Ontology Enrichment

Once the sequences were obtained ran a BLASTX search against the UNIPROT database at a 1e-30 significance level. The matches were extracted and compared to the GO annotation generated against UNIPROT hits located at EBI. The GO annotation of the GmVQ genes by using WEGO 2.0 website (http://wego.genomics.org.cn/).

### Analyzed the *cis*-elements of *GmVQ* promoters

The *cis*-elements of *GmVQ* promoters were analyzed to further understand the *GmVQ* gene family. We examined the sequences within 1,500 base pairs (bp) upstream of initiation codons (ATG) for promoter analysis and searched for these sequences in the soybean genome. The *cis*-elements in promoters were subsequently searched using the PlantCARE database (http://bioinformatics.psb.ugent.be/webtools/plantcare/html/).

### Gene interaction network

Protein sequence of *GmWRKY* transcription factors were obtained from the genome database of soybean, also were mapped to the WRKY proteins of Arabidopsis by BLASTP tool in the TAIR database. Subsequently, the interaction between *GmVQs* and *GmWRKYs* were forecasted based on the PAIR website (https://rc.webmail.pair.com/), and their network was drawn in Cytoscape 3.6.1.

## Results

### Identification of *GmVQs*

Hidden Markov Model (HMM) of the *VQ* motif (PF05678) was used to search for putative VQs in soybean proteins database. A total of 75 *VQs* were identifiedand were named from *GmVQ1* to *GmVQ75* based on their physical locations on the chromosomes. This is different from the previous study, which 74 *GmVQs* were identified before the database updated (Wang et al., 2014; Zhou et al., 2016). ExPasy predicted that these 75 VQ proteins have different physical and chemical properties whose amino acid lengths ranged from 89 aa (*GmVQ37*) to 486 aa (*GmVQ18*), with an average of 223 aa and most of them were less than 300 aa. The molecular weights of these 75 VQ proteins ranged from 10.03 kDa (*GmVQ37*) to 52.79 kDa (*GmVQ18*) and their isoelectric points ranged from 4.29 (*GmVQ69*) to 10.74 (*GmVQ51*) (Table 1).

**Table 1:** List of all GmVQ genes identified in the *Glycine max* genome.

Gene name	Gene locus	Chromosome location	Length (aa)	pI	Molecular weight (Da)	Family group
GmVQ1	Glyma01G018700	chr1:1790049-1792039	318	10.66	34712.78	VII
GmVQ2	Glyma01G096800	chr1:31515839-31517715	289	10.24	31636.98	VII
GmVQ3	Glyma01G195300	chr1:52952165-52953181	154	9.48	16878.08	VII
GmVQ4	Glyma02G208800	chr2:39393500-39394691	212	9.96	23346.3	VII
GmVQ5	Glyma03G120700	chr3:33242128-33243660	233	7.79	24442.59	VI
GmVQ6	Glyma03G127800	chr3:34231323-34232268	167	4.79	18699.76	I
GmVQ7	Glyma03G204900	chr3:41299415-41300202	119	9.84	13358.41	II
GmVQ8	Glyma03G249100	chr3:44529232-44529956	127	9.11	14693.84	I
GmVQ9	Glyma04G099600	chr4:9115245-9116947	287	10.12	31433.71	VII
GmVQ10	Glyma04G103200	chr4:9567274-9568529	205	9.14	22493.86	V
GmVQ11	Glyma04G103300	chr4:9570059-9571000	313	6.64	34199.48	V
GmVQ12	Glyma04G134200	chr4:19214276-19215243	127	6.7	14519.25	I
GmVQ13	Glyma04G214700	chr4:48626650-48627708	212	5.9	22930.94	II
GmVQ14	Glyma04G239400	chr4:50786868-50788346	240	8.96	26255.85	II
GmVQ15	Glyma05G107500	chr5:28551166-28551996	186	8.48	20503.94	VII
GmVQ16	Glyma05G133000	chr5:32592583-32593521	211	7.79	23513.49	V
GmVQ17	Glyma05G140700	chr5:33359443-33360263	113	5.43	12313.85	III
GmVQ18	Glyma05G179700	chr5:36744975-36747458	486	6.12	52793.72	V
GmVQ19	Glyma05G190000	chr5:37570564-37571544	208	6.84	22399.46	II
GmVQ20	Glyma05G198400	chr5:38262465-38265127	186	9.52	20618.64	IV
GmVQ21	Glyma06G101400	chr6:8043143-8044571	295	10.28	32310.83	VII
GmVQ22	Glyma06G104400	chr6:8309457-8311289	341	6.06	37163.76	V
GmVQ23	Glyma06G104500	chr6:8314638-8315685	316	6.48	34579.04	V
GmVQ24	Glyma06G124400	chr6:10128263-10129012	249	8.11	27136.69	II
GmVQ25	Glyma06G151400	chr6:12350255-12351217	222	5.97	24195.35	II
GmVQ26	Glyma06G240300	chr6:39620687-39622549	244	7.79	26839.89	IV
GmVQ27	Glyma07G028700	chr7:2307932-2309158	193	9.98	20948.74	IV
GmVQ28	Glyma07G092500	chr7:8632559-8633302	247	5.97	27087.07	II
GmVQ29	Glyma07G198000	chr7:36647302-36650373	310	8.42	33741.87	IV
GmVQ30	Glyma08G005700	chr8:456890-461071	174	9.1	19216.73	IV
GmVQ31	Glyma08G041900	chr8:3320834-3322022	140	6.9	15589.54	II
GmVQ32	Glyma08G087400	chr8:6616587-6617687	221	6.91	24350.54	V
GmVQ33	Glyma08G096000	chr8:7331414-7331749	111	6.26	12060.59	III
GmVQ34	Glyma08G137300	chr8:10495440-10498069	472	6.33	51419.34	V
GmVQ35	Glyma08G147600	chr8:11258747-11259761	198	6.51	21201.12	II
GmVQ36	Glyma08G157900	chr8:12235183-12236400	141	5.63	15964.98	III
GmVQ37	Glyma08G176500	chr8:14151104-14151373	89	7.89	10029.37	III
GmVQ38	Glyma08G214100	chr8:17287863-17288952	194	9.69	21099.11	IV
GmVQ39	Glyma08G272000	chr8:35627645-35629488	292	10.39	32000.2	VII
GmVQ40	Glyma08G272100	chr8:35632723-35638206	361	9.8	39876.98	VII
GmVQ41	Glyma08G272200	chr8:35665488-35667249	299	10.24	32844.1	VII
GmVQ42	Glyma08G308400	chr8:42711855-42712403	182	4.3	20461.87	VII
GmVQ43	Glyma09G051900	chr9:4508892-4509626	244	6.48	27252.83	V
GmVQ44	Glyma09G111800	chr9:22128197-22129301	203	7.11	22686.53	III
GmVQ45	Glyma09G183700	chr9:40881519-40882250	243	6.13	26618.56	II
GmVQ46	Glyma10G273300	chr10:49575568-49576678	191	7.83	20981.58	II
GmVQ47	Glyma11G046400	chr11:3468797-3469599	155	9.16	16952.31	VII
GmVQ48	Glyma11G239600	chr11:33399330-33401730	439	7.02	47710.64	V
GmVQ49	Glyma12G153600	chr12:23455875-23457485	248	7.02	27499.55	IV
GmVQ50	Glyma12G225200	chr12:38479959-38482769	246	7.17	27184.17	IV
GmVQ51	Glyma13G005100	chr13:1422443-1424046	224	10.74	23979.57	V
GmVQ52	Glyma13G039800	chr13:12310527-12311616	240	10.11	25791.06	II
GmVQ53	Glyma13G178500	chr13:29211898-29216138	281	9.8	30474.04	IV
GmVQ54	Glyma13G193800	chr13:30709903-30710253	116	5.14	13478	I
GmVQ55	Glyma13G218400	chr13:33181476-33181778	100	9.05	11045.26	III
GmVQ56	Glyma13G238100	chr13:34835450-34840141	260	9.54	27989.72	IV
GmVQ57	Glyma13G276100	chr13:37756820-37759545	249	7.91	27358.41	IV
GmVQ58	Glyma14G002800	chr14:293552-294736	161	9.68	17490.39	V
GmVQ59	Glyma14G124800	chr14:19432507-19433220	237	8.85	25366.52	II
GmVQ60	Glyma14G172200	chr14:42617341-42619795	429	6.59	46407.55	V
GmVQ61	Glyma15G075200	chr15:5769826-5772617	199	9.77	21595.31	IV
GmVQ62	Glyma15G158200	chr15:13251793-13252987	252	7.16	27774.66	V
GmVQ63	Glyma15G232200	chr15:43662201-43662912	122	6.65	14310.95	I
GmVQ64	Glyma15G249800	chr15:47637825-47638440	89	7.89	10074.51	III
GmVQ65	Glyma15G268300	chr15:50482677-50484349	158	7.74	17892.46	III
GmVQ66	Glyma17G159600	chr17:13790434-13791675	190	9.3	21149.77	VII
GmVQ67	Glyma17G182600	chr17:22616386-22616835	149	9.27	16873.05	V
GmVQ68	Glyma18G017800	chr18:1285879-1287658	454	6.45	48264.96	V
GmVQ69	Glyma18G108600	chr18:12426789-12427328	179	4.29	20004.4	VII
GmVQ70	Glyma19G125300	chr19:38346007-38347425	232	9.64	24435.6	VI
GmVQ71	Glyma19G130400	chr19:39031134-39032107	168	5.16	18878.02	I
GmVQ72	Glyma19G202300	chr19:45923283-45923992	124	9.7	13533.53	II
GmVQ73	Glyma19G246700	chr19:49331581-49332332	102	9.19	11775.23	I
GmVQ74	Glyma20G064500	chr20:21930212-21930913	233	10.51	25070.57	V
GmVQ75	Glyma20G116600	chr20:35927408-35928282	157	5.9	17333.32	II

### Phylogenetic analysis and multiple alignment of the *VQ* genes

**Figure 1:**
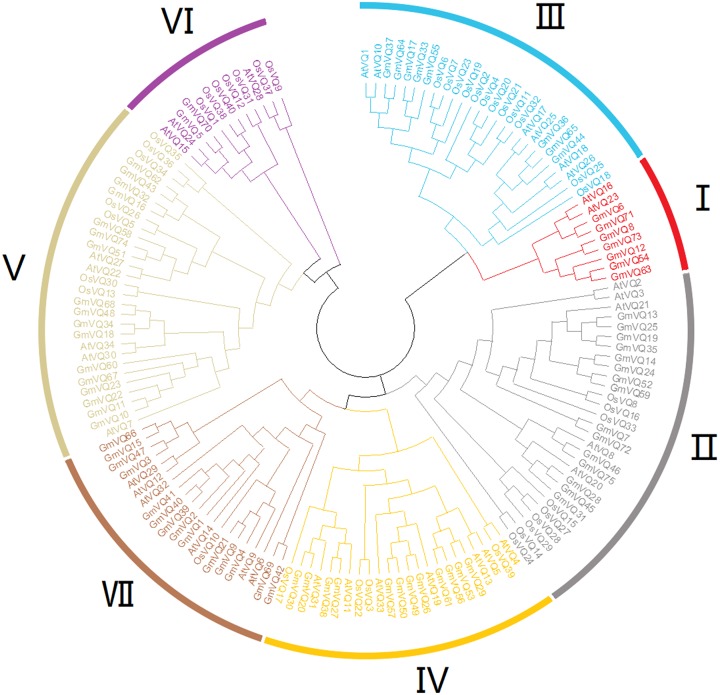
Phylogenetic tree analysis of the VQ genes in *Glycine max, Arabidopsis thaliana* and *Oryza sativa*. The phylogenetic tree was constructed using MEGA 7.0 by the neighbor-joining method. The Bootstrap value was 1,000 replicates. The three plant-specific clusters were designated as group I-VII and indicated in a specific color.

**Figure 2:**
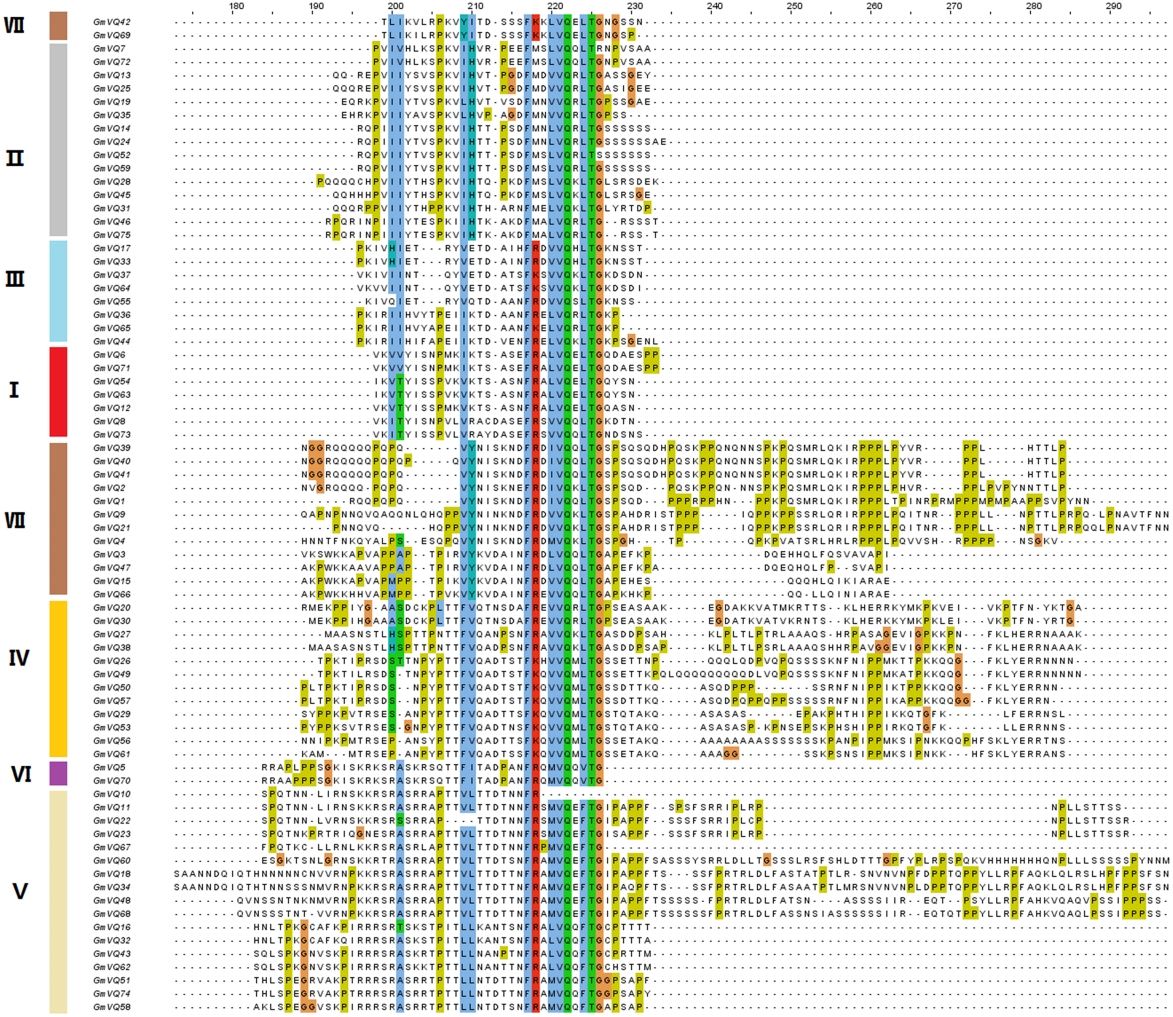
Multiple sequence alignment, gene structure and multiple motifs of soybean. Alignment of *VQ* domain of 75 VQ proteins in soybean. Amino acids that are conserved throughout are shaded in different colors. The genes in different groups are in different colors.

To explore the phylogenetic relationships among the *VQ* genes of soybean, *A. thaliana* and *O. sativa*, a NJ phylogenetic tree was constructed (Fig. 1). We found that soybean and *A. thaliana* have a closer relationship than rice. Based on their relationship with** AtVQs** and OsVQs and the characteristics of GmVQs’ core domain, they were divided into 7 groups, designated Group I-VII (Figs. 1 and 2). For the 75 GmVQ proteins, Group VI contains two VQ proteins; Group V has the biggest amount, with 17 VQ proteins. Groups I, II, III, IV, VII contain 7, 15, 8, 12, 14 members respectively. At the same time, we found 5 types of *VQ* specificity domain: FxxxVQxLTG (54/75), FxxxVQxFTG (16/75), FxxxVQxVTG (2/75), FxxxVQxLTR (1/75), FxxxVQxLTS (1/75), besides, there is also a GmVQ protein (GmVQ10) has partial domain deletion (Fig. 2). Different types of *VQ* domains indicate that they might have different biological functions.

### Conserved motifs and gene structures of the *VQ* gene family

**Figure 3:**
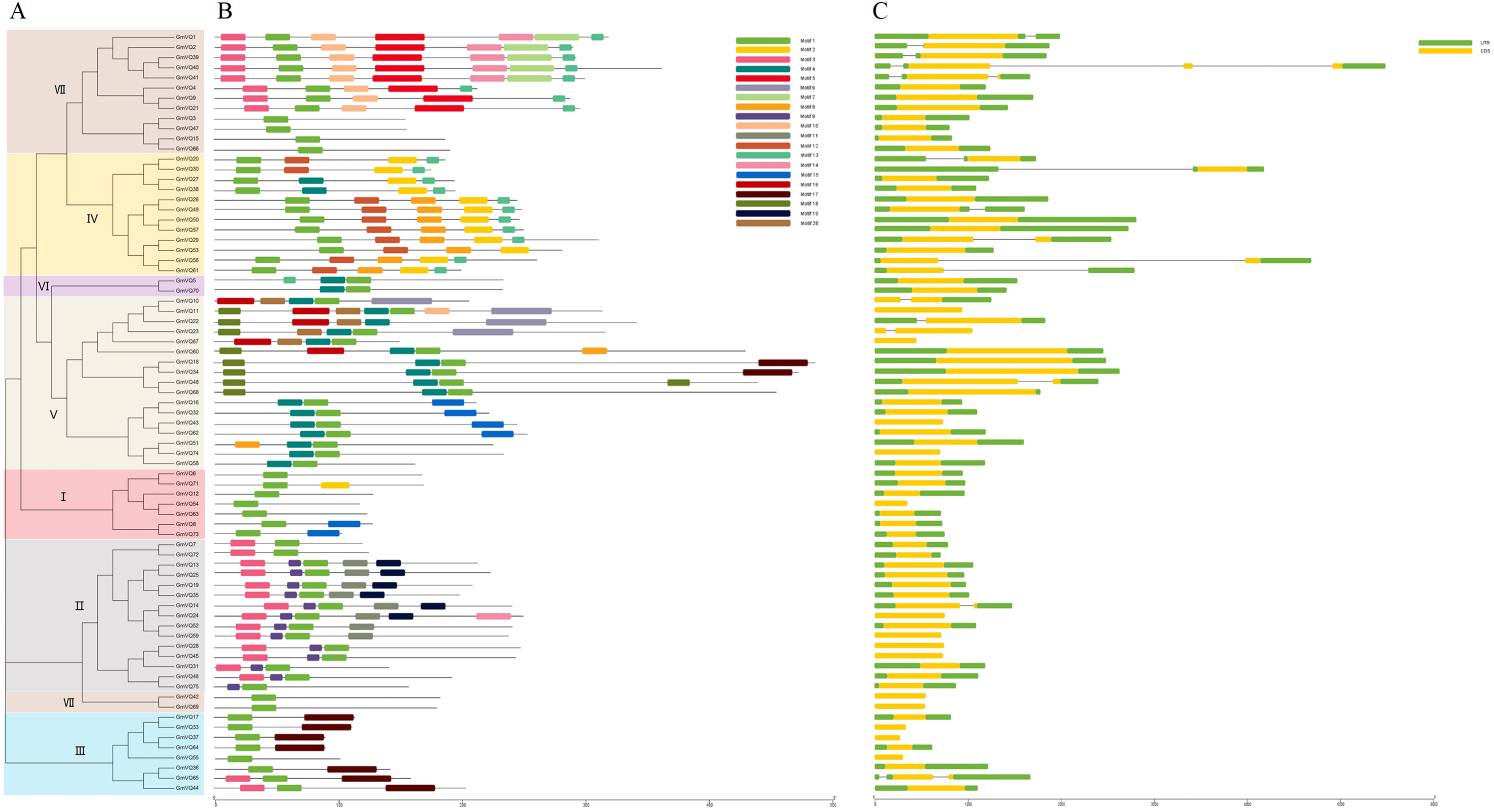
Phylogenetic tree, conserved motifs and gene structure in GmVQs. (A) Phylogenetic relationships (B) Conserved motifs of the *GmVQ*s. Each motif is represented by a number in colored box. (C) Exon/intron structures of *GmVQ* genes.

We predicted that the 75 *GmVQs* contained 20 conserved motifs, with the motif length ranged from 11 aa to 50 aa (Fig. S1). Every *GmVQ* member contains 1-7 conserved motifs (Fig. 3B). All of the proteins, excepted *GmVQ22*, show motif 1 which contains a specialty *VQ* domain. Additionally, an unrooted phylogenetic tree was constructed with *VQ* protein sequences, suggested that the motifs organization of *VQ* genes were consistent with the phylogenetic tree (Fig. 3A). Group V contains motif 4, Group IV contains motif 2. We found that most groups possess more than two motifs, suggested that every group might have special functions with a highly conserved amino acid residue. Through the *VQ* gene structures analysis, half of the group VI has introns; genes in group V have longer coding regions, while genes of group I have shorter coding regions than other groups (Fig. 3C). Interestingly, 78.67% (59/75) of *GmVQ* genes are intronless genes. It is speculated that a large number of introns might be lost in *VQ* genes during evolution. The phylogenetic tree shows that genes from same branches have similar gene structures, while those from different branches have different gene structures (Fig. 3A).

### Chromosome location and gene duplication

**Figure 4:**
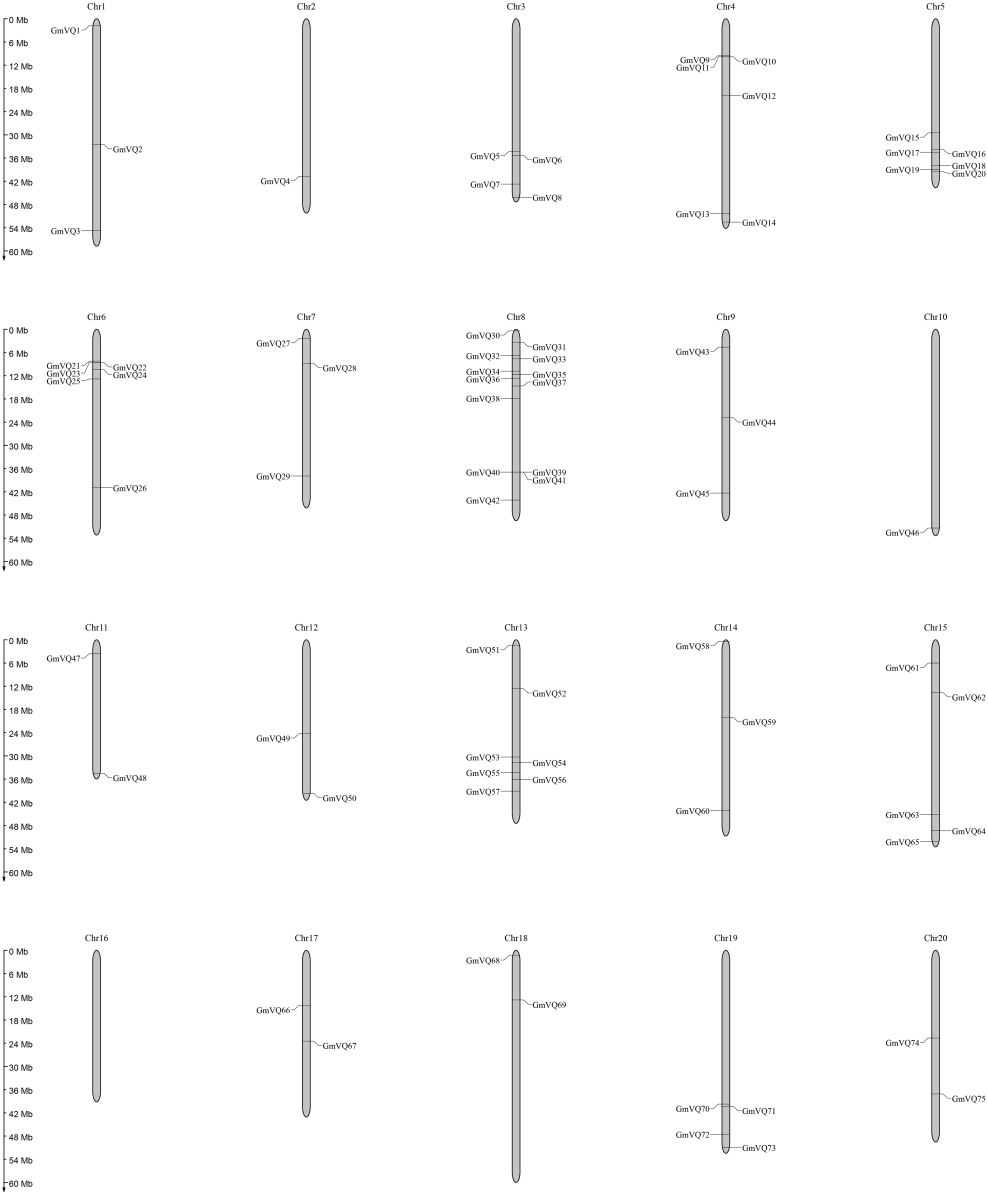
Chromosome location and duplication events analysis in *Glycine max*.

We drew a chromosomal location map of *GmVQ* genes on each chromosome (Fig. 4). *GmVQs* are distributed on all soybean chromosomes, except chromosome 16, and were densely distributed on chromosome 8 and chromosome 13, containing 13 and 7 members, respectively (Fig. 4). Most of *GmVQs* are distributed on the two ends of chromosomes.

Segmental or tandem duplicate in many gene families are the main expanding way in plants. To better study the evolution of *GmVQ* genes, we further explored gene duplication events using the MCScanX software. We found that 52 pairs of genes originated from segmental duplication, and 4 pairs of genes involved in tandem duplication events (Table S2).

### Evolution and divergence of the *VQ* gene family in soybean and *Arabidopsis*

With the OrthoMCL software, we found 56 paralogous pairs in soybean, 37 orthologous pairs between soybean and Arabidopsis. Some *VQ* genes have never had any homology genes. All the paralogous and orthologous pairs are listed in Table 2. At the same time, we found that two or more *GmVQ* genes match to one *AtVQ* gene, implying that they might promote the expansion of the *VQ* gene family during evolution. We calculated Ka/Ks ratios of 55 paralogous pairs in soybean (Table 3). Most Ka/Ks ratios are <1, however, the *GmVQ54/GmVQ63* and *GmVQ65/GmVQ36* pairs are <1. In addition, the genetic differentiation of the 55 gene pairs occurred between 5 and 30 MYA.

**Table 2:** Paralogous (Gm-Gm) and orthologous (Gm-At) gene pairs.

Gm-Gm	Gm-Gm	Gm-At
GmVQ3/GmVQ47	GmVQ24/GmVQ59	GmVQ37/AtVQ1
GmVQ5/GmVQ70	GmVQ27/GmVQ38	GmVQ64/AtVQ1
GmVQ6/GmVQ71	GmVQ28/GmVQ45	GmVQ14/AtVQ3
GmVQ7/GmVQ72	GmVQ29/GmVQ53	GmVQ24/AtVQ3
GmVQ8/GmVQ73	GmVQ29/GmVQ61	GmVQ52/AtVQ3
GmVQ9/GmVQ21	GmVQ29/GmVQ56	GmVQ59/AtVQ3
GmVQ10/GmVQ11	GmVQ34/GmVQ68	GmVQ29/AtVQ5
GmVQ10/GmVQ22	GmVQ34/GmVQ48	GmVQ53/AtVQ5
GmVQ10/GmVQ23	GmVQ37/GmVQ64	GmVQ61/AtVQ5
GmVQ10/GmVQ67	GmVQ39/GmVQ40	GmVQ56/AtVQ5
GmVQ11/GmVQ22	GmVQ39/GmVQ41	GmVQ46/AtVQ8
GmVQ11/GmVQ23	GmVQ39/GmVQ2	GmVQ75/AtVQ8
GmVQ11/GmVQ67	GmVQ40/GmVQ41	GmVQ9/AtVQ9
GmVQ13/GmVQ25	GmVQ40/GmVQ2	GmVQ21/AtVQ9
GmVQ14/GmVQ24	GmVQ41/GmVQ2	GmVQ37/AtVQ10
GmVQ14/GmVQ52	GmVQ42/GmVQ69	GmVQ64/AtVQ10
GmVQ14/GmVQ59	GmVQ43/GmVQ62	GmVQ27/AtVQ11
GmVQ15/GmVQ66	GmVQ46/GmVQ75	GmVQ38/AtVQ11
GmVQ16/GmVQ32	GmVQ49/GmVQ26	GmVQ1/AtVQ14
GmVQ18/GmVQ34	GmVQ50/GmVQ57	GmVQ5/AtVQ15
GmVQ18/GmVQ68	GmVQ51/GmVQ74	GmVQ70/AtVQ15
GmVQ18/GmVQ48	GmVQ52/GmVQ59	GmVQ44/AtVQ17
GmVQ19/GmVQ35	GmVQ53/GmVQ61	GmVQ50/AtVQ19
GmVQ20/GmVQ30	GmVQ53/GmVQ56	GmVQ57/AtVQ19
GmVQ22/GmVQ23	GmVQ54/GmVQ63	GmVQ28/AtVQ20
GmVQ22/GmVQ67	GmVQ61/GmVQ56	GmVQ45/AtVQ20
GmVQ23/GmVQ67	GmVQ65/GmVQ36	GmVQ19/AtVQ21
GmVQ24/GmVQ52	GmVQ68/GmVQ48	GmVQ35/AtVQ21
		GmVQ5/AtVQ24
		GmVQ70/AtVQ24
		GmVQ44/AtVQ25
		GmVQ20/AtVQ31
		GmVQ30/AtVQ31
		GmVQ18/AtVQ34
		GmVQ34/AtVQ34
		GmVQ68/AtVQ34
		GmVQ48/AtVQ34

**Table 3:** Ka, Ks and Ka/Ks values calculated for paralogous VQ gene pairs.

Gene 1	Gene 2	Ka	Ks	Ka/Ks ratio
GmVQ10	GmVQ11	0.002146692	0.013590406	0.15795643
GmVQ39	GmVQ40	0.014329244	0.032130119	0.445975451
GmVQ54	GmVQ63	0.070550548	0.066958938	1.053638991
GmVQ65	GmVQ36	0.129398947	0.090241867	1.433912564
GmVQ39	GmVQ41	0.015091052	0.092583143	0.163
GmVQ37	GmVQ64	0.033904078	0.095366382	0.355513935
GmVQ40	GmVQ41	0.02957328	0.096785754	0.305554062
GmVQ42	GmVQ69	0.039057024	0.110714815	0.352771436
GmVQ16	GmVQ32	0.056833182	0.117015316	0.485690114
GmVQ40	GmVQ2	0.056768509	0.119061292	0.476800713
GmVQ50	GmVQ57	0.012662345	0.127110953	0.099616473
GmVQ49	GmVQ26	0.034291687	0.128532842	0.26679319
GmVQ43	GmVQ62	0.057675771	0.129647567	0.444865819
GmVQ19	GmVQ35	0.060650867	0.132943691	0.456214704
GmVQ20	GmVQ30	0.047369299	0.134768712	0.351485879
GmVQ7	GmVQ72	0.073853375	0.1352768	0.545942653
GmVQ39	GmVQ2	0.050072412	0.136741168	0.366183886
GmVQ18	GmVQ34	0.048240383	0.141323284	0.341347734
GmVQ68	GmVQ48	0.072358564	0.146626964	0.493487433
GmVQ3	GmVQ47	0.082715722	0.153202253	0.539911912
GmVQ5	GmVQ70	0.053173881	0.15994977	0.33244112
GmVQ46	GmVQ75	0.053993084	0.162730183	0.33179514
GmVQ41	GmVQ2	0.047558234	0.164392307	0.289297198
GmVQ10	GmVQ22	0.080947528	0.167701359	0.482688565
GmVQ22	GmVQ23	0.12358775	0.173118789	0.713889871
GmVQ51	GmVQ74	0.051867494	0.17525146	0.295960409
GmVQ27	GmVQ38	0.068641212	0.187083237	0.366901992
GmVQ8	GmVQ73	0.07651417	0.194357737	0.393676994
GmVQ15	GmVQ66	0.064674193	0.201500105	0.32096357
GmVQ9	GmVQ21	0.031810279	0.204712416	0.155390081
GmVQ11	GmVQ22	0.068482759	0.20561255	0.33306702
GmVQ28	GmVQ45	0.07428748	0.212707642	0.349246879
GmVQ13	GmVQ25	0.0495425	0.21323249	0.232340297
GmVQ61	GmVQ56	0.078572076	0.228081862	0.344490682
GmVQ11	GmVQ67	0.187484416	0.242381253	0.773510383
GmVQ14	GmVQ24	0.070535521	0.260458812	0.270812571
GmVQ11	GmVQ23	0.143077885	0.278054006	0.514568687
GmVQ10	GmVQ67	0.25396485	0.28526939	0.890263236
GmVQ6	GmVQ71	0.098963625	0.304675089	0.324816923
GmVQ22	GmVQ67	0.242648421	0.35967981	0.674623413
GmVQ29	GmVQ53	0.1121903	0.365440906	0.306999841
GmVQ10	GmVQ23	0.206060196	0.446610645	0.461386664
GmVQ18	GmVQ48	0.431966031	0.855432366	0.50496807
GmVQ18	GmVQ68	0.415845352	0.884745464	0.470016936
GmVQ34	GmVQ68	0.40356234	0.937434864	0.430496406
GmVQ34	GmVQ48	0.385776641	0.968352827	0.398384381
GmVQ29	GmVQ61	0.320802002	0.987438675	0.324882963
GmVQ24	GmVQ59	0.231784771	1.001714144	0.231388139
GmVQ14	GmVQ59	0.271393455	1.079747859	0.251348917
GmVQ23	GmVQ67	0.564635744	1.087320132	0.51929117
GmVQ53	GmVQ61	0.281138217	1.090000891	0.257924759
GmVQ29	GmVQ56	0.422850584	1.281581327	0.329944401
GmVQ53	GmVQ56	0.280639233	1.337494009	0.209824665
GmVQ14	GmVQ52	1.027115432	1.538918692	0.667426705
GmVQ24	GmVQ52	1.074280372	1.754750076	0.612212751

### Expression analysis of *GmVQ* genes among various tissues

**Figure 5:**
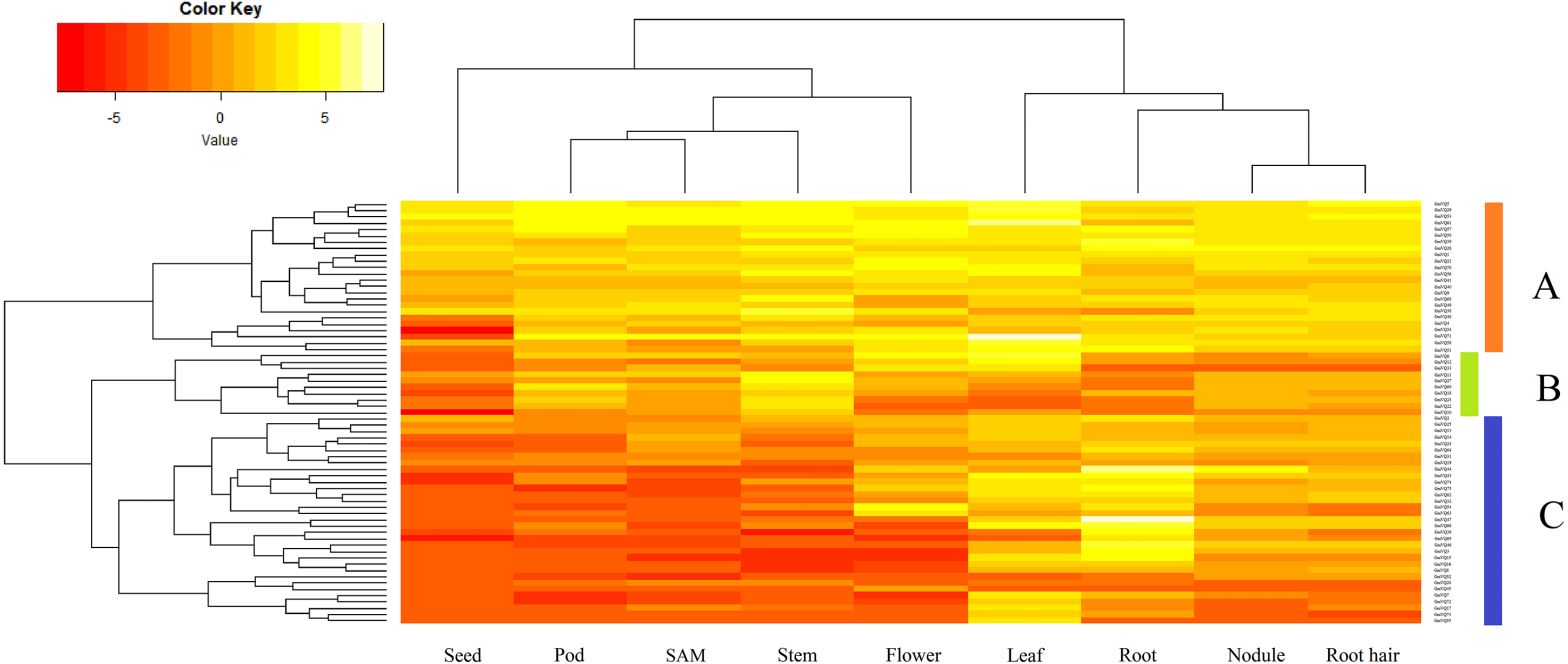
Expression analysis of *GmVQ* genes in different tissues and different stages. The clusters were designated as group A-C. Different colors in map represent gene transcript abundance values as shown in bar at top of figure.

Sixty-seven *GmVQ* genes were investigated using available RNA-seq data from nine different tissues (seed, pod, SAM, stem, flower, leaf, root, nodule, and root hair) (Fig. 5). We found that the expression levels of the *GmVQs* varied significantly in different tissues.Most *GmVQ* genes were found expressed in more than one detected organ. As shown in Fig. 5, genes in group A are expressed in all analyzed tissues. The expression levels of group B in pod and stem tissues are higher. Genes in group C have specific expression in leaf and root.

### Expression patterns of *GmVQs* under abiotic stress

**Figure 6:**
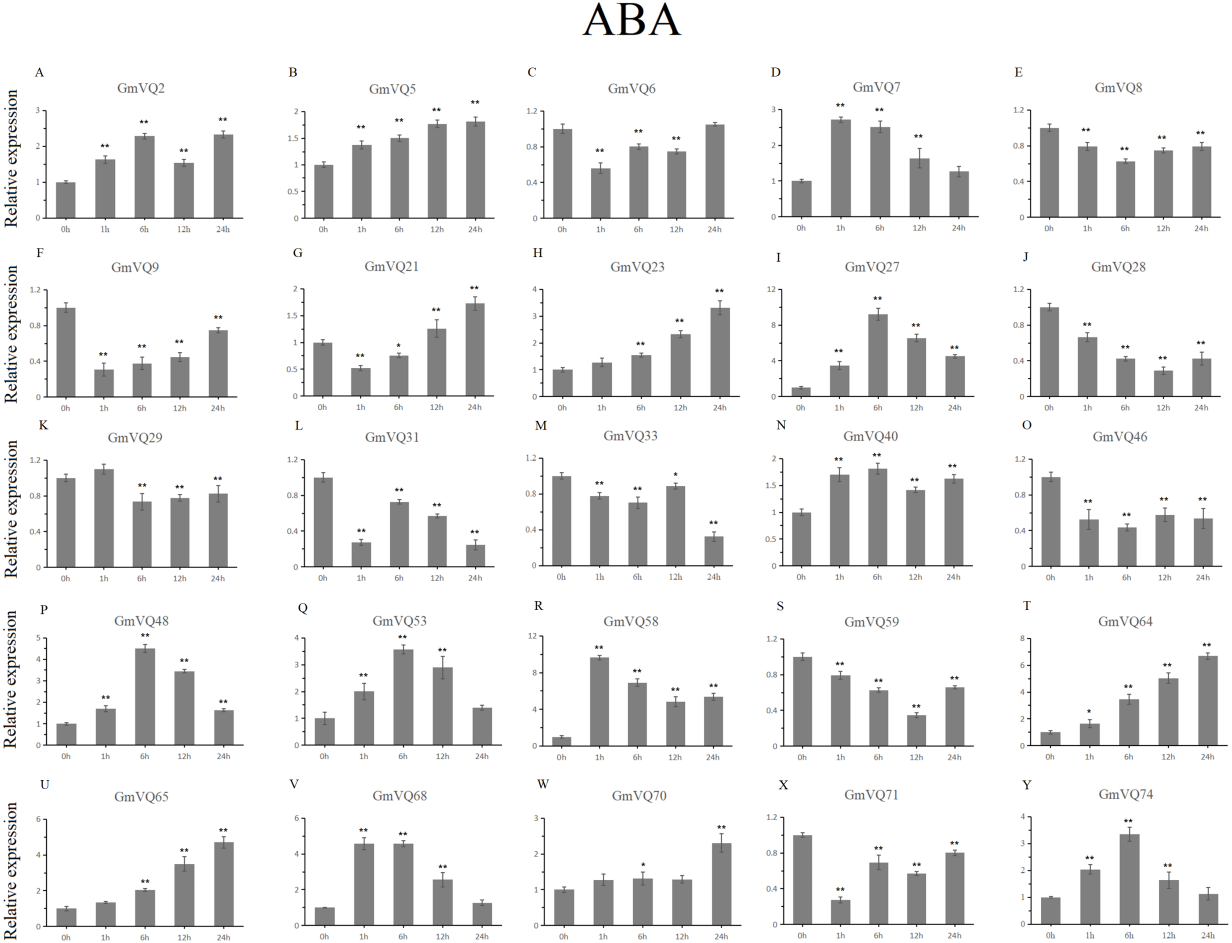
qRT-PCR analysis reveals *GmVQ* genes under ABA treatment compared to the controls. Stress treatments and time course are described in the Materials and Methods section. (A-Y) represent different genes which were used in qRT-PCR analysis. Asterisks on top of the bars indicating statistically significant differences between the stress and counterpart controls (*p < 0.05, **p < 0.01).

**Figure 7:**
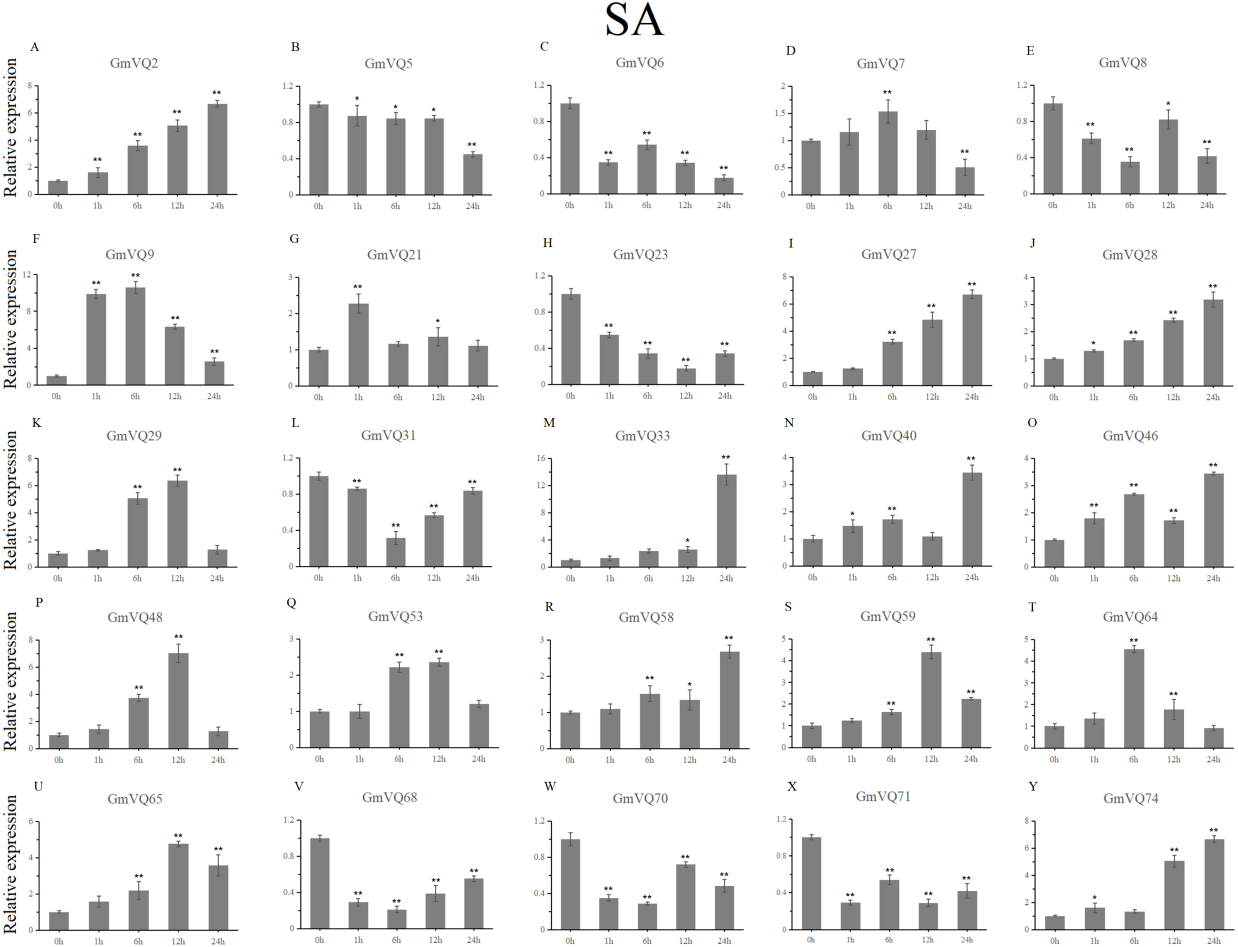
qRT-PCR analysis reveals *GmVQ* genes under SA treatment compared to the controls. Stress treatments and time course are described in the Materials and Methods section. (A-Y) represent different genes which were used in qRT-PCR analysis.Asterisks on top of the bars indicating statistically significant differences between the stress and counterpart controls (*p < 0.05, **p < 0.01).

**Figure 8:**
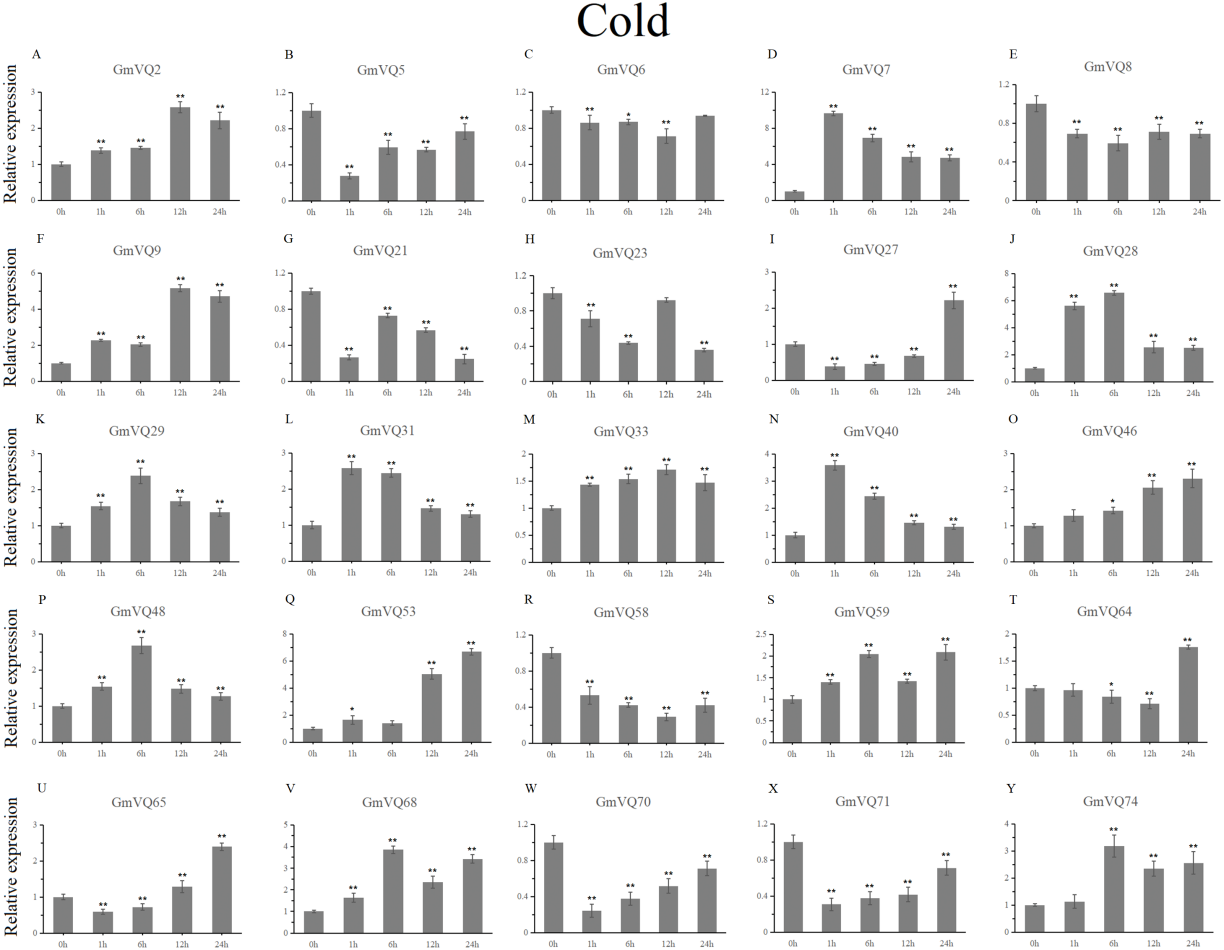
qRT-PCR analysis reveals *GmVQ* genes under cold treatment compared to the controls. Stress treatments and time course are described in the Materials and Methods section. (A-Y) represent different genes which were used in qRT-PCR analysis. Asterisks on top of the bars indicating statistically significant differences between the stress and counterpart controls (*p < 0.05, **p < 0.01).

**Figure 9:**
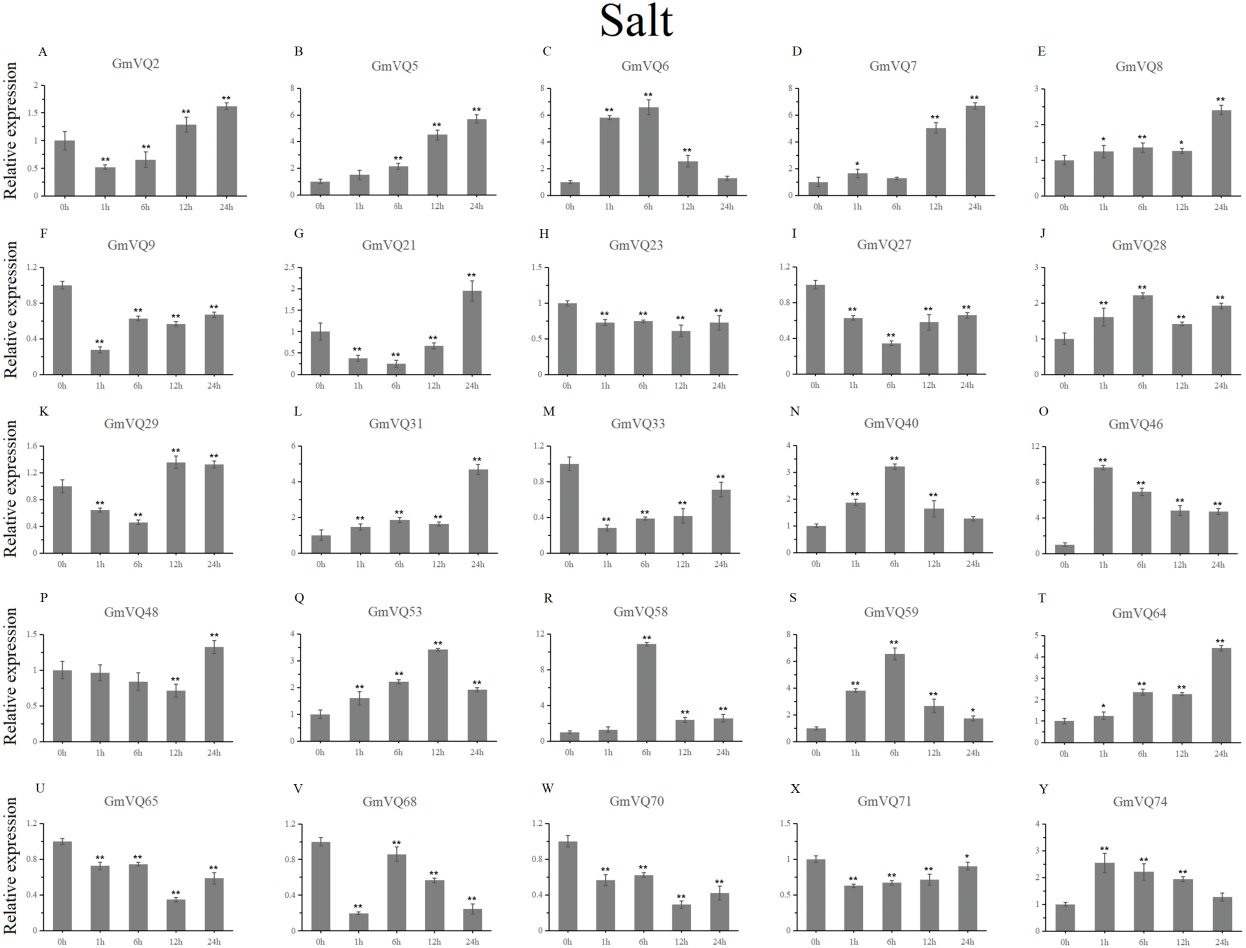
qRT-PCR analysis reveals *GmVQ* genes under NaCl treatment compared to the controls. Stress treatments and time course are described in the Materials and Methods section. (A-Y) represent different genes which were used in qRT-PCR analysis. Asterisks on top of the bars indicating statistically significant differences between the stress and counterpart controls (*p < 0.05, **p < 0.01).

**Figure 10:**
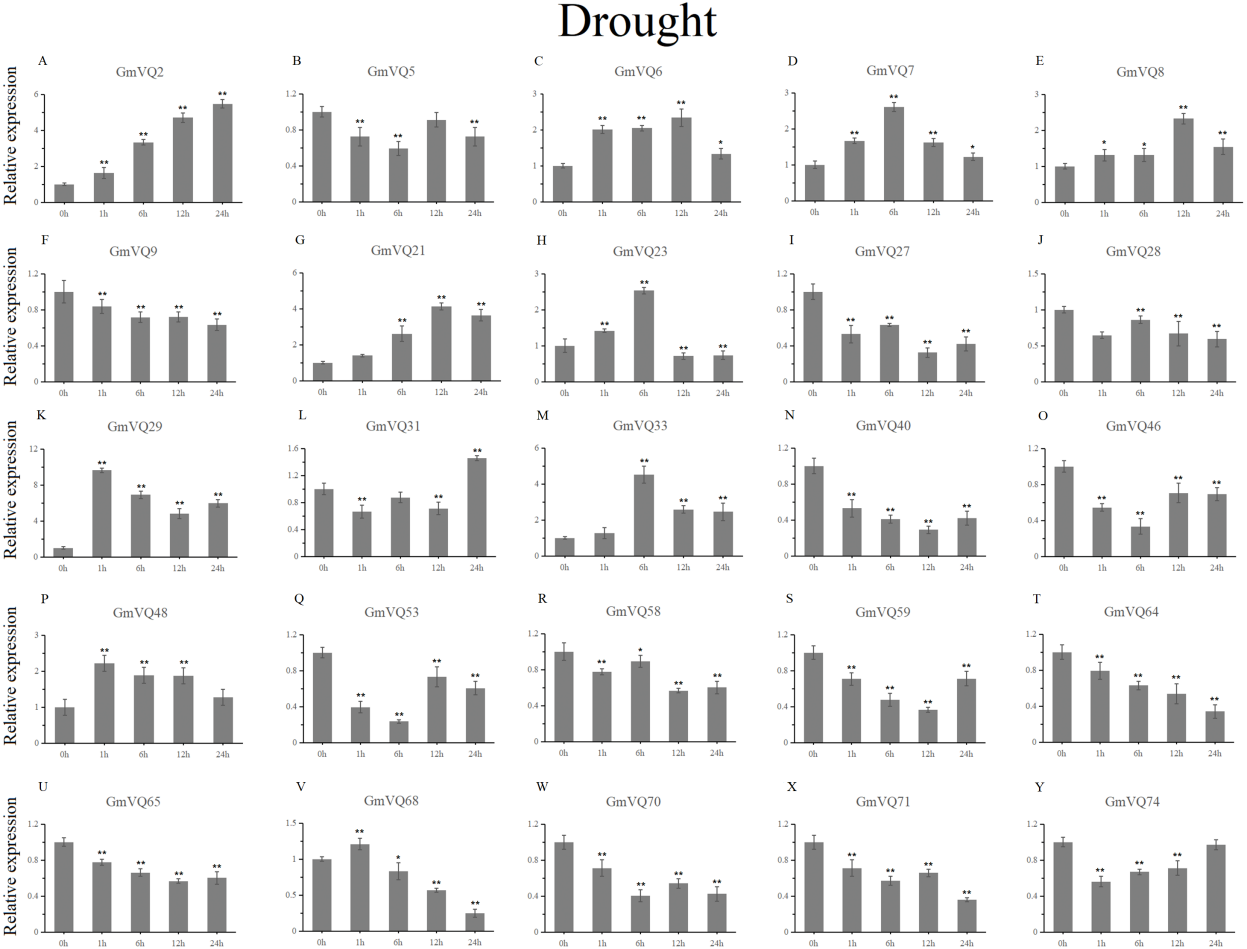
qRT-PCR analysis reveals *GmVQ* genes under drought treatment compared to the controls. Stress treatments and time course are described in the Materials and Methods section. (A-Y) represent different genes which were used in qRT-PCR analysis. Asterisks on top of the bars indicating statistically significant differences between the stress and counterpart controls (*p < 0.05, **p < 0.01).

We randomly selected 25 *GmVQ* genes from seven groups, and made sure their responses to the plant hormones-, cold-, salt-, and drought-stress (Figs. 6–10). Under ABA treatment, most genes were up-regulated whole treatment period and six genes (*GmVQ6/8/31/33/59/71*) were obviously down-regulated at some treatment time points (Fig. 6, Table S3). The expression levels of seven genes (*GmVQ2/27/40/48/53/68/74*) reached the peak at the 6 h treatment time point and four genes (*GmVQ9/21/31/71*) reached the lowest expression levels at the early treatment time points (0–1 h treatment). With SA treatment, the expression levels of most *GmVQs* were down-regulated throughout, while *GmVQ7* was up-regulated at 1 h, 6 h and 12 h treatment time points (Fig. 7, Table S4). In addition, nine *GmVQ* genes (*GmVQ5/6/8/23/31/68/70/71/74*) were down-regulated under all abiotic stress.

With cold treatment, the expression levels of fourteen *GmVQ* genes (*GmVQ2/7/9/28/29/31/33/40/46/48/53/59/68/74*) were up-regulated throughout (Fig. 8, Table S5), while the expression levels of three genes (*GmVQ27/64/65*) were down-regulated and then up-regulated during treatment. Under salt stress, the results were similar to that with cold stress treatment, most genes were up-regulated, eight genes (*GmVQ9/23/27/33/65/68/70/71*) were down-regulated throughout (Fig. 9, Table S6). On the contrary, under drought (PEG) stress, most genes were down-regulated, only eight genes (*GmVQ2/6/7/8/21/29/33/48*) were up-regulated during the treatment (Fig. 10, Table S7).

### *Cis*-elements in *GmVQ* promoters

**Figure 11:**
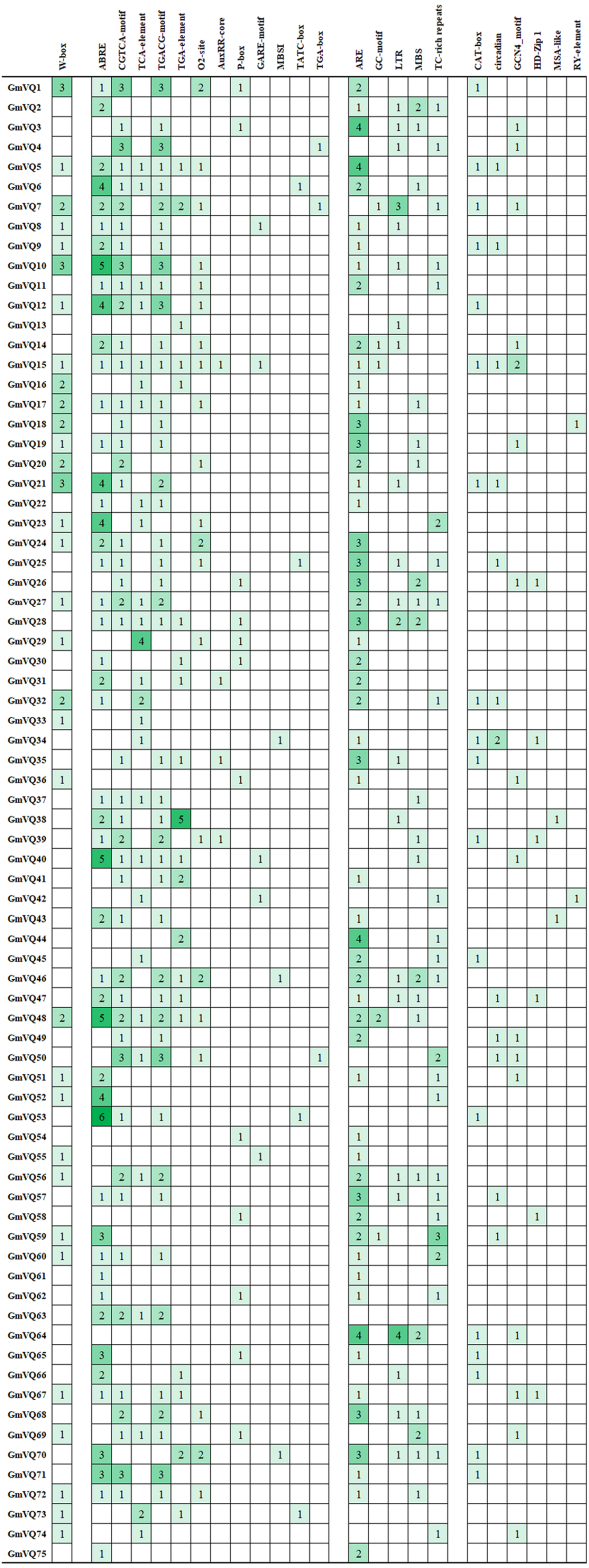
Number of each cis-acting element in the promoter region (1.5 kb upstream of the translation start site) of *GmVQ* genes.

We found many hormone- and stress- related promoter’s *cis*-elements in *GmVQ* genes. Enhancer regions (CAAT-box) and core promoter element are around −30 bp of transcription start (TATA-box). *Cis*-acting regulatory element (A-box) are the common *cis*-acting elements in the promoter. Others *cis*-elements that were found in the 75 *GmVQ* s can be classified into three groups (Fig. 11). Twelve *cis*-elements involve in the hormone responsiveness; five *cis*-elements are stress-related elements: ARE/GC/LTR/MBS/TC; some *GmVQ* genes containe plant growth and development elements, such as CAT-box/circadian/GCN4/HD-Zip 1/MSA-like/RY-element. In addition, some *GmVQ* genes containe W-box motif, which is binding site for WRKY transcription factor.

### Gene Ontology Enrichment

To further understand the functions of the *GmVQs*, we performed GO annotation and GO enrichment analyses (Fig. S2 and Table S8). The GO terms included three categories, biological process (BP), molecular function (MF) and cellular component (CC). GO enrichment confirmed that these *GmVQs* were enriched in the biological process (GO:0008150), regulation of biological process (GO:0050789) and biological regulation (GO:0065007) terms of the BP category. Cellular component (GO:0005575), intracellular (GO:0005622) and cell (GO:0005623) were the most abundant functions in the CC category (Table S8). MF was enriched in molecular function (GO:0003674) and binding (GO:0005488). The GO ebrichment suggested that *GmVQs* were play curcial roles in regulated of biological process.

### Gene interaction network analysis

**Figure 12:**
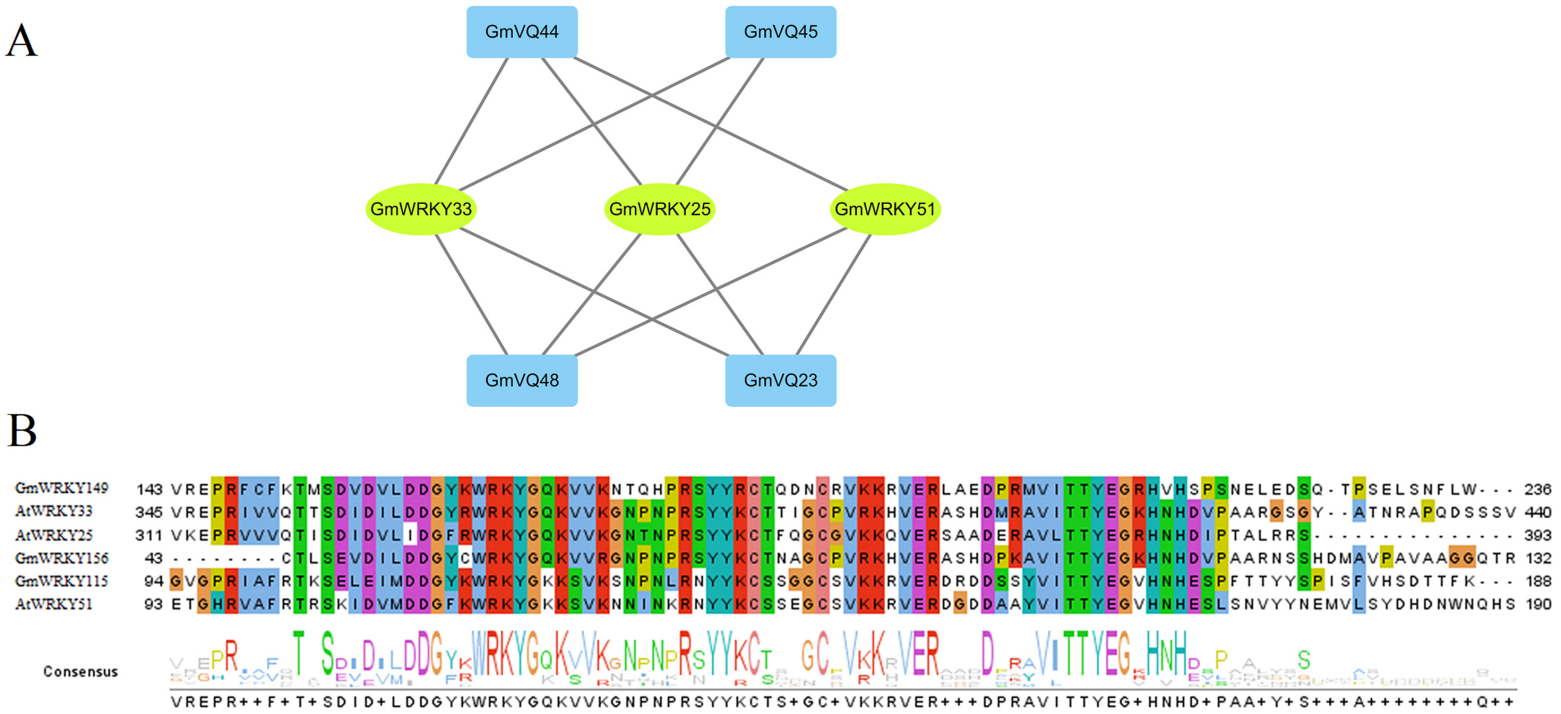
Interaction of GmVQ proteins with GmWRKY proteins. (A) The prediction of interaction between GmVQ proteins and GmWRKY proteins by the PAIR website, and the interaction network was draw in Cytoscape 3.6.1. (B) Sequence analysis of the WRKY domains of GmWRKY proteins and AtWRKY proteins.

Based on the PAIR tool, we found the functions and their interactions of the GmVQs and GmWRKYs. As shown in Fig. 12A, 3 GmWRKYs are supposed to interact with GmVQ proteins, included GmWRKY115, GmWRKY149 and GmWRKY156, all of them belong to WRKY’s IIc group. In the Fig. 12B, we found that GmWRKYs and AtWRKYs are quite similar in their core domains, indicated that they might have same functions, such as interacted with VQ proteins.

## Discussion

VQ protein is a kind of specific protein that widely exists in plant, involved in plant growth and can response to different stresses (Petersen et al., 2010; Fiil & Petersen, 2011; Xie et al., 2010). Hence, we completed genome-wide analysis of soybean VQ proteins by bioinformatic analysis and qRT-PCR to understand their regulation when environmental changed. In the previous study, 74 *GmVQ* genes were identified (Wang et al., 2014; Zhou et al., 2016). After the database was updated, we identified and isolated 75 *GmVQ* genes in the soybean genome. Compared with previous study, the number of genes in chromosome 2, 4 and 17 show a big difference. Soybean contains more VQ genes than that of *A. thaliana* (34) (Cheng et al., 2012), *Populus trichocarpa* (51) (Chu et al., 2016) and *O. sativa* (42) (Kim et al., 2013a; Kim et al., 2013b). The reason is the whole genome duplication events (WGD). There are two rounds of genome duplication, occurred at around 59 and 13 million years ago, which caused 75% soybean genes duplicated (Jeremy et al., 2010).

Seventy-five *VQ* genes were identified in *Glycine max’s* genome, divided into seven groups based on their comprehensive phylogenetic tree among *G. max*, *A. thaliana*, and *O. sativa*. These proteins are in the shorter branches and with closer spacing, suggesting that they were highly conserved during the evolution. The more closer related genes within the same group shared more similar gene structures, either in their intron or in the exon patterns. Whereas, the variation in different groups suggested the functional diversity of the *VQ* genes (Jiang, Sevugan & Ramachandran, 2018). In addition, most *GmVQ* genes (59; 78.67%) were found intronless, and most *GmVQ* genes (64; 85.33%) encoded relatively small proteins with protein length less than 300 amino acid. This suggests that *VQ* gene families were intronless and they were highly conserved during evolution. At the same time, gene duplication can help plants to adapt to different environments during their development and growth (Huang et al., 2016; Storz, 2009). The main expansion of *GmVQ* gene family is segmental duplication (52; 92.9%), only 4 pairs of genes involved in tandem duplication events (4; 7.1%). A similar phenomenon was reported in the *BrVQ* gene family, which contains a high proportion of segmental duplication (71.9%) and low proportion of tandem duplication (28.1%) (Zhang et al., 2015).

Nonfunctionalization, subfunctionalization, and neofunctionalization generally take place after genome duplication, resulting in lose or fix of genes (He & Zhang, 2005; Sandve, Rohlfs & Hvidsten, 2018; Stark et al., 2017). Soybean has undergone the WGD and the whole genome triplication (WGT) compared to grapevine (Wang et al., 2017). As there are 18 *VQ* genes in grapevine genome, the predicted number of VQ genes in soybean should be more than 100 (Wang et al., 2015). However, in this study, we only found 75 *VQ* genes in the soybean genome, suggesting that there were gene loss events after genome duplication. In addition, the Ks value of each paralogous pairs was calculated to find gene duplication events, the most duplication events in *GmVQ* gene occurred between 5 and 30 MYA, consistent with the recent WGD in soybean (Wang et al., 2017; Jeremy et al., 2010). The *Ka/Ks* ratios in different gene pairs are different, but most gene pairs’ *Ka/Ks* ratios are less than one and only two gene pairs’ (*GmVQ54*-*GmVQ63* and *GmVQ65*-*GmVQ36*) ratios are larger than 1, implying these gene pairs undergo different selection pressure. The above analysis indicated that purifying selection played a crucial role during the evolution, conserved *VQ* proteins evolved much slowly at the protein level.

Expression patterns of 67 *GmVQ* genes were performed to determine their tissue expression using RNA-seq data. The results showed that 24 genes were relatively highly expressed in nine tissues, indicated that they may relate to the growth and development of plants. Moreover, 76% (57/75) and 64% (48/75) of *GmVQ* genes’ expression levels were obviously increased in leaves and roots, respectively. More and more studies have shown that VQ proteins played a significant role in plants development. The study of *A. thaliana* mutants showed that *AtVQ8* had a certain influence on chlorophyll formation and leaf growth and development (Cheng et al., 2012). In this study, *GmVQ7* and *GmVQ75* were in the same evolutionary branch with *AtVQ8*. Their high expression in leaves indicating they might have similar function as *AtVQ8* (Cheng et al., 2012). These results will help us to study the further function of soybean’s VQ proteins.

Plants need to face various abiotic stresses during their growth in natural conditions, the most common of which are high salt, drought and cold (Kim et al., 2013a; Kim et al., 2013b; Wang et al., 2014). Except for regulation by environmental factors, *VQ* gene family is regulated by defense-related hormones, such as SA and ABA. In our study, we selected 25 *GmVQs* for qRT-PCR analysis under five different stresses (salt, drought, cold, SA and ABA stresses). In this study, most *GmVQ* genes were up-regulated with the SA treatment, the result is consistent with previous study that most *AtVQ* genes can response to pathogen or the SA treatment (Cheng et al., 2012). In addition, fifteen *GmVQ* genes (e.g., *GmVQ2/21/29/46*) were up-regulated under SA treatment, suggesting that they play a potential role in stress resitance. 56% *GmVQ* genes (14/25) were up-regulated, which is different with the up-regulation of *VQ* genes in rice that only three *OsVQ* genes were up-regulated more than two fold (Kim et al., 2013a; Kim et al., 2013b). Increasing evidence suggests that *VQ* genes are involved in various stress response. For example, 23% *ZmVQ* genes were upregulated, all the *VvVQ* genes were up-regulated by drought stress (Song et al., 2016; Wang et al., 2015). Consistently, 30% of *GmVQ* genes were up-regulated, *GmVQ2/29/33* were highly expressed under drought stress. Nevertheless, *AtVQ9* and *AtVQ15* were reported can response to abiotic stress during high salinity treatment. The response of *VQ* genes to cold stress is similar to that of Chinese cabbage (Hu et al., 2013; Zhang et al., 2015; Cheng et al., 2012). In our study, *GmVQ5/6/7/31/46/58/59* and *GmVQ7/9/28* were activated the salt and cold stresses, respectively, because that their promoter region exists in specific stress *cis*-elements. Besides, homologous *GmVQ* genes possessed similar expression pattern but may exhibit opposite expression trend under stress, such as *GmVQ9*-*GmVQ21* were** up-regulated under SA treatment, but *GmVQ9* was up-regulated and *GmVQ29* was down-regulated during cold stress. These results suggest that *GmVQ* genes participate in response mechanism of abiotic stresses, their regulation mechanism is complex and diverse.

As auxiliary factor, VQ genes regulate transcription, can interact with many proteins to participate in regulating complex physiological and biochemical processes of plants, such as they can interact with *WRKY* transcription factors (Wang et al., 2015; Lei et al., 2017; Lai et al., 2011). Studies have shown that the responses under three different pathogens, *VQ* protein are interacted with *WRKY* protein in rice (Li et al., 2014). VQ proteins and WRKY proteins may form a protein complex to exercise function. We found some of the *GmVQ* genes interact with group I’s *WRKY* , most *VQ* genes interact with groups I and IIc’s VQ protein in various stresses in previous reports (Dong et al., 2018; Guo et al., 2018; Lei et al., 2017). The promoter analysis indicated that 23 of 75 *GmVQ* genes (30.67%) contained one or more W-box motif in their 1,500 bp promoter regions, W-box were present in 78% *VvVQ* genes, 91% *ZmVQ* genes contained one or more W-box motif (Song et al., 2016). In the promoters of *GmWRKY* genes, W-boxes could regulate *GmWRKY* members (Dong, Chen & Chen, 2003). It indicates that WRKY protein affect VQ genes expression and thus responses to environmental stimuli (Dong et al., 2018; Guo et al., 2018).

## Conclusions

Seventy-five *VQ* genes were identified in the soybean genomes. All *VQ* genes fell into seven groups (I-VII). *VQ* genes from the same evolutionary branches of soybean shared similar motifs and structures. The selection pressure analysis showed that most of the paralogous pairs were under a strong purifying selection in the *GmVQ* genes. RNA-seq analysis revealed that the *VQ* genes had different expression patterns in different tissues, indicating that they play crucial roles in different tissue. Finally, qRT-PCR showed that the *VQ* gene family was responsive to biotic and abiotic stresses. Our results provide a theoretical basis for further study on the function of *GmVQs*.

##  Supplemental Information

10.7717/peerj.7509/supp-1 Figure S1 Sequence logo of motifs in *GmVQ* genes

10.7717/peerj.7509/supp-2 Figure S2 Gene ontology categories assigned of the *GmVQ* genes

10.7717/peerj.7509/supp-3 Table S1 List of primers used in qRT-PCR

10.7717/peerj.7509/supp-4 Table S2 List of VQ gene duplication events

10.7717/peerj.7509/supp-5 Table S3 Raw data for the ABA stress

10.7717/peerj.7509/supp-6 Table S4 Raw data for the SA stress

10.7717/peerj.7509/supp-7 Table S5 Raw data for the salt stress

10.7717/peerj.7509/supp-8 Table S6 Raw data for the drought stress

10.7717/peerj.7509/supp-9 Table S7 Raw data for the cold stress

10.7717/peerj.7509/supp-10 Table S8 GO terms of the *GmVQ* genes

## Additional Information and Declarations

### Data Availability

The following information was supplied regarding data availability: The raw measurements are available in Fig. S1 and Table S1.
