# Supplementary figures and images for: Genome-wide identification and expression analysis of the VQ gene family in soybean (Glycine max)
Source: PeerJ. 2019 Aug 21;7:e7509. doi: 10.7717/peerj.7509 (PMC6708371; doi:10.7717/peerj.7509)

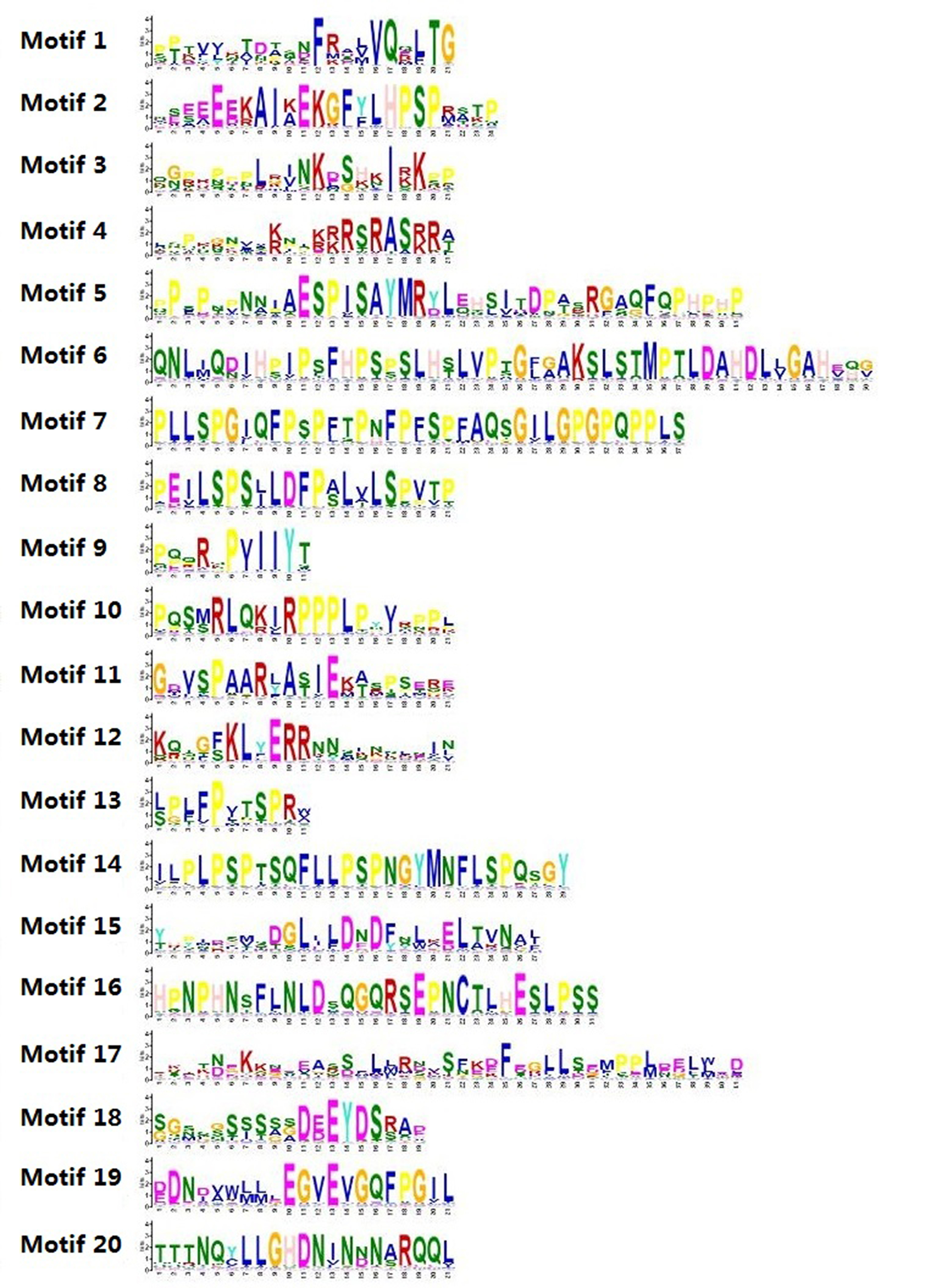

Supplement: Figure S1 [file peerj-07-7509-s001.png]

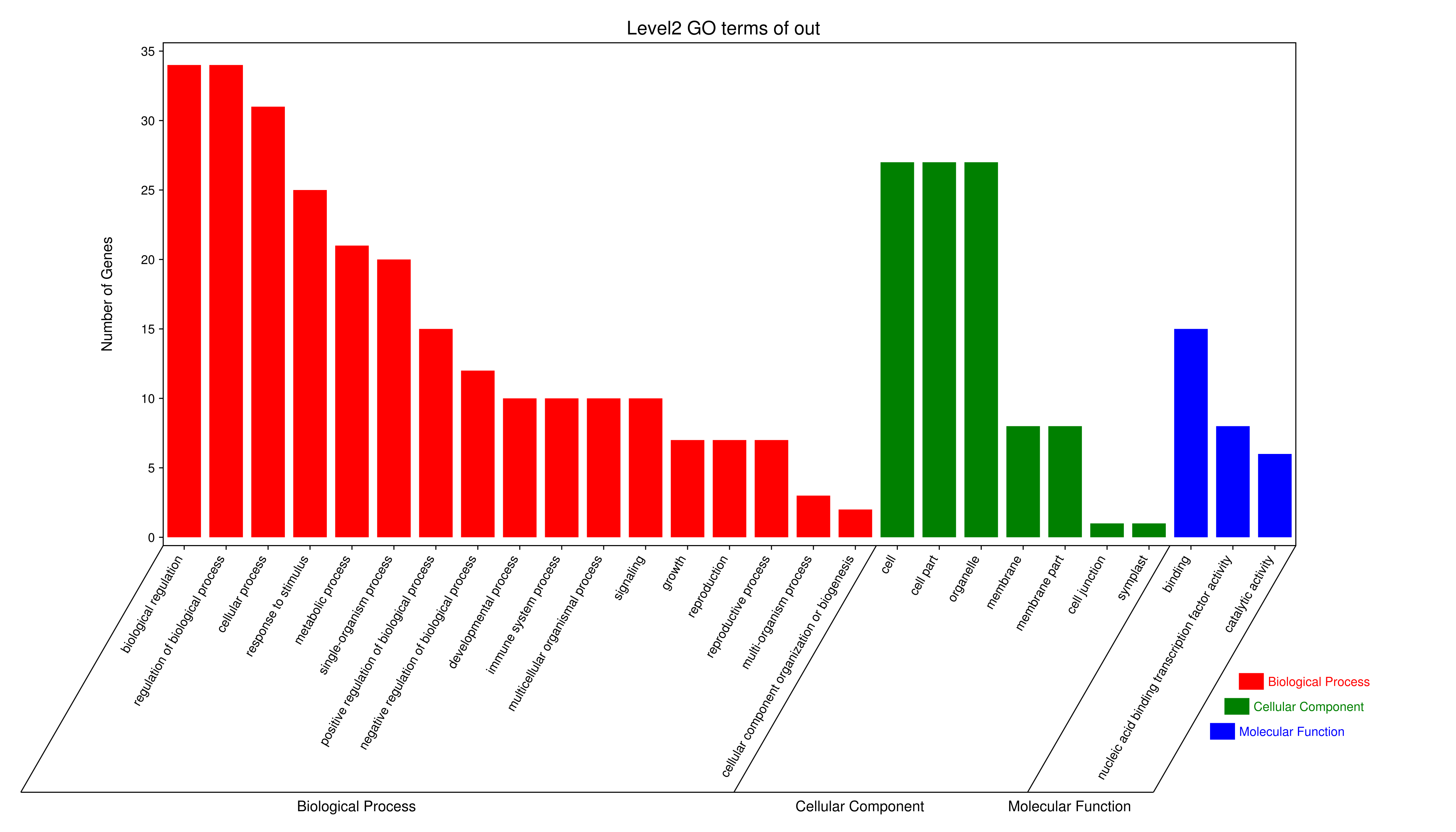

Supplement: Figure S2 [file peerj-07-7509-s002.png]
