# Supplementary material for: Genome-wide identification and expression analysis of the VQ gene family in soybean (Glycine max)
Source: PeerJ. 2019 Aug 21;7:e7509. doi: 10.7717/peerj.7509 (PMC6708371; doi:10.7717/peerj.7509)
Supplement: Table S1 [file peerj-07-7509-s003.docx]

| **Table S1 List of primers used in qRT-PCR** | | |
| --- | --- | --- |
| Gene name | Forward primer (5'-3') | Reverse primer(5'-3') |
| GmVQ2 | GGGGTGCCCAATTTCAACAC | GGGAAGGAACATCAGGCACA |
| GmVQ5 | TCAGAAGTCTCTCTCCGGTA | GGGACTTCCTCTTGGAGAT |
| GmVQ6 | AATTCACCCTGATTCCTCCT | GAGGCTCATATTCTCGTCG |
| GmVQ7 | ATAAGAAGCAGAAGCAGCAC | TGCAACCAGTAAAATCCTCT |
| GmVQ8 | CAAATTGCTACCACCACAAG | TCTTCATCCAACTCCAGAGA |
| GmVQ9 | CGGTGTACAACATCAACAAG | GAAGTTGTTGAAGGTAACGG |
| GmVQ21 | CATTAAGAAACCCGTCTTTG | CGGTTAGTAATTTGAGGCAG |
| GmVQ23 | AACAACCACCCCTGACTCTC | GCCTTGAAGCTCTGCTTTCG |
| GmVQ27 | AACACCACCTTCGTCCAA | TTGAAGTTCGGCTTCTTG |
| GmVQ28 | ACAACCCTCAAACCAGAAAT | TCTCCCACGCTACTACAATA |
| GmVQ29 | CCGAAGCTACTGAAGAGAAA | AATTGAAGGGTTTGACAGTG |
| GmVQ31 | AATCATCATCTTCATCCCTG | GTTTTAAGACCTCTTCGCCT |
| GmVQ33 | CAAAGATAGTGCACATCGAA | ATGAGTAGCATGGAAGATGC |
| GmVQ40 | AAAAACATTAGGAAGAGCCC | GGATGATCCTGAGACTGAGA |
| GmVQ46 | GATACGCAAAGAAACCAAAC | ATTGTAGTTGGACCCATCAG |
| GmVQ48 | CGGATCAGCGAGTCAAGGTT | CGTCGAAACGAAAACCCTCC |
| GmVQ53 | CCTACCACCGTGTCTGTTCC | GAGTTAGGTTTGGGGGAGGC |
| GmVQ58 | AGTTCACTGGAGCTCCTTCT | CGATCATTATTTGGAGCTGT |
| GmVQ59 | GATGATGTTGTTGGAGGGAT | AAGAACCGGGCTTAAATCAT |
| GmVQ64 | AATACCCAATACGTCGAAAC | CTGAGCAACCTATCGAACTC |
| GmVQ65 | AGGCTCACAGGAAAGCCAAA | CCATGACCTTCTTGGGGTGT |
| GmVQ68 | TGGATCCGACACAAAGACCG | GAGGGAGAGTGGTACTGGGA |
| GmVQ70 | CCTGTCACCGATTCATGGCT | CGCCGGAGAGAGACTTCTGA |
| GmVQ71 | GAGGAGGAGGAAGAGGATAA | CAGTGATTAAGATGAGGGGA |
| GmVQ74 | TCACCCCCTATATCCTACCT | ACATTGCTTGTTGGAAGACT |
