# Supplementary material for: Genome-wide identification and expression analysis of the VQ gene family in soybean (Glycine max)
Source: PeerJ. 2019 Aug 21;7:e7509. doi: 10.7717/peerj.7509 (PMC6708371; doi:10.7717/peerj.7509)
Supplement: Table S2 [file peerj-07-7509-s004.docx]

| Table S2 List of VQ gene duplication events | | |
| --- | --- | --- |
| Segmental duplication | | Tandem duplication |
| GmVQ3-GmVQ47 | GmVQ24-GmVQ59 | GmVQ10-GmVQ11 |
| GmVQ5-GmVQ70 | GmVQ27-GmVQ38 | GmVQ22-GmVQ23 |
| GmVQ6-GmVQ71 | GmVQ28-GmVQ45 | GmVQ39-GmVQ40 |
| GmVQ7-GmVQ72 | GmVQ29-GmVQ53 | GmVQ40-GmVQ41 |
| GmVQ8-GmVQ73 | GmVQ29-GmVQ61 |  |
| GmVQ9-GmVQ21 | GmVQ29-GmVQ56 |  |
| GmVQ10-GmVQ22 | GmVQ34-GmVQ68 |  |
| GmVQ10-GmVQ23 | GmVQ34-GmVQ48 |  |
| GmVQ10-GmVQ67 | GmVQ37-GmVQ64 |  |
| GmVQ11-GmVQ22 | GmVQ39-GmVQ41 |  |
| GmVQ11-GmVQ23 | GmVQ39-GmVQ2 |  |
| GmVQ11-GmVQ67 | GmVQ40-GmVQ2 |  |
| GmVQ13-GmVQ25 | GmVQ41-GmVQ2 |  |
| GmVQ14-GmVQ24 | GmVQ42-GmVQ69 |  |
| GmVQ14-GmVQ52 | GmVQ43-GmVQ62 |  |
| GmVQ14-GmVQ59 | GmVQ46-GmVQ75 |  |
| GmVQ15-GmVQ66 | GmVQ49-GmVQ26 |  |
| GmVQ16-GmVQ32 | GmVQ50-GmVQ57 |  |
| GmVQ18-GmVQ34 | GmVQ51-GmVQ74 |  |
| GmVQ18-GmVQ68 | GmVQ52-GmVQ59 |  |
| GmVQ18-GmVQ48 | GmVQ53-GmVQ61 |  |
| GmVQ19-GmVQ35 | GmVQ53-GmVQ56 |  |
| GmVQ20-GmVQ30 | GmVQ54-GmVQ63 |  |
| GmVQ22-GmVQ67 | GmVQ61-GmVQ56 |  |
| GmVQ23-GmVQ67 | GmVQ65-GmVQ36 |  |
| GmVQ24-GmVQ52 | GmVQ68-GmVQ48 |  |
