# Supplementary material for: Genome-wide identification and expression analysis of the VQ gene family in soybean (Glycine max)
Source: PeerJ. 2019 Aug 21;7:e7509. doi: 10.7717/peerj.7509 (PMC6708371; doi:10.7717/peerj.7509)
Supplement: Table S3 [file peerj-07-7509-s005.docx]

| Table S3 Raw data for the ABA stress | | | | | | | | | | | | | | | |
| --- | --- | --- | --- | --- | --- | --- | --- | --- | --- | --- | --- | --- | --- | --- | --- |
| Gene | 0h | | | 1h | | | 6h | | | 12h | | | 24h | | |
| actin | 24.7424 | 25.4777 | 25.8974 | 24.7255 | 24.7774 | 24.6982 | 26.0245 | 25.7375 | 26.9825 | 24.8577 | 25.1777 | 24.7789 | 24.7734 | 25.1775 | 24.3784 |
| GmVQ2 | 28.6142 | 28.6579 | 28.7018 | 27.4138 | 27.2277 | 27.3298 | 28.3401 | 28.4024 | 28.3087 | 27.7102 | 27.5812 | 27.5317 | 26.9141 | 26.8413 | 26.7907 |
| GmVQ5 | 30.5294 | 30.7173 | 30.6399 | 29.5269 | 29.6220 | 29.4728 | 30.8741 | 30.9942 | 30.9185 | 29.3307 | 29.3798 | 29.4425 | 29.1491 | 29.1460 | 29.2638 |
| GmVQ6 | 28.6690 | 28.7187 | 28.6938 | 28.9136 | 29.0388 | 28.7407 | 29.8319 | 29.9420 | 29.8968 | 28.6250 | 28.7248 | 28.6863 | 27.9962 | 28.0498 | 28.0338 |
| GmVQ7 | 32.4308 | 32.4528 | 32.4197 | 30.3396 | 30.3646 | 30.4196 | 32.1048 | 31.9712 | 31.9271 | 31.3931 | 31.4982 | 31.0607 | 31.3804 | 31.7153 | 31.4678 |
| GmVQ8 | 32.7269 | 32.7766 | 32.8766 | 32.5215 | 32.4039 | 32.5579 | 34.2791 | 34.3925 | 34.3547 | 32.7246 | 32.8244 | 32.7859 | 32.5643 | 32.4467 | 32.6006 |
| GmVQ9 | 28.7495 | 28.7473 | 28.5860 | 29.8203 | 29.4370 | 30.0905 | 30.9315 | 31.3045 | 30.7470 | 29.2538 | 29.5609 | 29.4754 | 28.4638 | 28.5636 | 28.5252 |
| GmVQ21 | 30.6351 | 30.6435 | 30.6258 | 30.8792 | 31.0834 | 30.8683 | 31.9610 | 31.8273 | 31.9740 | 29.8834 | 29.6990 | 30.0696 | 29.1416 | 29.2955 | 29.3416 |
| GmVQ23 | 28.6568 | 28.6388 | 28.7396 | 27.8793 | 27.7307 | 27.5244 | 28.9865 | 28.9701 | 28.8579 | 27.0129 | 27.1361 | 26.9769 | 26.3693 | 26.2658 | 26.4964 |
| GmVQ27 | 31.3244 | 31.4868 | 31.4760 | 29.0468 | 28.8328 | 29.2068 | 29.2493 | 29.0607 | 29.0737 | 28.2487 | 28.4149 | 28.2638 | 28.7010 | 28.6154 | 28.7413 |
| GmVQ28 | 32.0896 | 32.0717 | 32.1724 | 31.9604 | 32.1367 | 32.1482 | 34.1594 | 34.2692 | 34.3157 | 33.6149 | 33.2550 | 33.6006 | 32.5649 | 33.0720 | 32.7194 |
| GmVQ29 | 28.6541 | 28.8151 | 28.7542 | 28.0342 | 27.8921 | 27.9887 | 29.8792 | 30.0855 | 30.2408 | 28.6028 | 28.6780 | 28.7339 | 28.3793 | 28.6178 | 28.2991 |
| GmVQ31 | 28.7118 | 28.7185 | 28.6424 | 29.8376 | 30.1302 | 29.8041 | 29.9784 | 30.0768 | 30.0443 | 29.1119 | 29.1085 | 29.0104 | 29.8003 | 30.3622 | 30.2997 |
| GmVQ33 | 28.7244 | 28.6601 | 28.5888 | 28.3222 | 28.3971 | 28.4534 | 29.9504 | 30.0048 | 30.2073 | 28.4649 | 28.3500 | 28.4023 | 29.9857 | 29.4920 | 29.6585 |
| GmVQ40 | 33.6778 | 33.7411 | 33.5887 | 32.3757 | 32.1665 | 32.3318 | 33.7402 | 33.7794 | 33.6231 | 32.8140 | 32.7279 | 32.7288 | 32.3403 | 32.3863 | 32.4760 |
| GmVQ46 | 33.6558 | 33.8182 | 33.8074 | 34.0029 | 34.4461 | 33.8301 | 35.8640 | 35.7407 | 35.9916 | 33.9449 | 34.2799 | 34.2288 | 34.4239 | 34.0929 | 33.8190 |
| GmVQ48 | 28.7262 | 28.7329 | 28.6568 | 27.3946 | 27.1853 | 27.3507 | 27.4584 | 27.3467 | 27.4419 | 26.5290 | 26.4567 | 26.4942 | 27.3591 | 27.3910 | 27.4783 |
| GmVQ53 | 28.6648 | 28.6731 | 28.6554 | 26.8959 | 27.3075 | 26.9160 | 27.6728 | 27.6664 | 27.7901 | 26.5446 | 26.6409 | 26.9562 | 27.5982 | 27.4852 | 27.6856 |
| GmVQ58 | 28.6546 | 28.5223 | 28.7323 | 24.7581 | 24.6866 | 24.7439 | 26.7762 | 26.7728 | 26.6302 | 25.8950 | 26.1237 | 25.7886 | 25.6151 | 25.7223 | 25.5192 |
| GmVQ59 | 33.6067 | 33.7677 | 33.7068 | 33.4219 | 33.3043 | 33.4582 | 35.1795 | 35.2928 | 35.2551 | 34.6684 | 34.8886 | 34.8015 | 33.6638 | 33.7386 | 33.7056 |
| GmVQ64 | 33.8766 | 33.7070 | 33.7660 | 32.6695 | 32.5192 | 32.1622 | 32.7055 | 32.9905 | 32.9190 | 30.9001 | 31.0296 | 31.1282 | 30.4073 | 30.5044 | 30.4358 |
| GmVQ65 | 32.0736 | 32.1206 | 32.1625 | 31.0051 | 31.0837 | 31.1254 | 31.9220 | 31.9633 | 32.0440 | 29.7109 | 30.0038 | 29.9579 | 29.4026 | 29.2126 | 29.2856 |
| GmVQ68 | 28.6910 | 28.7292 | 28.7799 | 26.0334 | 25.8849 | 25.8198 | 27.3961 | 27.3911 | 27.4889 | 27.1512 | 26.7125 | 27.0278 | 27.6698 | 28.0047 | 27.7572 |
| GmVQ70 | 28.5264 | 28.6751 | 28.8930 | 27.8837 | 27.7351 | 27.5288 | 28.9909 | 29.1786 | 29.3879 | 27.8068 | 28.0268 | 27.8633 | 26.8919 | 26.7458 | 27.0737 |
| GmVQ71 | 28.4792 | 28.6589 | 28.8569 | 29.8044 | 30.0971 | 29.7710 | 29.9453 | 30.2641 | 30.0112 | 29.0788 | 29.0754 | 28.9773 | 28.3313 | 28.4415 | 28.3963 |
| GmVQ74 | 33.5093 | 33.6859 | 33.8859 | 32.0978 | 31.8959 | 32.0982 | 32.7107 | 32.8447 | 32.9361 | 32.7845 | 32.6341 | 32.2771 | 33.2942 | 32.8607 | 32.6755 |
