# Supplementary material for: Genome-wide identification and expression analysis of the VQ gene family in soybean (Glycine max)
Source: PeerJ. 2019 Aug 21;7:e7509. doi: 10.7717/peerj.7509 (PMC6708371; doi:10.7717/peerj.7509)
Supplement: Table S4 [file peerj-07-7509-s006.docx]

| Table S4 Raw data for the SA stress | | | | | | | | | | | | | | | |
| --- | --- | --- | --- | --- | --- | --- | --- | --- | --- | --- | --- | --- | --- | --- | --- |
| Gene | 0h | | | 1h | | | 6h | | | 12h | | | 24h | | |
| actin | 24.7424 | 25.4777 | 25.8974 | 24.7255 | 24.7774 | 24.6982 | 26.0245 | 25.7375 | 26.9825 | 24.8577 | 25.1777 | 24.7789 | 24.7734 | 25.1775 | 24.3784 |
| GmVQ2 | 28.4939 | 28.6426 | 28.8605 | 27.6598 | 27.4017 | 27.0447 | 27.8309 | 27.7455 | 27.5448 | 25.7748 | 25.9122 | 26.0108 | 25.2905 | 25.3870 | 25.3184 |
| GmVQ5 | 30.4541 | 30.6338 | 30.8318 | 29.9927 | 30.3336 | 30.2912 | 31.9022 | 31.7058 | 31.6874 | 30.5010 | 30.4009 | 30.4506 | 31.0944 | 31.2786 | 31.2614 |
| GmVQ6 | 28.5094 | 28.6860 | 28.8860 | 29.4404 | 29.6769 | 29.6545 | 30.5657 | 30.5112 | 30.3094 | 29.9192 | 29.6824 | 29.8489 | 30.4820 | 30.9707 | 30.4314 |
| GmVQ7 | 32.3413 | 32.5292 | 32.4518 | 31.8868 | 31.5254 | 31.6771 | 32.9837 | 32.6051 | 32.6252 | 31.8570 | 31.6493 | 31.9188 | 32.9063 | 32.1334 | 33.4552 |
| GmVQ8 | 32.7685 | 32.8182 | 32.7934 | 32.9784 | 32.7185 | 32.9213 | 35.4473 | 35.1227 | 34.9968 | 32.8717 | 32.5061 | 32.5867 | 33.1912 | 33.4983 | 33.7511 |
| GmVQ9 | 28.6278 | 28.6775 | 28.7775 | 24.6708 | 24.7889 | 24.8018 | 26.1106 | 26.1234 | 26.2698 | 25.5936 | 25.6584 | 25.5322 | 26.9427 | 26.5040 | 26.8193 |
| GmVQ21 | 30.6190 | 30.6409 | 30.6220 | 28.8417 | 28.6449 | 28.9728 | 31.3138 | 31.2280 | 31.3797 | 29.8377 | 29.4941 | 30.0182 | 29.8682 | 30.1148 | 29.7283 |
| GmVQ23 | 28.7490 | 28.7468 | 28.5855 | 28.8340 | 28.9848 | 28.9709 | 31.3001 | 31.2106 | 30.8960 | 30.6437 | 31.0600 | 30.5927 | 29.7575 | 29.5207 | 29.6873 |
| GmVQ27 | 31.3099 | 31.6077 | 31.4376 | 30.5411 | 30.5222 | 30.4936 | 30.6059 | 30.6336 | 30.6778 | 28.7103 | 28.9390 | 28.6039 | 28.1841 | 28.0511 | 28.1029 |
| GmVQ28 | 32.0808 | 32.1618 | 32.1363 | 31.1501 | 31.0673 | 31.1471 | 32.2220 | 32.3020 | 32.2310 | 30.3772 | 30.4692 | 30.4062 | 29.7521 | 29.8376 | 30.0030 |
| GmVQ29 | 28.7041 | 28.8322 | 28.9441 | 27.7546 | 27.8654 | 27.7921 | 27.1603 | 27.2977 | 27.3963 | 25.7547 | 25.6241 | 25.5555 | 28.1031 | 27.9560 | 27.4480 |
| GmVQ31 | 28.6111 | 28.7721 | 28.7112 | 28.2513 | 28.3087 | 28.2834 | 30.9850 | 31.6635 | 31.1891 | 29.1119 | 29.1320 | 29.0104 | 28.3645 | 28.2991 | 28.4294 |
| GmVQ33 | 28.6787 | 28.8397 | 28.6093 | 27.9842 | 27.8371 | 27.3291 | 28.3092 | 28.4916 | 28.1489 | 27.0751 | 26.6364 | 26.9517 | 24.2616 | 24.1715 | 24.5021 |
| GmVQ40 | 33.7531 | 33.6888 | 33.6175 | 32.7168 | 32.5494 | 32.2777 | 33.7416 | 33.9455 | 33.7007 | 33.3331 | 32.9717 | 33.1234 | 31.4577 | 31.2526 | 31.2523 |
| GmVQ46 | 33.7672 | 33.8305 | 33.6781 | 32.4741 | 32.1463 | 32.3161 | 33.2180 | 33.2402 | 33.2563 | 32.5979 | 32.4809 | 32.6439 | 31.3792 | 31.4129 | 31.4221 |
| GmVQ48 | 28.7308 | 28.5985 | 28.8085 | 27.7260 | 27.2380 | 27.7635 | 27.7854 | 27.5917 | 27.7161 | 25.4990 | 25.5955 | 25.3235 | 28.0745 | 27.9274 | 27.4194 |
| GmVQ53 | 28.7629 | 28.5933 | 28.6523 | 27.7437 | 28.1779 | 28.2585 | 28.3138 | 28.4961 | 28.3920 | 27.0796 | 26.9605 | 26.9562 | 27.7059 | 27.9331 | 27.8003 |
| GmVQ58 | 28.5834 | 28.6303 | 28.5315 | 28.0714 | 27.7100 | 27.8617 | 29.1684 | 28.7897 | 28.8099 | 27.7712 | 28.1054 | 27.5024 | 26.5227 | 26.6266 | 26.7175 |
| GmVQ59 | 33.6407 | 33.6876 | 33.7296 | 32.6858 | 32.8703 | 32.6782 | 33.8421 | 33.9790 | 33.7855 | 31.0717 | 31.2422 | 31.0589 | 31.8909 | 31.9544 | 31.9484 |
| GmVQ64 | 33.7301 | 33.7771 | 33.8190 | 32.7765 | 32.4330 | 32.9570 | 32.4381 | 32.4424 | 32.5309 | 32.2583 | 32.3838 | 33.0332 | 33.1479 | 33.3338 | 33.4808 |
| GmVQ65 | 31.9423 | 32.1189 | 32.0220 | 30.8087 | 30.5971 | 31.1651 | 32.1731 | 31.5539 | 31.9831 | 29.4064 | 29.4933 | 29.4204 | 29.8519 | 29.8309 | 29.4451 |
| GmVQ68 | 28.6307 | 28.8186 | 28.7412 | 30.0250 | 29.6651 | 30.0107 | 32.1274 | 31.6223 | 31.8902 | 29.3411 | 29.8868 | 29.8689 | 29.0591 | 29.0519 | 28.9105 |
| GmVQ70 | 28.6733 | 28.7230 | 28.6981 | 29.5136 | 29.4688 | 29.7596 | 31.3520 | 31.4893 | 31.3186 | 28.7765 | 28.7721 | 28.6712 | 29.0959 | 29.4030 | 28.9894 |
| GmVQ71 | 28.5986 | 28.6483 | 28.7483 | 29.9475 | 29.7851 | 29.6660 | 30.3563 | 30.3458 | 30.5953 | 30.1532 | 29.7934 | 30.1389 | 29.1033 | 29.6490 | 29.2578 |
| GmVQ74 | 33.7119 | 33.5796 | 33.7896 | 32.6878 | 32.4297 | 32.0727 | 34.2216 | 34.0052 | 34.2591 | 30.8028 | 30.9402 | 31.0388 | 30.3185 | 30.4150 | 30.3464 |
