# Supplementary material for: Genome-wide identification and expression analysis of the VQ gene family in soybean (Glycine max)
Source: PeerJ. 2019 Aug 21;7:e7509. doi: 10.7717/peerj.7509 (PMC6708371; doi:10.7717/peerj.7509)
Supplement: Table S5 [file peerj-07-7509-s007.docx]

| Table S5 Raw data for the salt stress | | | | | | | | | | | | | | | |
| --- | --- | --- | --- | --- | --- | --- | --- | --- | --- | --- | --- | --- | --- | --- | --- |
| Gene | 0h | | | 1h | | | 6h | | | 12h | | | 24h | | |
| actin | 24.7424 | 25.4777 | 25.8974 | 24.7255 | 24.7774 | 24.6982 | 26.0245 | 25.7375 | 26.9825 | 24.8577 | 25.1777 | 24.7789 | 24.7734 | 25.1775 | 24.3784 |
| GmVQ2 | 28.6127 | 28.6596 | 28.5608 | 28.9050 | 29.1092 | 28.8941 | 30.4479 | 29.8531 | 30.2131 | 28.0295 | 27.7248 | 27.8541 | 27.4198 | 27.3213 | 27.3674 |
| GmVQ5 | 30.5869 | 30.6338 | 30.6758 | 29.0695 | 29.5970 | 29.6818 | 30.4353 | 30.2800 | 30.5640 | 27.8984 | 28.1271 | 28.0920 | 27.6186 | 27.4621 | 27.5227 |
| GmVQ6 | 28.6408 | 28.6877 | 28.7297 | 25.4953 | 25.5565 | 25.5027 | 26.8336 | 26.9899 | 26.7476 | 27.1039 | 26.6652 | 26.9805 | 27.6225 | 27.9574 | 27.7099 |
| GmVQ7 | 32.2660 | 32.4457 | 32.6437 | 31.3381 | 31.1878 | 30.8308 | 32.9796 | 33.0905 | 32.9053 | 29.5687 | 29.6982 | 29.7968 | 29.0759 | 29.1730 | 29.1044 |
| GmVQ8 | 32.6090 | 32.7856 | 32.6887 | 31.9789 | 31.9504 | 31.6240 | 33.0861 | 33.2739 | 33.3615 | 32.0112 | 32.1220 | 31.9585 | 30.9871 | 30.8411 | 30.9710 |
| GmVQ9 | 28.5838 | 28.7717 | 28.6943 | 29.8337 | 30.1264 | 29.8003 | 30.1794 | 30.2934 | 30.2557 | 29.1081 | 29.1282 | 29.0066 | 28.7563 | 28.6710 | 28.6190 |
| GmVQ21 | 30.6151 | 30.6648 | 30.6399 | 31.3627 | 31.7357 | 31.1782 | 33.8457 | 33.0495 | 33.7490 | 30.6522 | 30.7782 | 30.9497 | 29.0092 | 28.9607 | 29.3025 |
| GmVQ23 | 28.6273 | 28.6770 | 28.7770 | 28.6212 | 28.4869 | 28.4583 | 29.9746 | 29.9721 | 30.0400 | 28.8240 | 29.1911 | 28.9149 | 28.6639 | 28.3471 | 28.7057 |
| GmVQ27 | 31.3480 | 31.5268 | 31.4482 | 31.4231 | 31.5364 | 31.4987 | 33.8963 | 33.9559 | 33.7612 | 31.5819 | 31.9490 | 31.9202 | 31.5206 | 31.3840 | 31.4876 |
| GmVQ28 | 32.1819 | 32.1797 | 32.0184 | 30.8087 | 30.5994 | 31.0542 | 31.8154 | 31.8459 | 31.9136 | 31.2471 | 31.1609 | 31.1618 | 30.5375 | 30.5766 | 30.6520 |
| GmVQ29 | 28.5993 | 28.8971 | 28.7270 | 28.8049 | 28.6730 | 28.7378 | 30.7600 | 30.6316 | 30.8344 | 27.9847 | 27.8003 | 27.8226 | 27.8061 | 27.6878 | 27.7312 |
| GmVQ31 | 28.5707 | 28.7332 | 28.7223 | 27.5724 | 27.6314 | 27.3418 | 28.7142 | 28.5786 | 28.7769 | 27.4541 | 27.5634 | 27.6502 | 25.9499 | 25.8931 | 25.7835 |
| GmVQ33 | 28.6280 | 28.6667 | 28.6960 | 29.7910 | 29.7450 | 30.0613 | 30.9022 | 30.9731 | 30.8545 | 29.2245 | 29.5316 | 29.7844 | 28.4345 | 28.7487 | 28.5135 |
| GmVQ40 | 33.6067 | 33.7677 | 33.7068 | 32.2546 | 32.1040 | 32.0982 | 32.8793 | 32.8447 | 32.9361 | 32.7845 | 32.6341 | 32.2771 | 32.7498 | 32.8607 | 32.6755 |
| GmVQ46 | 33.7968 | 33.9578 | 33.7274 | 29.9048 | 29.8335 | 29.8906 | 31.9230 | 31.9195 | 31.7770 | 31.0417 | 31.2704 | 30.9354 | 31.0591 | 30.8691 | 30.9421 |
| GmVQ48 | 28.7720 | 28.7077 | 28.6363 | 28.1502 | 28.2965 | 27.9601 | 29.8506 | 30.0569 | 29.6398 | 28.5742 | 28.8492 | 28.9136 | 27.7944 | 27.6100 | 27.7393 |
| GmVQ53 | 28.6536 | 28.7169 | 28.5645 | 27.3515 | 27.1423 | 27.5970 | 28.3583 | 28.3888 | 28.4565 | 26.4860 | 26.4505 | 26.4509 | 27.0804 | 27.1194 | 27.1948 |
| GmVQ58 | 28.6546 | 28.5223 | 28.7323 | 27.9556 | 27.8085 | 27.3005 | 26.0527 | 26.0655 | 26.0993 | 26.9724 | 27.1529 | 26.8102 | 26.8848 | 26.4461 | 26.7614 |
| GmVQ59 | 33.7871 | 33.6175 | 33.6765 | 31.0929 | 31.1867 | 31.1042 | 31.9316 | 31.9043 | 31.7553 | 32.0295 | 31.5654 | 31.9822 | 32.2575 | 32.5106 | 32.1834 |
| GmVQ64 | 33.7301 | 33.7771 | 33.6782 | 32.9687 | 32.9402 | 32.6138 | 33.3381 | 33.4478 | 33.4969 | 32.1644 | 32.2254 | 32.1347 | 31.0782 | 30.9980 | 31.0695 |
| GmVQ65 | 32.1018 | 32.1515 | 32.1267 | 32.0540 | 31.9198 | 31.8912 | 33.4075 | 33.4049 | 33.4729 | 33.1029 | 33.3171 | 33.2707 | 32.1401 | 32.4162 | 32.3771 |
| GmVQ68 | 28.6747 | 28.7244 | 28.8244 | 30.4132 | 30.3676 | 30.5462 | 29.6826 | 29.9286 | 29.9001 | 29.1549 | 29.1751 | 29.0534 | 29.8433 | 30.4052 | 30.3427 |
| GmVQ70 | 28.6772 | 28.6991 | 28.6803 | 29.0008 | 28.9542 | 28.7196 | 30.1988 | 30.2740 | 30.3051 | 30.1863 | 29.8265 | 30.1720 | 29.1364 | 29.6821 | 29.2909 |
| GmVQ71 | 28.7203 | 28.7181 | 28.5568 | 28.6363 | 28.7497 | 28.7119 | 30.1988 | 30.1134 | 30.0614 | 28.5962 | 28.9104 | 28.6751 | 28.1725 | 28.1684 | 28.3114 |
| GmVQ74 | 33.5519 | 33.8497 | 33.6796 | 31.5815 | 31.6065 | 31.9662 | 33.5452 | 33.2131 | 33.5410 | 32.3785 | 32.2356 | 32.3026 | 32.6224 | 32.9573 | 32.7098 |
