# Supplementary material for: Genome-wide identification and expression analysis of the VQ gene family in soybean (Glycine max)
Source: PeerJ. 2019 Aug 21;7:e7509. doi: 10.7717/peerj.7509 (PMC6708371; doi:10.7717/peerj.7509)
Supplement: Table S6 [file peerj-07-7509-s008.docx]

| Table S6 Raw data for the drought stress | | | | | | | | | | | | | | | |
| --- | --- | --- | --- | --- | --- | --- | --- | --- | --- | --- | --- | --- | --- | --- | --- |
| Gene | 0h | | | 1h | | | 6h | | | 12h | | | 24h | | |
| actin | 24.7424 | 25.4777 | 25.8974 | 24.7255 | 24.7774 | 24.6982 | 26.0245 | 25.7375 | 26.9825 | 24.8577 | 25.1777 | 24.7789 | 24.7734 | 25.1775 | 24.3784 |
| GmVQ2 | 28.5992 | 28.6489 | 28.7489 | 27.5521 | 27.4017 | 27.0447 | 27.7453 | 27.8730 | 27.8016 | 26.0746 | 25.9122 | 26.0108 | 25.6725 | 25.6312 | 25.5503 |
| GmVQ5 | 30.6190 | 30.6409 | 30.6220 | 30.5674 | 30.2505 | 30.6091 | 32.1257 | 32.4994 | 32.2013 | 30.2403 | 30.4843 | 30.3027 | 30.6101 | 30.2932 | 30.6518 |
| GmVQ6 | 28.7490 | 28.7468 | 28.5855 | 27.0979 | 26.9621 | 27.1013 | 28.4809 | 28.5659 | 28.5679 | 27.0025 | 26.9132 | 27.2060 | 27.5393 | 27.8608 | 27.6756 |
| GmVQ7 | 32.3099 | 32.6077 | 32.4376 | 31.1337 | 31.0046 | 31.0899 | 32.0190 | 31.8790 | 31.9341 | 31.3744 | 31.3895 | 31.2146 | 31.6042 | 31.6700 | 31.4349 |
| GmVQ8 | 32.7475 | 32.8284 | 32.8030 | 31.9789 | 31.7194 | 31.6240 | 33.0861 | 33.2739 | 33.4831 | 31.0529 | 31.2356 | 31.1449 | 31.8036 | 31.5556 | 31.3985 |
| GmVQ9 | 28.6573 | 28.7853 | 28.8972 | 28.2140 | 28.4689 | 28.2638 | 30.1663 | 29.9331 | 30.0648 | 28.6432 | 28.7068 | 28.8605 | 28.8590 | 28.8300 | 28.5955 |
| GmVQ21 | 30.5529 | 30.7139 | 30.6530 | 29.5081 | 29.5970 | 29.4673 | 30.4353 | 30.0536 | 29.9629 | 28.2241 | 28.1617 | 28.0920 | 28.2906 | 28.0487 | 28.2216 |
| GmVQ23 | 28.7074 | 28.8684 | 28.6381 | 27.6098 | 27.5236 | 27.5245 | 28.2797 | 28.1780 | 28.2256 | 28.5554 | 28.8300 | 28.8949 | 28.3275 | 28.7760 | 28.5518 |
| GmVQ27 | 31.5112 | 31.4469 | 31.3755 | 31.7544 | 32.0046 | 31.4733 | 32.9524 | 33.0276 | 33.0084 | 32.4762 | 32.5801 | 32.9259 | 31.8900 | 32.4357 | 32.0445 |
| GmVQ28 | 32.1107 | 32.1740 | 32.0216 | 32.0250 | 32.2236 | 32.1267 | 33.1562 | 33.3147 | 33.1847 | 31.9547 | 32.2650 | 32.7009 | 32.5718 | 32.0480 | 32.2876 |
| GmVQ29 | 28.7594 | 28.6271 | 28.8371 | 24.8628 | 24.7915 | 24.8486 | 26.8810 | 26.8775 | 26.7350 | 25.9997 | 26.2284 | 25.8934 | 25.4735 | 25.5634 | 25.6762 |
| GmVQ31 | 28.7915 | 28.6219 | 28.6809 | 28.4775 | 28.9038 | 28.6116 | 29.8362 | 29.8555 | 29.6254 | 28.5597 | 28.8348 | 28.8992 | 27.5659 | 27.5956 | 27.5183 |
| GmVQ33 | 28.6120 | 28.6589 | 28.5601 | 27.9842 | 27.8422 | 27.3291 | 27.5409 | 27.2473 | 27.3234 | 27.0010 | 26.7611 | 26.8390 | 26.9134 | 26.4747 | 26.9804 |
| GmVQ40 | 33.6407 | 33.6876 | 33.7296 | 33.9964 | 34.2466 | 33.7152 | 35.7264 | 35.8365 | 36.0338 | 35.1819 | 34.8220 | 35.1678 | 34.1320 | 34.6777 | 34.2865 |
| GmVQ46 | 33.7301 | 33.7771 | 33.8190 | 34.0236 | 34.2267 | 34.0116 | 36.2433 | 35.6425 | 35.8839 | 33.8530 | 33.8215 | 33.5027 | 33.7225 | 33.5114 | 33.4611 |
| GmVQ48 | 28.5282 | 28.7048 | 28.6079 | 27.2446 | 26.6254 | 26.9850 | 29.1728 | 28.2320 | 28.7894 | 27.6539 | 27.1852 | 27.3245 | 27.6412 | 27.9761 | 27.7286 |
| GmVQ53 | 28.5590 | 28.7470 | 28.6695 | 29.3264 | 29.6598 | 29.1900 | 31.7663 | 31.5177 | 31.6275 | 28.4717 | 28.7025 | 28.9078 | 29.0118 | 28.6610 | 28.7468 |
| GmVQ58 | 28.6116 | 28.6613 | 28.6364 | 28.3349 | 28.4359 | 28.3225 | 29.5778 | 29.6551 | 29.7954 | 29.0502 | 29.0703 | 28.9487 | 28.9462 | 28.6131 | 28.7845 |
| GmVQ59 | 33.6478 | 33.7288 | 33.7033 | 33.5813 | 33.4063 | 33.6907 | 35.6010 | 35.8895 | 35.4523 | 34.6099 | 34.7100 | 34.8301 | 33.4632 | 33.7774 | 33.5421 |
| GmVQ64 | 33.7461 | 33.8742 | 33.9861 | 33.3003 | 33.6108 | 33.5515 | 35.2310 | 35.4485 | 35.3029 | 34.4722 | 34.3770 | 33.9468 | 35.0891 | 34.4370 | 34.7843 |
| GmVQ65 | 32.1861 | 32.1218 | 32.0504 | 31.8251 | 31.9262 | 31.8127 | 33.6118 | 33.5053 | 33.6880 | 32.5404 | 32.5606 | 32.4389 | 32.4365 | 32.1033 | 32.2748 |
| GmVQ68 | 28.7252 | 28.7885 | 28.6361 | 27.7333 | 27.9159 | 27.8413 | 29.6826 | 29.9286 | 30.0874 | 29.1549 | 29.1751 | 29.0534 | 29.8433 | 30.4052 | 30.3427 |
| GmVQ70 | 28.7163 | 28.5840 | 28.7940 | 28.3553 | 28.6300 | 28.6948 | 30.7169 | 31.1957 | 30.7914 | 29.2599 | 29.2055 | 29.0036 | 29.2073 | 29.1824 | 29.6916 |
| GmVQ71 | 28.6441 | 28.6660 | 28.6330 | 28.3222 | 28.5969 | 28.6617 | 30.3959 | 30.2189 | 30.4566 | 28.8518 | 28.9189 | 28.7477 | 29.5236 | 29.4920 | 29.6585 |
| GmVQ74 | 33.6272 | 33.6769 | 33.7769 | 33.9039 | 34.0387 | 33.7406 | 35.2275 | 35.1421 | 35.0901 | 33.6249 | 33.9391 | 33.7038 | 33.2012 | 33.0497 | 33.1788 |
