# Supplementary material for: Genome-wide identification and expression analysis of the VQ gene family in soybean (Glycine max)
Source: PeerJ. 2019 Aug 21;7:e7509. doi: 10.7717/peerj.7509 (PMC6708371; doi:10.7717/peerj.7509)
Supplement: Table S7 [file peerj-07-7509-s009.docx]

| Table S7 Raw data for the cold stress | | | | | | | | | | | | | | | |
| --- | --- | --- | --- | --- | --- | --- | --- | --- | --- | --- | --- | --- | --- | --- | --- |
| Gene | 0h | | | 1h | | | 6h | | | 12h | | | 24h | | |
| actin | 24.7424 | 25.4777 | 25.8974 | 24.7255 | 24.7774 | 24.6982 | 26.0245 | 25.7375 | 26.9825 | 24.8577 | 25.1777 | 24.7789 | 24.7734 | 25.1775 | 24.3784 |
| GmVQ2 | 28.7352 | 28.7849 | 28.4770 | 27.5527 | 27.6478 | 27.4986 | 28.9575 | 29.0100 | 29.0430 | 26.8342 | 26.9669 | 26.7999 | 27.0350 | 26.9865 | 26.7641 |
| GmVQ5 | 30.5503 | 30.7300 | 30.6395 | 31.7793 | 32.0720 | 31.7459 | 32.1250 | 32.4994 | 32.2013 | 31.0537 | 31.0738 | 30.9522 | 30.3062 | 30.6166 | 30.3712 |
| GmVQ6 | 28.6133 | 28.7743 | 28.6938 | 28.1212 | 28.3668 | 28.3383 | 29.8319 | 29.7665 | 29.7263 | 28.6250 | 28.9392 | 28.7039 | 28.2013 | 28.1972 | 28.1789 |
| GmVQ7 | 32.3619 | 32.5416 | 32.4518 | 28.5734 | 28.5021 | 28.5592 | 30.5916 | 30.5881 | 30.4456 | 29.7103 | 29.9390 | 29.6039 | 29.7276 | 29.5376 | 29.6107 |
| GmVQ8 | 32.7082 | 32.7579 | 32.9140 | 32.7207 | 32.5865 | 32.7625 | 34.2791 | 34.6528 | 34.3547 | 32.7246 | 33.0388 | 32.8035 | 32.7635 | 32.6292 | 32.8053 |
| GmVQ9 | 28.5350 | 28.7887 | 28.7593 | 26.9132 | 26.8393 | 26.8870 | 28.4897 | 28.5310 | 28.6184 | 25.9528 | 25.8749 | 25.8465 | 25.9702 | 25.7802 | 25.8533 |
| GmVQ21 | 30.7241 | 30.6219 | 30.5737 | 31.7793 | 32.0720 | 31.9535 | 31.9202 | 32.0185 | 31.9861 | 31.0537 | 31.0738 | 30.9522 | 31.7421 | 32.3040 | 32.2415 |
| GmVQ23 | 28.7657 | 28.7664 | 28.5494 | 28.3510 | 28.6256 | 28.6905 | 30.7126 | 30.8042 | 30.7871 | 28.3332 | 28.3788 | 28.4134 | 29.5524 | 29.5207 | 29.6873 |
| GmVQ27 | 31.3286 | 31.5083 | 31.5184 | 32.1745 | 32.5475 | 31.9900 | 33.3307 | 33.4944 | 33.6044 | 31.6755 | 31.5901 | 31.5381 | 29.8211 | 29.7725 | 29.5502 |
| GmVQ28 | 32.1671 | 32.0564 | 32.1564 | 29.0830 | 28.9894 | 28.9356 | 30.2665 | 30.2630 | 30.3308 | 30.5367 | 30.0980 | 30.4133 | 30.1029 | 30.2074 | 30.3237 |
| GmVQ29 | 28.8653 | 28.7631 | 28.5952 | 27.4781 | 27.5832 | 27.3879 | 28.2236 | 28.4121 | 28.4779 | 27.5500 | 27.6816 | 27.4786 | 27.7973 | 27.5747 | 27.7230 |
| GmVQ31 | 28.7823 | 28.6801 | 28.6319 | 26.8104 | 26.6109 | 26.6660 | 28.3512 | 28.2176 | 28.2871 | 27.6395 | 27.7446 | 27.7697 | 27.6268 | 27.8169 | 27.7142 |
| GmVQ33 | 28.5759 | 28.7556 | 28.6635 | 27.5393 | 27.4949 | 27.4957 | 29.0097 | 28.8416 | 28.9194 | 27.3710 | 27.4879 | 27.5136 | 27.5301 | 27.3686 | 27.6575 |
| GmVQ40 | 33.6484 | 33.6981 | 33.7347 | 31.2993 | 31.1561 | 31.1962 | 33.3467 | 33.2131 | 33.2827 | 32.6350 | 32.7402 | 32.7653 | 32.6224 | 32.8125 | 32.7098 |
| GmVQ46 | 33.9066 | 33.8044 | 33.6385 | 32.9687 | 32.8201 | 32.6138 | 34.0759 | 34.2637 | 34.1352 | 32.1644 | 32.3644 | 32.4077 | 31.9769 | 31.8309 | 32.1588 |
| GmVQ48 | 28.5268 | 28.7065 | 28.9045 | 27.4495 | 27.5546 | 27.3593 | 28.1411 | 28.1526 | 28.2085 | 27.7031 | 27.7497 | 27.7018 | 27.7687 | 27.8717 | 27.6944 |
| GmVQ53 | 28.4851 | 28.6617 | 28.8617 | 27.5559 | 27.4056 | 27.0486 | 29.1974 | 28.8867 | 29.1231 | 25.7865 | 25.9160 | 26.0146 | 25.2937 | 25.3908 | 25.3222 |
| GmVQ58 | 28.3981 | 28.8747 | 28.6364 | 28.9391 | 29.1893 | 28.6579 | 30.6691 | 30.7793 | 30.8255 | 30.1246 | 29.7648 | 30.1103 | 29.0747 | 29.6204 | 29.2292 |
| GmVQ59 | 33.6689 | 33.7186 | 33.6937 | 32.5682 | 32.5177 | 32.6212 | 33.5863 | 33.5486 | 33.4806 | 32.8141 | 32.7279 | 32.7288 | 31.9020 | 32.0710 | 32.1426 |
| GmVQ64 | 33.7167 | 33.7664 | 33.8664 | 33.2208 | 33.3671 | 33.0307 | 34.9212 | 35.1275 | 34.7104 | 33.6448 | 33.9198 | 33.9842 | 32.3796 | 32.4042 | 32.3401 |
| GmVQ65 | 31.9702 | 32.4499 | 31.9600 | 32.3660 | 32.0607 | 32.3551 | 33.4496 | 33.3140 | 33.6740 | 31.2592 | 31.1858 | 31.5564 | 30.3486 | 30.2203 | 30.2514 |
| GmVQ68 | 28.7964 | 28.7942 | 28.6329 | 27.3979 | 27.2226 | 27.5953 | 27.6170 | 27.6526 | 27.7489 | 27.0772 | 27.2577 | 26.9150 | 26.4546 | 26.2894 | 26.3683 |
| GmVQ70 | 28.5563 | 28.8541 | 28.6840 | 29.8242 | 29.8763 | 30.8552 | 30.9354 | 31.3084 | 30.7509 | 29.2576 | 29.2469 | 29.1510 | 28.4677 | 28.7818 | 28.5466 |
| GmVQ71 | 28.5376 | 28.7001 | 28.6892 | 29.7910 | 29.4077 | 30.0613 | 30.9022 | 31.2752 | 30.7178 | 29.2245 | 29.5316 | 29.7844 | 28.4345 | 28.7487 | 28.5135 |
| GmVQ74 | 33.6778 | 33.7411 | 33.5887 | 33.0129 | 33.1440 | 32.5478 | 32.9846 | 33.0322 | 32.7014 | 32.0297 | 32.2102 | 31.8675 | 31.9421 | 31.5034 | 31.8187 |
