# Supplementary material for: Genome-wide identification and expression analysis of the VQ gene family in soybean (Glycine max)
Source: PeerJ. 2019 Aug 21;7:e7509. doi: 10.7717/peerj.7509 (PMC6708371; doi:10.7717/peerj.7509)
Supplement: Table S8 [file peerj-07-7509-s010.docx]

| **Table S8 GO terms of the *GmVQ* genes** | | | | | | | |
| --- | --- | --- | --- | --- | --- | --- | --- |
| GO ID | GO Term | Ontology | GeneRatio | BgRatio | Pvalue | FDR | geneID |
| GO:0008150 | biological_process | BP | 39 | 39 | 1 | 1 | GmVQ1;GmVQ2;GmVQ4;GmVQ6;GmVQ7;GmVQ8;GmVQ9;GmVQ12;GmVQ13;GmVQ14;GmVQ18;GmVQ19;GmVQ21;GmVQ24;GmVQ25;GmVQ26;GmVQ28;GmVQ29;GmVQ30;GmVQ34;GmVQ35;GmVQ36;GmVQ39;GmVQ40;GmVQ41;GmVQ45;GmVQ49;GmVQ50;GmVQ52;GmVQ53;GmVQ54;GmVQ56;GmVQ57;GmVQ59;GmVQ61;GmVQ63;GmVQ65;GmVQ68;GmVQ71 |
| GO:0050789 | regulation of biological process | BP | 34 | 34 | 1 | 1 | GmVQ1;GmVQ2;GmVQ4;GmVQ6;GmVQ7;GmVQ8;GmVQ9;GmVQ12;GmVQ13;GmVQ14;GmVQ19;GmVQ21;GmVQ24;GmVQ25;GmVQ26;GmVQ28;GmVQ29;GmVQ30;GmVQ35;GmVQ39;GmVQ40;GmVQ41;GmVQ45;GmVQ49;GmVQ50;GmVQ52;GmVQ53;GmVQ54;GmVQ56;GmVQ57;GmVQ59;GmVQ61;GmVQ63;GmVQ71 |
| GO:0065007 | biological regulation | BP | 34 | 34 | 1 | 1 | GmVQ1;GmVQ2;GmVQ4;GmVQ6;GmVQ7;GmVQ8;GmVQ9;GmVQ12;GmVQ13;GmVQ14;GmVQ19;GmVQ21;GmVQ24;GmVQ25;GmVQ26;GmVQ28;GmVQ29;GmVQ30;GmVQ35;GmVQ39;GmVQ40;GmVQ41;GmVQ45;GmVQ49;GmVQ50;GmVQ52;GmVQ53;GmVQ54;GmVQ56;GmVQ57;GmVQ59;GmVQ61;GmVQ63;GmVQ71 |
| GO:0009987 | cellular process | BP | 31 | 31 | 1 | 1 | GmVQ6;GmVQ7;GmVQ8;GmVQ12;GmVQ13;GmVQ14;GmVQ18;GmVQ19;GmVQ24;GmVQ25;GmVQ26;GmVQ28;GmVQ29;GmVQ30;GmVQ34;GmVQ35;GmVQ36;GmVQ45;GmVQ49;GmVQ50;GmVQ52;GmVQ53;GmVQ54;GmVQ56;GmVQ57;GmVQ59;GmVQ61;GmVQ63;GmVQ65;GmVQ68;GmVQ71 |
| GO:0050794 | regulation of cellular process | BP | 25 | 25 | 1 | 1 | GmVQ6;GmVQ7;GmVQ8;GmVQ12;GmVQ13;GmVQ14;GmVQ19;GmVQ24;GmVQ25;GmVQ26;GmVQ28;GmVQ29;GmVQ35;GmVQ45;GmVQ49;GmVQ50;GmVQ52;GmVQ53;GmVQ54;GmVQ56;GmVQ57;GmVQ59;GmVQ61;GmVQ63;GmVQ71 |
| GO:0050896 | response to stimulus | BP | 25 | 25 | 1 | 1 | GmVQ4;GmVQ7;GmVQ9;GmVQ13;GmVQ14;GmVQ19;GmVQ21;GmVQ24;GmVQ25;GmVQ26;GmVQ28;GmVQ29;GmVQ30;GmVQ35;GmVQ36;GmVQ45;GmVQ49;GmVQ50;GmVQ52;GmVQ53;GmVQ56;GmVQ57;GmVQ59;GmVQ61;GmVQ65 |
| GO:0006950 | response to stress | BP | 23 | 23 | 1 | 1 | GmVQ4;GmVQ7;GmVQ9;GmVQ13;GmVQ14;GmVQ19;GmVQ21;GmVQ24;GmVQ25;GmVQ26;GmVQ28;GmVQ29;GmVQ30;GmVQ35;GmVQ45;GmVQ49;GmVQ50;GmVQ52;GmVQ53;GmVQ56;GmVQ57;GmVQ59;GmVQ61 |
| GO:0008152 | metabolic process | BP | 21 | 21 | 1 | 1 | GmVQ6;GmVQ8;GmVQ12;GmVQ13;GmVQ18;GmVQ19;GmVQ26;GmVQ29;GmVQ30;GmVQ34;GmVQ35;GmVQ49;GmVQ50;GmVQ53;GmVQ54;GmVQ56;GmVQ57;GmVQ61;GmVQ63;GmVQ68;GmVQ71 |
| GO:0044237 | cellular metabolic process | BP | 21 | 21 | 1 | 1 | GmVQ6;GmVQ8;GmVQ12;GmVQ13;GmVQ18;GmVQ19;GmVQ26;GmVQ29;GmVQ30;GmVQ34;GmVQ35;GmVQ49;GmVQ50;GmVQ53;GmVQ54;GmVQ56;GmVQ57;GmVQ61;GmVQ63;GmVQ68;GmVQ71 |
| GO:0048583 | regulation of response to stimulus | BP | 21 | 21 | 1 | 1 | GmVQ4;GmVQ7;GmVQ9;GmVQ13;GmVQ14;GmVQ19;GmVQ21;GmVQ24;GmVQ25;GmVQ26;GmVQ29;GmVQ30;GmVQ35;GmVQ49;GmVQ50;GmVQ52;GmVQ53;GmVQ56;GmVQ57;GmVQ59;GmVQ61 |
| GO:0080134 | regulation of response to stress | BP | 21 | 21 | 1 | 1 | GmVQ4;GmVQ7;GmVQ9;GmVQ13;GmVQ14;GmVQ19;GmVQ21;GmVQ24;GmVQ25;GmVQ26;GmVQ29;GmVQ30;GmVQ35;GmVQ49;GmVQ50;GmVQ52;GmVQ53;GmVQ56;GmVQ57;GmVQ59;GmVQ61 |
| GO:0006952 | defense response | BP | 20 | 20 | 1 | 1 | GmVQ7;GmVQ13;GmVQ14;GmVQ19;GmVQ24;GmVQ25;GmVQ26;GmVQ28;GmVQ29;GmVQ30;GmVQ35;GmVQ45;GmVQ49;GmVQ50;GmVQ52;GmVQ53;GmVQ56;GmVQ57;GmVQ59;GmVQ61 |
| GO:0044699 | single-organism process | BP | 20 | 20 | 1 | 1 | GmVQ1;GmVQ2;GmVQ7;GmVQ13;GmVQ14;GmVQ19;GmVQ24;GmVQ25;GmVQ28;GmVQ30;GmVQ35;GmVQ36;GmVQ39;GmVQ40;GmVQ41;GmVQ45;GmVQ52;GmVQ56;GmVQ59;GmVQ65 |
| GO:0031347 | regulation of defense response | BP | 18 | 18 | 1 | 1 | GmVQ7;GmVQ13;GmVQ14;GmVQ19;GmVQ24;GmVQ25;GmVQ26;GmVQ29;GmVQ30;GmVQ35;GmVQ49;GmVQ50;GmVQ52;GmVQ53;GmVQ56;GmVQ57;GmVQ59;GmVQ61 |
| GO:0006139 | nucleobase-containing compound metabolic process | BP | 15 | 15 | 1 | 1 | GmVQ6;GmVQ8;GmVQ12;GmVQ26;GmVQ29;GmVQ30;GmVQ49;GmVQ50;GmVQ53;GmVQ54;GmVQ56;GmVQ57;GmVQ61;GmVQ63;GmVQ71 |
| GO:0006725 | cellular aromatic compound metabolic process | BP | 15 | 15 | 1 | 1 | GmVQ6;GmVQ8;GmVQ12;GmVQ26;GmVQ29;GmVQ30;GmVQ49;GmVQ50;GmVQ53;GmVQ54;GmVQ56;GmVQ57;GmVQ61;GmVQ63;GmVQ71 |
| GO:0006807 | nitrogen compound metabolic process | BP | 15 | 15 | 1 | 1 | GmVQ6;GmVQ8;GmVQ12;GmVQ26;GmVQ29;GmVQ30;GmVQ49;GmVQ50;GmVQ53;GmVQ54;GmVQ56;GmVQ57;GmVQ61;GmVQ63;GmVQ71 |
| GO:0009058 | biosynthetic process | BP | 15 | 15 | 1 | 1 | GmVQ6;GmVQ8;GmVQ12;GmVQ26;GmVQ29;GmVQ30;GmVQ49;GmVQ50;GmVQ53;GmVQ54;GmVQ56;GmVQ57;GmVQ61;GmVQ63;GmVQ71 |
| GO:0010467 | gene expression | BP | 15 | 15 | 1 | 1 | GmVQ6;GmVQ8;GmVQ12;GmVQ26;GmVQ29;GmVQ30;GmVQ49;GmVQ50;GmVQ53;GmVQ54;GmVQ56;GmVQ57;GmVQ61;GmVQ63;GmVQ71 |
| GO:0010468 | regulation of gene expression | BP | 15 | 15 | 1 | 1 | GmVQ6;GmVQ8;GmVQ12;GmVQ26;GmVQ29;GmVQ30;GmVQ49;GmVQ50;GmVQ53;GmVQ54;GmVQ56;GmVQ57;GmVQ61;GmVQ63;GmVQ71 |
| GO:0016070 | RNA metabolic process | BP | 15 | 15 | 1 | 1 | GmVQ6;GmVQ8;GmVQ12;GmVQ26;GmVQ29;GmVQ30;GmVQ49;GmVQ50;GmVQ53;GmVQ54;GmVQ56;GmVQ57;GmVQ61;GmVQ63;GmVQ71 |
| GO:0019222 | regulation of metabolic process | BP | 15 | 15 | 1 | 1 | GmVQ6;GmVQ8;GmVQ12;GmVQ26;GmVQ29;GmVQ30;GmVQ49;GmVQ50;GmVQ53;GmVQ54;GmVQ56;GmVQ57;GmVQ61;GmVQ63;GmVQ71 |
| GO:0034641 | cellular nitrogen compound metabolic process | BP | 15 | 15 | 1 | 1 | GmVQ6;GmVQ8;GmVQ12;GmVQ26;GmVQ29;GmVQ30;GmVQ49;GmVQ50;GmVQ53;GmVQ54;GmVQ56;GmVQ57;GmVQ61;GmVQ63;GmVQ71 |
| GO:0043170 | macromolecule metabolic process | BP | 15 | 15 | 1 | 1 | GmVQ6;GmVQ8;GmVQ12;GmVQ26;GmVQ29;GmVQ30;GmVQ49;GmVQ50;GmVQ53;GmVQ54;GmVQ56;GmVQ57;GmVQ61;GmVQ63;GmVQ71 |
| GO:0044238 | primary metabolic process | BP | 15 | 15 | 1 | 1 | GmVQ6;GmVQ8;GmVQ12;GmVQ26;GmVQ29;GmVQ30;GmVQ49;GmVQ50;GmVQ53;GmVQ54;GmVQ56;GmVQ57;GmVQ61;GmVQ63;GmVQ71 |
| GO:0044260 | cellular macromolecule metabolic process | BP | 15 | 15 | 1 | 1 | GmVQ6;GmVQ8;GmVQ12;GmVQ26;GmVQ29;GmVQ30;GmVQ49;GmVQ50;GmVQ53;GmVQ54;GmVQ56;GmVQ57;GmVQ61;GmVQ63;GmVQ71 |
| GO:0046483 | heterocycle metabolic process | BP | 15 | 15 | 1 | 1 | GmVQ6;GmVQ8;GmVQ12;GmVQ26;GmVQ29;GmVQ30;GmVQ49;GmVQ50;GmVQ53;GmVQ54;GmVQ56;GmVQ57;GmVQ61;GmVQ63;GmVQ71 |
| GO:0048518 | positive regulation of biological process | BP | 15 | 15 | 1 | 1 | GmVQ6;GmVQ7;GmVQ8;GmVQ12;GmVQ13;GmVQ14;GmVQ19;GmVQ24;GmVQ25;GmVQ35;GmVQ52;GmVQ54;GmVQ59;GmVQ63;GmVQ71 |
| GO:0060255 | regulation of macromolecule metabolic process | BP | 15 | 15 | 1 | 1 | GmVQ6;GmVQ8;GmVQ12;GmVQ26;GmVQ29;GmVQ30;GmVQ49;GmVQ50;GmVQ53;GmVQ54;GmVQ56;GmVQ57;GmVQ61;GmVQ63;GmVQ71 |
| GO:0071704 | organic substance metabolic process | BP | 15 | 15 | 1 | 1 | GmVQ6;GmVQ8;GmVQ12;GmVQ26;GmVQ29;GmVQ30;GmVQ49;GmVQ50;GmVQ53;GmVQ54;GmVQ56;GmVQ57;GmVQ61;GmVQ63;GmVQ71 |
| GO:0090304 | nucleic acid metabolic process | BP | 15 | 15 | 1 | 1 | GmVQ6;GmVQ8;GmVQ12;GmVQ26;GmVQ29;GmVQ30;GmVQ49;GmVQ50;GmVQ53;GmVQ54;GmVQ56;GmVQ57;GmVQ61;GmVQ63;GmVQ71 |
| GO:1901360 | organic cyclic compound metabolic process | BP | 15 | 15 | 1 | 1 | GmVQ6;GmVQ8;GmVQ12;GmVQ26;GmVQ29;GmVQ30;GmVQ49;GmVQ50;GmVQ53;GmVQ54;GmVQ56;GmVQ57;GmVQ61;GmVQ63;GmVQ71 |
| GO:0006351 | transcription, DNA-templated | BP | 14 | 14 | 1 | 1 | GmVQ6;GmVQ8;GmVQ12;GmVQ26;GmVQ29;GmVQ49;GmVQ50;GmVQ53;GmVQ54;GmVQ56;GmVQ57;GmVQ61;GmVQ63;GmVQ71 |
| GO:0006355 | regulation of transcription, DNA-templated | BP | 14 | 14 | 1 | 1 | GmVQ6;GmVQ8;GmVQ12;GmVQ26;GmVQ29;GmVQ49;GmVQ50;GmVQ53;GmVQ54;GmVQ56;GmVQ57;GmVQ61;GmVQ63;GmVQ71 |
| GO:0009059 | macromolecule biosynthetic process | BP | 14 | 14 | 1 | 1 | GmVQ6;GmVQ8;GmVQ12;GmVQ26;GmVQ29;GmVQ49;GmVQ50;GmVQ53;GmVQ54;GmVQ56;GmVQ57;GmVQ61;GmVQ63;GmVQ71 |
| GO:0009889 | regulation of biosynthetic process | BP | 14 | 14 | 1 | 1 | GmVQ6;GmVQ8;GmVQ12;GmVQ26;GmVQ29;GmVQ49;GmVQ50;GmVQ53;GmVQ54;GmVQ56;GmVQ57;GmVQ61;GmVQ63;GmVQ71 |
| GO:0010556 | regulation of macromolecule biosynthetic process | BP | 14 | 14 | 1 | 1 | GmVQ6;GmVQ8;GmVQ12;GmVQ26;GmVQ29;GmVQ49;GmVQ50;GmVQ53;GmVQ54;GmVQ56;GmVQ57;GmVQ61;GmVQ63;GmVQ71 |
| GO:0018130 | heterocycle biosynthetic process | BP | 14 | 14 | 1 | 1 | GmVQ6;GmVQ8;GmVQ12;GmVQ26;GmVQ29;GmVQ49;GmVQ50;GmVQ53;GmVQ54;GmVQ56;GmVQ57;GmVQ61;GmVQ63;GmVQ71 |
| GO:0019219 | regulation of nucleobase-containing compound metabolic process | BP | 14 | 14 | 1 | 1 | GmVQ6;GmVQ8;GmVQ12;GmVQ26;GmVQ29;GmVQ49;GmVQ50;GmVQ53;GmVQ54;GmVQ56;GmVQ57;GmVQ61;GmVQ63;GmVQ71 |
| GO:0019438 | aromatic compound biosynthetic process | BP | 14 | 14 | 1 | 1 | GmVQ6;GmVQ8;GmVQ12;GmVQ26;GmVQ29;GmVQ49;GmVQ50;GmVQ53;GmVQ54;GmVQ56;GmVQ57;GmVQ61;GmVQ63;GmVQ71 |
| GO:0031323 | regulation of cellular metabolic process | BP | 14 | 14 | 1 | 1 | GmVQ6;GmVQ8;GmVQ12;GmVQ26;GmVQ29;GmVQ49;GmVQ50;GmVQ53;GmVQ54;GmVQ56;GmVQ57;GmVQ61;GmVQ63;GmVQ71 |
| GO:0031326 | regulation of cellular biosynthetic process | BP | 14 | 14 | 1 | 1 | GmVQ6;GmVQ8;GmVQ12;GmVQ26;GmVQ29;GmVQ49;GmVQ50;GmVQ53;GmVQ54;GmVQ56;GmVQ57;GmVQ61;GmVQ63;GmVQ71 |
| GO:0032774 | RNA biosynthetic process | BP | 14 | 14 | 1 | 1 | GmVQ6;GmVQ8;GmVQ12;GmVQ26;GmVQ29;GmVQ49;GmVQ50;GmVQ53;GmVQ54;GmVQ56;GmVQ57;GmVQ61;GmVQ63;GmVQ71 |
| GO:0034645 | cellular macromolecule biosynthetic process | BP | 14 | 14 | 1 | 1 | GmVQ6;GmVQ8;GmVQ12;GmVQ26;GmVQ29;GmVQ49;GmVQ50;GmVQ53;GmVQ54;GmVQ56;GmVQ57;GmVQ61;GmVQ63;GmVQ71 |
| GO:0034654 | nucleobase-containing compound biosynthetic process | BP | 14 | 14 | 1 | 1 | GmVQ6;GmVQ8;GmVQ12;GmVQ26;GmVQ29;GmVQ49;GmVQ50;GmVQ53;GmVQ54;GmVQ56;GmVQ57;GmVQ61;GmVQ63;GmVQ71 |
| GO:0044249 | cellular biosynthetic process | BP | 14 | 14 | 1 | 1 | GmVQ6;GmVQ8;GmVQ12;GmVQ26;GmVQ29;GmVQ49;GmVQ50;GmVQ53;GmVQ54;GmVQ56;GmVQ57;GmVQ61;GmVQ63;GmVQ71 |
| GO:0044271 | cellular nitrogen compound biosynthetic process | BP | 14 | 14 | 1 | 1 | GmVQ6;GmVQ8;GmVQ12;GmVQ26;GmVQ29;GmVQ49;GmVQ50;GmVQ53;GmVQ54;GmVQ56;GmVQ57;GmVQ61;GmVQ63;GmVQ71 |
| GO:0051090 | regulation of sequence-specific DNA binding transcription factor activity | BP | 14 | 14 | 1 | 1 | GmVQ6;GmVQ8;GmVQ12;GmVQ26;GmVQ29;GmVQ49;GmVQ50;GmVQ53;GmVQ54;GmVQ56;GmVQ57;GmVQ61;GmVQ63;GmVQ71 |
| GO:0051171 | regulation of nitrogen compound metabolic process | BP | 14 | 14 | 1 | 1 | GmVQ6;GmVQ8;GmVQ12;GmVQ26;GmVQ29;GmVQ49;GmVQ50;GmVQ53;GmVQ54;GmVQ56;GmVQ57;GmVQ61;GmVQ63;GmVQ71 |
| GO:0051252 | regulation of RNA metabolic process | BP | 14 | 14 | 1 | 1 | GmVQ6;GmVQ8;GmVQ12;GmVQ26;GmVQ29;GmVQ49;GmVQ50;GmVQ53;GmVQ54;GmVQ56;GmVQ57;GmVQ61;GmVQ63;GmVQ71 |
| GO:0065009 | regulation of molecular function | BP | 14 | 14 | 1 | 1 | GmVQ6;GmVQ8;GmVQ12;GmVQ26;GmVQ29;GmVQ49;GmVQ50;GmVQ53;GmVQ54;GmVQ56;GmVQ57;GmVQ61;GmVQ63;GmVQ71 |
| GO:0080090 | regulation of primary metabolic process | BP | 14 | 14 | 1 | 1 | GmVQ6;GmVQ8;GmVQ12;GmVQ26;GmVQ29;GmVQ49;GmVQ50;GmVQ53;GmVQ54;GmVQ56;GmVQ57;GmVQ61;GmVQ63;GmVQ71 |
| GO:0097659 | nucleic acid-templated transcription | BP | 14 | 14 | 1 | 1 | GmVQ6;GmVQ8;GmVQ12;GmVQ26;GmVQ29;GmVQ49;GmVQ50;GmVQ53;GmVQ54;GmVQ56;GmVQ57;GmVQ61;GmVQ63;GmVQ71 |
| GO:1901362 | organic cyclic compound biosynthetic process | BP | 14 | 14 | 1 | 1 | GmVQ6;GmVQ8;GmVQ12;GmVQ26;GmVQ29;GmVQ49;GmVQ50;GmVQ53;GmVQ54;GmVQ56;GmVQ57;GmVQ61;GmVQ63;GmVQ71 |
| GO:1901576 | organic substance biosynthetic process | BP | 14 | 14 | 1 | 1 | GmVQ6;GmVQ8;GmVQ12;GmVQ26;GmVQ29;GmVQ49;GmVQ50;GmVQ53;GmVQ54;GmVQ56;GmVQ57;GmVQ61;GmVQ63;GmVQ71 |
| GO:1903506 | regulation of nucleic acid-templated transcription | BP | 14 | 14 | 1 | 1 | GmVQ6;GmVQ8;GmVQ12;GmVQ26;GmVQ29;GmVQ49;GmVQ50;GmVQ53;GmVQ54;GmVQ56;GmVQ57;GmVQ61;GmVQ63;GmVQ71 |
| GO:2000112 | regulation of cellular macromolecule biosynthetic process | BP | 14 | 14 | 1 | 1 | GmVQ6;GmVQ8;GmVQ12;GmVQ26;GmVQ29;GmVQ49;GmVQ50;GmVQ53;GmVQ54;GmVQ56;GmVQ57;GmVQ61;GmVQ63;GmVQ71 |
| GO:2001141 | regulation of RNA biosynthetic process | BP | 14 | 14 | 1 | 1 | GmVQ6;GmVQ8;GmVQ12;GmVQ26;GmVQ29;GmVQ49;GmVQ50;GmVQ53;GmVQ54;GmVQ56;GmVQ57;GmVQ61;GmVQ63;GmVQ71 |
| GO:0044763 | single-organism cellular process | BP | 13 | 13 | 1 | 1 | GmVQ7;GmVQ13;GmVQ14;GmVQ19;GmVQ24;GmVQ25;GmVQ28;GmVQ30;GmVQ35;GmVQ45;GmVQ52;GmVQ56;GmVQ59 |
| GO:0051716 | cellular response to stimulus | BP | 13 | 13 | 1 | 1 | GmVQ7;GmVQ13;GmVQ14;GmVQ19;GmVQ24;GmVQ25;GmVQ30;GmVQ35;GmVQ36;GmVQ52;GmVQ56;GmVQ59;GmVQ65 |
| GO:0048519 | negative regulation of biological process | BP | 12 | 12 | 1 | 1 | GmVQ4;GmVQ9;GmVQ21;GmVQ26;GmVQ29;GmVQ30;GmVQ49;GmVQ50;GmVQ53;GmVQ56;GmVQ57;GmVQ61 |
| GO:0048585 | negative regulation of response to stimulus | BP | 11 | 11 | 1 | 1 | GmVQ4;GmVQ9;GmVQ21;GmVQ26;GmVQ29;GmVQ49;GmVQ50;GmVQ53;GmVQ56;GmVQ57;GmVQ61 |
| GO:0002376 | immune system process | BP | 10 | 10 | 1 | 1 | GmVQ7;GmVQ13;GmVQ14;GmVQ19;GmVQ24;GmVQ25;GmVQ30;GmVQ35;GmVQ52;GmVQ59 |
| GO:0002682 | regulation of immune system process | BP | 10 | 10 | 1 | 1 | GmVQ7;GmVQ13;GmVQ14;GmVQ19;GmVQ24;GmVQ25;GmVQ30;GmVQ35;GmVQ52;GmVQ59 |
| GO:0006955 | immune response | BP | 10 | 10 | 1 | 1 | GmVQ7;GmVQ13;GmVQ14;GmVQ19;GmVQ24;GmVQ25;GmVQ30;GmVQ35;GmVQ52;GmVQ59 |
| GO:0007154 | cell communication | BP | 10 | 10 | 1 | 1 | GmVQ7;GmVQ13;GmVQ14;GmVQ19;GmVQ24;GmVQ25;GmVQ35;GmVQ52;GmVQ56;GmVQ59 |
| GO:0007165 | signal transduction | BP | 10 | 10 | 1 | 1 | GmVQ7;GmVQ13;GmVQ14;GmVQ19;GmVQ24;GmVQ25;GmVQ35;GmVQ52;GmVQ56;GmVQ59 |
| GO:0023052 | signaling | BP | 10 | 10 | 1 | 1 | GmVQ7;GmVQ13;GmVQ14;GmVQ19;GmVQ24;GmVQ25;GmVQ35;GmVQ52;GmVQ56;GmVQ59 |
| GO:0032501 | multicellular organismal process | BP | 10 | 10 | 1 | 1 | GmVQ1;GmVQ2;GmVQ28;GmVQ30;GmVQ36;GmVQ39;GmVQ40;GmVQ41;GmVQ45;GmVQ65 |
| GO:0032502 | developmental process | BP | 10 | 10 | 1 | 1 | GmVQ1;GmVQ2;GmVQ28;GmVQ30;GmVQ36;GmVQ39;GmVQ40;GmVQ41;GmVQ45;GmVQ65 |
| GO:0044700 | single organism signaling | BP | 10 | 10 | 1 | 1 | GmVQ7;GmVQ13;GmVQ14;GmVQ19;GmVQ24;GmVQ25;GmVQ35;GmVQ52;GmVQ56;GmVQ59 |
| GO:0044767 | single-organism developmental process | BP | 10 | 10 | 1 | 1 | GmVQ1;GmVQ2;GmVQ28;GmVQ30;GmVQ36;GmVQ39;GmVQ40;GmVQ41;GmVQ45;GmVQ65 |
| GO:0045087 | innate immune response | BP | 10 | 10 | 1 | 1 | GmVQ7;GmVQ13;GmVQ14;GmVQ19;GmVQ24;GmVQ25;GmVQ30;GmVQ35;GmVQ52;GmVQ59 |
| GO:0002218 | activation of innate immune response | BP | 9 | 9 | 1 | 1 | GmVQ7;GmVQ13;GmVQ14;GmVQ19;GmVQ24;GmVQ25;GmVQ35;GmVQ52;GmVQ59 |
| GO:0002253 | activation of immune response | BP | 9 | 9 | 1 | 1 | GmVQ7;GmVQ13;GmVQ14;GmVQ19;GmVQ24;GmVQ25;GmVQ35;GmVQ52;GmVQ59 |
| GO:0002684 | positive regulation of immune system process | BP | 9 | 9 | 1 | 1 | GmVQ7;GmVQ13;GmVQ14;GmVQ19;GmVQ24;GmVQ25;GmVQ35;GmVQ52;GmVQ59 |
| GO:0009870 | defense response signaling pathway, resistance gene-dependent | BP | 9 | 9 | 1 | 1 | GmVQ7;GmVQ13;GmVQ14;GmVQ19;GmVQ24;GmVQ25;GmVQ35;GmVQ52;GmVQ59 |
| GO:0009892 | negative regulation of metabolic process | BP | 9 | 9 | 1 | 1 | GmVQ26;GmVQ29;GmVQ30;GmVQ49;GmVQ50;GmVQ53;GmVQ56;GmVQ57;GmVQ61 |
| GO:0010605 | negative regulation of macromolecule metabolic process | BP | 9 | 9 | 1 | 1 | GmVQ26;GmVQ29;GmVQ30;GmVQ49;GmVQ50;GmVQ53;GmVQ56;GmVQ57;GmVQ61 |
| GO:0010629 | negative regulation of gene expression | BP | 9 | 9 | 1 | 1 | GmVQ26;GmVQ29;GmVQ30;GmVQ49;GmVQ50;GmVQ53;GmVQ56;GmVQ57;GmVQ61 |
| GO:0031349 | positive regulation of defense response | BP | 9 | 9 | 1 | 1 | GmVQ7;GmVQ13;GmVQ14;GmVQ19;GmVQ24;GmVQ25;GmVQ35;GmVQ52;GmVQ59 |
| GO:0045088 | regulation of innate immune response | BP | 9 | 9 | 1 | 1 | GmVQ7;GmVQ13;GmVQ14;GmVQ19;GmVQ24;GmVQ25;GmVQ35;GmVQ52;GmVQ59 |
| GO:0045089 | positive regulation of innate immune response | BP | 9 | 9 | 1 | 1 | GmVQ7;GmVQ13;GmVQ14;GmVQ19;GmVQ24;GmVQ25;GmVQ35;GmVQ52;GmVQ59 |
| GO:0048584 | positive regulation of response to stimulus | BP | 9 | 9 | 1 | 1 | GmVQ7;GmVQ13;GmVQ14;GmVQ19;GmVQ24;GmVQ25;GmVQ35;GmVQ52;GmVQ59 |
| GO:0050776 | regulation of immune response | BP | 9 | 9 | 1 | 1 | GmVQ7;GmVQ13;GmVQ14;GmVQ19;GmVQ24;GmVQ25;GmVQ35;GmVQ52;GmVQ59 |
| GO:0050778 | positive regulation of immune response | BP | 9 | 9 | 1 | 1 | GmVQ7;GmVQ13;GmVQ14;GmVQ19;GmVQ24;GmVQ25;GmVQ35;GmVQ52;GmVQ59 |
| GO:0006968 | cellular defense response | BP | 8 | 8 | 1 | 1 | GmVQ26;GmVQ29;GmVQ49;GmVQ50;GmVQ53;GmVQ56;GmVQ57;GmVQ61 |
| GO:0007275 | multicellular organism development | BP | 8 | 8 | 1 | 1 | GmVQ1;GmVQ2;GmVQ30;GmVQ36;GmVQ39;GmVQ40;GmVQ41;GmVQ65 |
| GO:0009791 | post-embryonic development | BP | 8 | 8 | 1 | 1 | GmVQ1;GmVQ2;GmVQ30;GmVQ36;GmVQ39;GmVQ40;GmVQ41;GmVQ65 |
| GO:0009890 | negative regulation of biosynthetic process | BP | 8 | 8 | 1 | 1 | GmVQ26;GmVQ29;GmVQ49;GmVQ50;GmVQ53;GmVQ56;GmVQ57;GmVQ61 |
| GO:0010185 | regulation of cellular defense response | BP | 8 | 8 | 1 | 1 | GmVQ26;GmVQ29;GmVQ49;GmVQ50;GmVQ53;GmVQ56;GmVQ57;GmVQ61 |
| GO:0010558 | negative regulation of macromolecule biosynthetic process | BP | 8 | 8 | 1 | 1 | GmVQ26;GmVQ29;GmVQ49;GmVQ50;GmVQ53;GmVQ56;GmVQ57;GmVQ61 |
| GO:0031324 | negative regulation of cellular metabolic process | BP | 8 | 8 | 1 | 1 | GmVQ26;GmVQ29;GmVQ49;GmVQ50;GmVQ53;GmVQ56;GmVQ57;GmVQ61 |
| GO:0031327 | negative regulation of cellular biosynthetic process | BP | 8 | 8 | 1 | 1 | GmVQ26;GmVQ29;GmVQ49;GmVQ50;GmVQ53;GmVQ56;GmVQ57;GmVQ61 |
| GO:0031348 | negative regulation of defense response | BP | 8 | 8 | 1 | 1 | GmVQ26;GmVQ29;GmVQ49;GmVQ50;GmVQ53;GmVQ56;GmVQ57;GmVQ61 |
| GO:0043433 | negative regulation of sequence-specific DNA binding transcription factor activity | BP | 8 | 8 | 1 | 1 | GmVQ26;GmVQ29;GmVQ49;GmVQ50;GmVQ53;GmVQ56;GmVQ57;GmVQ61 |
| GO:0044092 | negative regulation of molecular function | BP | 8 | 8 | 1 | 1 | GmVQ26;GmVQ29;GmVQ49;GmVQ50;GmVQ53;GmVQ56;GmVQ57;GmVQ61 |
| GO:0044707 | single-multicellular organism process | BP | 8 | 8 | 1 | 1 | GmVQ1;GmVQ2;GmVQ30;GmVQ36;GmVQ39;GmVQ40;GmVQ41;GmVQ65 |
| GO:0045892 | negative regulation of transcription, DNA-templated | BP | 8 | 8 | 1 | 1 | GmVQ26;GmVQ29;GmVQ49;GmVQ50;GmVQ53;GmVQ56;GmVQ57;GmVQ61 |
| GO:0045934 | negative regulation of nucleobase-containing compound metabolic process | BP | 8 | 8 | 1 | 1 | GmVQ26;GmVQ29;GmVQ49;GmVQ50;GmVQ53;GmVQ56;GmVQ57;GmVQ61 |
| GO:0048523 | negative regulation of cellular process | BP | 8 | 8 | 1 | 1 | GmVQ26;GmVQ29;GmVQ49;GmVQ50;GmVQ53;GmVQ56;GmVQ57;GmVQ61 |
| GO:0051172 | negative regulation of nitrogen compound metabolic process | BP | 8 | 8 | 1 | 1 | GmVQ26;GmVQ29;GmVQ49;GmVQ50;GmVQ53;GmVQ56;GmVQ57;GmVQ61 |
| GO:0051245 | negative regulation of cellular defense response | BP | 8 | 8 | 1 | 1 | GmVQ26;GmVQ29;GmVQ49;GmVQ50;GmVQ53;GmVQ56;GmVQ57;GmVQ61 |
| GO:0051253 | negative regulation of RNA metabolic process | BP | 8 | 8 | 1 | 1 | GmVQ26;GmVQ29;GmVQ49;GmVQ50;GmVQ53;GmVQ56;GmVQ57;GmVQ61 |
| GO:1902679 | negative regulation of RNA biosynthetic process | BP | 8 | 8 | 1 | 1 | GmVQ26;GmVQ29;GmVQ49;GmVQ50;GmVQ53;GmVQ56;GmVQ57;GmVQ61 |
| GO:1903507 | negative regulation of nucleic acid-templated transcription | BP | 8 | 8 | 1 | 1 | GmVQ26;GmVQ29;GmVQ49;GmVQ50;GmVQ53;GmVQ56;GmVQ57;GmVQ61 |
| GO:2000113 | negative regulation of cellular macromolecule biosynthetic process | BP | 8 | 8 | 1 | 1 | GmVQ26;GmVQ29;GmVQ49;GmVQ50;GmVQ53;GmVQ56;GmVQ57;GmVQ61 |
| GO:0000003 | reproduction | BP | 7 | 7 | 1 | 1 | GmVQ1;GmVQ2;GmVQ28;GmVQ39;GmVQ40;GmVQ41;GmVQ45 |
| GO:0003006 | developmental process involved in reproduction | BP | 7 | 7 | 1 | 1 | GmVQ1;GmVQ2;GmVQ28;GmVQ39;GmVQ40;GmVQ41;GmVQ45 |
| GO:0022414 | reproductive process | BP | 7 | 7 | 1 | 1 | GmVQ1;GmVQ2;GmVQ28;GmVQ39;GmVQ40;GmVQ41;GmVQ45 |
| GO:0040007 | growth | BP | 7 | 7 | 1 | 1 | GmVQ1;GmVQ2;GmVQ28;GmVQ39;GmVQ40;GmVQ41;GmVQ45 |
| GO:0040008 | regulation of growth | BP | 7 | 7 | 1 | 1 | GmVQ1;GmVQ2;GmVQ28;GmVQ39;GmVQ40;GmVQ41;GmVQ45 |
| GO:0044702 | single organism reproductive process | BP | 7 | 7 | 1 | 1 | GmVQ1;GmVQ2;GmVQ28;GmVQ39;GmVQ40;GmVQ41;GmVQ45 |
| GO:0048589 | developmental growth | BP | 7 | 7 | 1 | 1 | GmVQ1;GmVQ2;GmVQ28;GmVQ39;GmVQ40;GmVQ41;GmVQ45 |
| GO:0048638 | regulation of developmental growth | BP | 7 | 7 | 1 | 1 | GmVQ1;GmVQ2;GmVQ28;GmVQ39;GmVQ40;GmVQ41;GmVQ45 |
| GO:0048856 | anatomical structure development | BP | 7 | 7 | 1 | 1 | GmVQ1;GmVQ2;GmVQ28;GmVQ39;GmVQ40;GmVQ41;GmVQ45 |
| GO:0050793 | regulation of developmental process | BP | 7 | 7 | 1 | 1 | GmVQ1;GmVQ2;GmVQ28;GmVQ39;GmVQ40;GmVQ41;GmVQ45 |
| GO:0051239 | regulation of multicellular organismal process | BP | 7 | 7 | 1 | 1 | GmVQ1;GmVQ2;GmVQ28;GmVQ39;GmVQ40;GmVQ41;GmVQ45 |
| GO:2000241 | regulation of reproductive process | BP | 7 | 7 | 1 | 1 | GmVQ1;GmVQ2;GmVQ28;GmVQ39;GmVQ40;GmVQ41;GmVQ45 |
| GO:0006793 | phosphorus metabolic process | BP | 6 | 6 | 1 | 1 | GmVQ13;GmVQ18;GmVQ19;GmVQ34;GmVQ35;GmVQ68 |
| GO:0006796 | phosphate-containing compound metabolic process | BP | 6 | 6 | 1 | 1 | GmVQ13;GmVQ18;GmVQ19;GmVQ34;GmVQ35;GmVQ68 |
| GO:0009891 | positive regulation of biosynthetic process | BP | 6 | 6 | 1 | 1 | GmVQ6;GmVQ8;GmVQ12;GmVQ54;GmVQ63;GmVQ71 |
| GO:0009893 | positive regulation of metabolic process | BP | 6 | 6 | 1 | 1 | GmVQ6;GmVQ8;GmVQ12;GmVQ54;GmVQ63;GmVQ71 |
| GO:0010557 | positive regulation of macromolecule biosynthetic process | BP | 6 | 6 | 1 | 1 | GmVQ6;GmVQ8;GmVQ12;GmVQ54;GmVQ63;GmVQ71 |
| GO:0010604 | positive regulation of macromolecule metabolic process | BP | 6 | 6 | 1 | 1 | GmVQ6;GmVQ8;GmVQ12;GmVQ54;GmVQ63;GmVQ71 |
| GO:0010628 | positive regulation of gene expression | BP | 6 | 6 | 1 | 1 | GmVQ6;GmVQ8;GmVQ12;GmVQ54;GmVQ63;GmVQ71 |
| GO:0016310 | phosphorylation | BP | 6 | 6 | 1 | 1 | GmVQ13;GmVQ18;GmVQ19;GmVQ34;GmVQ35;GmVQ68 |
| GO:0031325 | positive regulation of cellular metabolic process | BP | 6 | 6 | 1 | 1 | GmVQ6;GmVQ8;GmVQ12;GmVQ54;GmVQ63;GmVQ71 |
| GO:0031328 | positive regulation of cellular biosynthetic process | BP | 6 | 6 | 1 | 1 | GmVQ6;GmVQ8;GmVQ12;GmVQ54;GmVQ63;GmVQ71 |
| GO:0044093 | positive regulation of molecular function | BP | 6 | 6 | 1 | 1 | GmVQ6;GmVQ8;GmVQ12;GmVQ54;GmVQ63;GmVQ71 |
| GO:0045893 | positive regulation of transcription, DNA-templated | BP | 6 | 6 | 1 | 1 | GmVQ6;GmVQ8;GmVQ12;GmVQ54;GmVQ63;GmVQ71 |
| GO:0045935 | positive regulation of nucleobase-containing compound metabolic process | BP | 6 | 6 | 1 | 1 | GmVQ6;GmVQ8;GmVQ12;GmVQ54;GmVQ63;GmVQ71 |
| GO:0048522 | positive regulation of cellular process | BP | 6 | 6 | 1 | 1 | GmVQ6;GmVQ8;GmVQ12;GmVQ54;GmVQ63;GmVQ71 |
| GO:0051091 | positive regulation of sequence-specific DNA binding transcription factor activity | BP | 6 | 6 | 1 | 1 | GmVQ6;GmVQ8;GmVQ12;GmVQ54;GmVQ63;GmVQ71 |
| GO:0051173 | positive regulation of nitrogen compound metabolic process | BP | 6 | 6 | 1 | 1 | GmVQ6;GmVQ8;GmVQ12;GmVQ54;GmVQ63;GmVQ71 |
| GO:0051254 | positive regulation of RNA metabolic process | BP | 6 | 6 | 1 | 1 | GmVQ6;GmVQ8;GmVQ12;GmVQ54;GmVQ63;GmVQ71 |
| GO:1902680 | positive regulation of RNA biosynthetic process | BP | 6 | 6 | 1 | 1 | GmVQ6;GmVQ8;GmVQ12;GmVQ54;GmVQ63;GmVQ71 |
| GO:1903508 | positive regulation of nucleic acid-templated transcription | BP | 6 | 6 | 1 | 1 | GmVQ6;GmVQ8;GmVQ12;GmVQ54;GmVQ63;GmVQ71 |
| GO:0009888 | tissue development | BP | 5 | 5 | 1 | 1 | GmVQ1;GmVQ2;GmVQ39;GmVQ40;GmVQ41 |
| GO:0009960 | endosperm development | BP | 5 | 5 | 1 | 1 | GmVQ1;GmVQ2;GmVQ39;GmVQ40;GmVQ41 |
| GO:0010154 | fruit development | BP | 5 | 5 | 1 | 1 | GmVQ1;GmVQ2;GmVQ39;GmVQ40;GmVQ41 |
| GO:0048316 | seed development | BP | 5 | 5 | 1 | 1 | GmVQ1;GmVQ2;GmVQ39;GmVQ40;GmVQ41 |
| GO:0048580 | regulation of post-embryonic development | BP | 5 | 5 | 1 | 1 | GmVQ1;GmVQ2;GmVQ39;GmVQ40;GmVQ41 |
| GO:0048608 | reproductive structure development | BP | 5 | 5 | 1 | 1 | GmVQ1;GmVQ2;GmVQ39;GmVQ40;GmVQ41 |
| GO:0048731 | system development | BP | 5 | 5 | 1 | 1 | GmVQ1;GmVQ2;GmVQ39;GmVQ40;GmVQ41 |
| GO:0061458 | reproductive system development | BP | 5 | 5 | 1 | 1 | GmVQ1;GmVQ2;GmVQ39;GmVQ40;GmVQ41 |
| GO:0080050 | regulation of seed development | BP | 5 | 5 | 1 | 1 | GmVQ1;GmVQ2;GmVQ39;GmVQ40;GmVQ41 |
| GO:0080112 | seed growth | BP | 5 | 5 | 1 | 1 | GmVQ1;GmVQ2;GmVQ39;GmVQ40;GmVQ41 |
| GO:0080113 | regulation of seed growth | BP | 5 | 5 | 1 | 1 | GmVQ1;GmVQ2;GmVQ39;GmVQ40;GmVQ41 |
| GO:2000026 | regulation of multicellular organismal development | BP | 5 | 5 | 1 | 1 | GmVQ1;GmVQ2;GmVQ39;GmVQ40;GmVQ41 |
| GO:0006970 | response to osmotic stress | BP | 3 | 3 | 1 | 1 | GmVQ4;GmVQ9;GmVQ21 |
| GO:0009628 | response to abiotic stimulus | BP | 3 | 3 | 1 | 1 | GmVQ4;GmVQ9;GmVQ21 |
| GO:0009651 | response to salt stress | BP | 3 | 3 | 1 | 1 | GmVQ4;GmVQ9;GmVQ21 |
| GO:0010033 | response to organic substance | BP | 3 | 3 | 1 | 1 | GmVQ30;GmVQ36;GmVQ65 |
| GO:0042221 | response to chemical | BP | 3 | 3 | 1 | 1 | GmVQ30;GmVQ36;GmVQ65 |
| GO:0043900 | regulation of multi-organism process | BP | 3 | 3 | 1 | 1 | GmVQ28;GmVQ30;GmVQ45 |
| GO:0047484 | regulation of response to osmotic stress | BP | 3 | 3 | 1 | 1 | GmVQ4;GmVQ9;GmVQ21 |
| GO:0051704 | multi-organism process | BP | 3 | 3 | 1 | 1 | GmVQ28;GmVQ30;GmVQ45 |
| GO:0070887 | cellular response to chemical stimulus | BP | 3 | 3 | 1 | 1 | GmVQ30;GmVQ36;GmVQ65 |
| GO:0071310 | cellular response to organic substance | BP | 3 | 3 | 1 | 1 | GmVQ30;GmVQ36;GmVQ65 |
| GO:1901000 | regulation of response to salt stress | BP | 3 | 3 | 1 | 1 | GmVQ4;GmVQ9;GmVQ21 |
| GO:1901001 | negative regulation of response to salt stress | BP | 3 | 3 | 1 | 1 | GmVQ4;GmVQ9;GmVQ21 |
| GO:0000902 | cell morphogenesis | BP | 2 | 2 | 1 | 1 | GmVQ28;GmVQ45 |
| GO:0000904 | cell morphogenesis involved in differentiation | BP | 2 | 2 | 1 | 1 | GmVQ28;GmVQ45 |
| GO:0001101 | response to acid chemical | BP | 2 | 2 | 1 | 1 | GmVQ36;GmVQ65 |
| GO:0001558 | regulation of cell growth | BP | 2 | 2 | 1 | 1 | GmVQ28;GmVQ45 |
| GO:0009653 | anatomical structure morphogenesis | BP | 2 | 2 | 1 | 1 | GmVQ28;GmVQ45 |
| GO:0009719 | response to endogenous stimulus | BP | 2 | 2 | 1 | 1 | GmVQ36;GmVQ65 |
| GO:0009725 | response to hormone | BP | 2 | 2 | 1 | 1 | GmVQ36;GmVQ65 |
| GO:0009737 | response to abscisic acid | BP | 2 | 2 | 1 | 1 | GmVQ36;GmVQ65 |
| GO:0009826 | unidimensional cell growth | BP | 2 | 2 | 1 | 1 | GmVQ28;GmVQ45 |
| GO:0009856 | pollination | BP | 2 | 2 | 1 | 1 | GmVQ28;GmVQ45 |
| GO:0009860 | pollen tube growth | BP | 2 | 2 | 1 | 1 | GmVQ28;GmVQ45 |
| GO:0009932 | cell tip growth | BP | 2 | 2 | 1 | 1 | GmVQ28;GmVQ45 |
| GO:0010769 | regulation of cell morphogenesis involved in differentiation | BP | 2 | 2 | 1 | 1 | GmVQ28;GmVQ45 |
| GO:0016043 | cellular component organization | BP | 2 | 2 | 1 | 1 | GmVQ28;GmVQ45 |
| GO:0016049 | cell growth | BP | 2 | 2 | 1 | 1 | GmVQ28;GmVQ45 |
| GO:0022603 | regulation of anatomical structure morphogenesis | BP | 2 | 2 | 1 | 1 | GmVQ28;GmVQ45 |
| GO:0022604 | regulation of cell morphogenesis | BP | 2 | 2 | 1 | 1 | GmVQ28;GmVQ45 |
| GO:0030154 | cell differentiation | BP | 2 | 2 | 1 | 1 | GmVQ28;GmVQ45 |
| GO:0032870 | cellular response to hormone stimulus | BP | 2 | 2 | 1 | 1 | GmVQ36;GmVQ65 |
| GO:0032989 | cellular component morphogenesis | BP | 2 | 2 | 1 | 1 | GmVQ28;GmVQ45 |
| GO:0033993 | response to lipid | BP | 2 | 2 | 1 | 1 | GmVQ36;GmVQ65 |
| GO:0044703 | multi-organism reproductive process | BP | 2 | 2 | 1 | 1 | GmVQ28;GmVQ45 |
| GO:0044706 | multi-multicellular organism process | BP | 2 | 2 | 1 | 1 | GmVQ28;GmVQ45 |
| GO:0045595 | regulation of cell differentiation | BP | 2 | 2 | 1 | 1 | GmVQ28;GmVQ45 |
| GO:0048468 | cell development | BP | 2 | 2 | 1 | 1 | GmVQ28;GmVQ45 |
| GO:0048588 | developmental cell growth | BP | 2 | 2 | 1 | 1 | GmVQ28;GmVQ45 |
| GO:0048868 | pollen tube development | BP | 2 | 2 | 1 | 1 | GmVQ28;GmVQ45 |
| GO:0048869 | cellular developmental process | BP | 2 | 2 | 1 | 1 | GmVQ28;GmVQ45 |
| GO:0051128 | regulation of cellular component organization | BP | 2 | 2 | 1 | 1 | GmVQ28;GmVQ45 |
| GO:0051510 | regulation of unidimensional cell growth | BP | 2 | 2 | 1 | 1 | GmVQ28;GmVQ45 |
| GO:0060284 | regulation of cell development | BP | 2 | 2 | 1 | 1 | GmVQ28;GmVQ45 |
| GO:0060560 | developmental growth involved in morphogenesis | BP | 2 | 2 | 1 | 1 | GmVQ28;GmVQ45 |
| GO:0071215 | cellular response to abscisic acid stimulus | BP | 2 | 2 | 1 | 1 | GmVQ36;GmVQ65 |
| GO:0071229 | cellular response to acid chemical | BP | 2 | 2 | 1 | 1 | GmVQ36;GmVQ65 |
| GO:0071396 | cellular response to lipid | BP | 2 | 2 | 1 | 1 | GmVQ36;GmVQ65 |
| GO:0071495 | cellular response to endogenous stimulus | BP | 2 | 2 | 1 | 1 | GmVQ36;GmVQ65 |
| GO:0071840 | cellular component organization or biogenesis | BP | 2 | 2 | 1 | 1 | GmVQ28;GmVQ45 |
| GO:0080092 | regulation of pollen tube growth | BP | 2 | 2 | 1 | 1 | GmVQ28;GmVQ45 |
| GO:0090351 | seedling development | BP | 2 | 2 | 1 | 1 | GmVQ36;GmVQ65 |
| GO:0097305 | response to alcohol | BP | 2 | 2 | 1 | 1 | GmVQ36;GmVQ65 |
| GO:0097306 | cellular response to alcohol | BP | 2 | 2 | 1 | 1 | GmVQ36;GmVQ65 |
| GO:1901700 | response to oxygen-containing compound | BP | 2 | 2 | 1 | 1 | GmVQ36;GmVQ65 |
| GO:1901701 | cellular response to oxygen-containing compound | BP | 2 | 2 | 1 | 1 | GmVQ36;GmVQ65 |
| GO:0002252 | immune effector process | BP | 1 | 1 | 1 | 1 | GmVQ30 |
| GO:0002697 | regulation of immune effector process | BP | 1 | 1 | 1 | 1 | GmVQ30 |
| GO:0002831 | regulation of response to biotic stimulus | BP | 1 | 1 | 1 | 1 | GmVQ30 |
| GO:0006396 | RNA processing | BP | 1 | 1 | 1 | 1 | GmVQ30 |
| GO:0009605 | response to external stimulus | BP | 1 | 1 | 1 | 1 | GmVQ30 |
| GO:0009607 | response to biotic stimulus | BP | 1 | 1 | 1 | 1 | GmVQ30 |
| GO:0009615 | response to virus | BP | 1 | 1 | 1 | 1 | GmVQ30 |
| GO:0009616 | virus induced gene silencing | BP | 1 | 1 | 1 | 1 | GmVQ30 |
| GO:0010025 | wax biosynthetic process | BP | 1 | 1 | 1 | 1 | GmVQ30 |
| GO:0010050 | vegetative phase change | BP | 1 | 1 | 1 | 1 | GmVQ30 |
| GO:0010166 | wax metabolic process | BP | 1 | 1 | 1 | 1 | GmVQ30 |
| GO:0010267 | production of ta-siRNAs involved in RNA interference | BP | 1 | 1 | 1 | 1 | GmVQ30 |
| GO:0010608 | posttranscriptional regulation of gene expression | BP | 1 | 1 | 1 | 1 | GmVQ30 |
| GO:0014070 | response to organic cyclic compound | BP | 1 | 1 | 1 | 1 | GmVQ30 |
| GO:0016032 | viral process | BP | 1 | 1 | 1 | 1 | GmVQ30 |
| GO:0016246 | RNA interference | BP | 1 | 1 | 1 | 1 | GmVQ30 |
| GO:0016441 | posttranscriptional gene silencing | BP | 1 | 1 | 1 | 1 | GmVQ30 |
| GO:0016458 | gene silencing | BP | 1 | 1 | 1 | 1 | GmVQ30 |
| GO:0019722 | calcium-mediated signaling | BP | 1 | 1 | 1 | 1 | GmVQ56 |
| GO:0019932 | second-messenger-mediated signaling | BP | 1 | 1 | 1 | 1 | GmVQ56 |
| GO:0030422 | production of siRNA involved in RNA interference | BP | 1 | 1 | 1 | 1 | GmVQ30 |
| GO:0031047 | gene silencing by RNA | BP | 1 | 1 | 1 | 1 | GmVQ30 |
| GO:0031050 | dsRNA fragmentation | BP | 1 | 1 | 1 | 1 | GmVQ30 |
| GO:0032101 | regulation of response to external stimulus | BP | 1 | 1 | 1 | 1 | GmVQ30 |
| GO:0035194 | posttranscriptional gene silencing by RNA | BP | 1 | 1 | 1 | 1 | GmVQ30 |
| GO:0035821 | modification of morphology or physiology of other organism | BP | 1 | 1 | 1 | 1 | GmVQ30 |
| GO:0040029 | regulation of gene expression, epigenetic | BP | 1 | 1 | 1 | 1 | GmVQ30 |
| GO:0043207 | response to external biotic stimulus | BP | 1 | 1 | 1 | 1 | GmVQ30 |
| GO:0043331 | response to dsRNA | BP | 1 | 1 | 1 | 1 | GmVQ30 |
| GO:0044003 | modification by symbiont of host morphology or physiology | BP | 1 | 1 | 1 | 1 | GmVQ30 |
| GO:0044403 | symbiosis, encompassing mutualism through parasitism | BP | 1 | 1 | 1 | 1 | GmVQ30 |
| GO:0044419 | interspecies interaction between organisms | BP | 1 | 1 | 1 | 1 | GmVQ30 |
| GO:0044710 | single-organism metabolic process | BP | 1 | 1 | 1 | 1 | GmVQ30 |
| GO:0044764 | multi-organism cellular process | BP | 1 | 1 | 1 | 1 | GmVQ30 |
| GO:0050688 | regulation of defense response to virus | BP | 1 | 1 | 1 | 1 | GmVQ30 |
| GO:0051607 | defense response to virus | BP | 1 | 1 | 1 | 1 | GmVQ30 |
| GO:0051701 | interaction with host | BP | 1 | 1 | 1 | 1 | GmVQ30 |
| GO:0051707 | response to other organism | BP | 1 | 1 | 1 | 1 | GmVQ30 |
| GO:0051817 | modification of morphology or physiology of other organism involved in symbiotic interaction | BP | 1 | 1 | 1 | 1 | GmVQ30 |
| GO:0052018 | modulation by symbiont of RNA levels in host | BP | 1 | 1 | 1 | 1 | GmVQ30 |
| GO:0052249 | modulation of RNA levels in other organism involved in symbiotic interaction | BP | 1 | 1 | 1 | 1 | GmVQ30 |
| GO:0065008 | regulation of biological quality | BP | 1 | 1 | 1 | 1 | GmVQ30 |
| GO:0070918 | production of small RNA involved in gene silencing by RNA | BP | 1 | 1 | 1 | 1 | GmVQ30 |
| GO:0071359 | cellular response to dsRNA | BP | 1 | 1 | 1 | 1 | GmVQ30 |
| GO:0071407 | cellular response to organic cyclic compound | BP | 1 | 1 | 1 | 1 | GmVQ30 |
| GO:0098542 | defense response to other organism | BP | 1 | 1 | 1 | 1 | GmVQ30 |
| GO:0098586 | cellular response to virus | BP | 1 | 1 | 1 | 1 | GmVQ30 |
| GO:1901698 | response to nitrogen compound | BP | 1 | 1 | 1 | 1 | GmVQ30 |
| GO:1901699 | cellular response to nitrogen compound | BP | 1 | 1 | 1 | 1 | GmVQ30 |
| GO:0003674 | molecular_function | MF | 18 | 18 | 1 | 1 | GmVQ5;GmVQ13;GmVQ18;GmVQ19;GmVQ26;GmVQ29;GmVQ30;GmVQ34;GmVQ35;GmVQ48;GmVQ49;GmVQ50;GmVQ53;GmVQ56;GmVQ57;GmVQ61;GmVQ68;GmVQ70 |
| GO:0005488 | binding | MF | 15 | 15 | 1 | 1 | GmVQ5;GmVQ18;GmVQ26;GmVQ29;GmVQ30;GmVQ34;GmVQ48;GmVQ49;GmVQ50;GmVQ53;GmVQ56;GmVQ57;GmVQ61;GmVQ68;GmVQ70 |
| GO:0003676 | nucleic acid binding | MF | 12 | 12 | 1 | 1 | GmVQ18;GmVQ26;GmVQ29;GmVQ34;GmVQ48;GmVQ49;GmVQ50;GmVQ53;GmVQ56;GmVQ57;GmVQ61;GmVQ68 |
| GO:0003677 | DNA binding | MF | 12 | 12 | 1 | 1 | GmVQ18;GmVQ26;GmVQ29;GmVQ34;GmVQ48;GmVQ49;GmVQ50;GmVQ53;GmVQ56;GmVQ57;GmVQ61;GmVQ68 |
| GO:0097159 | organic cyclic compound binding | MF | 12 | 12 | 1 | 1 | GmVQ18;GmVQ26;GmVQ29;GmVQ34;GmVQ48;GmVQ49;GmVQ50;GmVQ53;GmVQ56;GmVQ57;GmVQ61;GmVQ68 |
| GO:1901363 | heterocyclic compound binding | MF | 12 | 12 | 1 | 1 | GmVQ18;GmVQ26;GmVQ29;GmVQ34;GmVQ48;GmVQ49;GmVQ50;GmVQ53;GmVQ56;GmVQ57;GmVQ61;GmVQ68 |
| GO:0005515 | protein binding | MF | 11 | 11 | 1 | 1 | GmVQ5;GmVQ26;GmVQ29;GmVQ30;GmVQ49;GmVQ50;GmVQ53;GmVQ56;GmVQ57;GmVQ61;GmVQ70 |
| GO:0001071 | nucleic acid binding transcription factor activity | MF | 8 | 8 | 1 | 1 | GmVQ26;GmVQ29;GmVQ49;GmVQ50;GmVQ53;GmVQ56;GmVQ57;GmVQ61 |
| GO:0003700 | transcription factor activity, sequence-specific DNA binding | MF | 8 | 8 | 1 | 1 | GmVQ26;GmVQ29;GmVQ49;GmVQ50;GmVQ53;GmVQ56;GmVQ57;GmVQ61 |
| GO:0043565 | sequence-specific DNA binding | MF | 8 | 8 | 1 | 1 | GmVQ26;GmVQ29;GmVQ49;GmVQ50;GmVQ53;GmVQ56;GmVQ57;GmVQ61 |
| GO:0003824 | catalytic activity | MF | 6 | 6 | 1 | 1 | GmVQ13;GmVQ18;GmVQ19;GmVQ34;GmVQ35;GmVQ68 |
| GO:0016301 | kinase activity | MF | 6 | 6 | 1 | 1 | GmVQ13;GmVQ18;GmVQ19;GmVQ34;GmVQ35;GmVQ68 |
| GO:0016740 | transferase activity | MF | 6 | 6 | 1 | 1 | GmVQ13;GmVQ18;GmVQ19;GmVQ34;GmVQ35;GmVQ68 |
| GO:0016772 | transferase activity, transferring phosphorus-containing groups | MF | 6 | 6 | 1 | 1 | GmVQ13;GmVQ18;GmVQ19;GmVQ34;GmVQ35;GmVQ68 |
| GO:0005516 | calmodulin binding | MF | 2 | 2 | 1 | 1 | GmVQ5;GmVQ70 |
| GO:0005575 | cellular_component | CC | 27 | 27 | 1 | 1 | GmVQ5;GmVQ7;GmVQ13;GmVQ14;GmVQ19;GmVQ24;GmVQ25;GmVQ26;GmVQ28;GmVQ29;GmVQ30;GmVQ31;GmVQ35;GmVQ36;GmVQ44;GmVQ45;GmVQ46;GmVQ49;GmVQ50;GmVQ52;GmVQ53;GmVQ56;GmVQ57;GmVQ59;GmVQ61;GmVQ65;GmVQ70 |
| GO:0005622 | intracellular | CC | 27 | 27 | 1 | 1 | GmVQ5;GmVQ7;GmVQ13;GmVQ14;GmVQ19;GmVQ24;GmVQ25;GmVQ26;GmVQ28;GmVQ29;GmVQ30;GmVQ31;GmVQ35;GmVQ36;GmVQ44;GmVQ45;GmVQ46;GmVQ49;GmVQ50;GmVQ52;GmVQ53;GmVQ56;GmVQ57;GmVQ59;GmVQ61;GmVQ65;GmVQ70 |
| GO:0005623 | cell | CC | 27 | 27 | 1 | 1 | GmVQ5;GmVQ7;GmVQ13;GmVQ14;GmVQ19;GmVQ24;GmVQ25;GmVQ26;GmVQ28;GmVQ29;GmVQ30;GmVQ31;GmVQ35;GmVQ36;GmVQ44;GmVQ45;GmVQ46;GmVQ49;GmVQ50;GmVQ52;GmVQ53;GmVQ56;GmVQ57;GmVQ59;GmVQ61;GmVQ65;GmVQ70 |
| GO:0043226 | organelle | CC | 27 | 27 | 1 | 1 | GmVQ5;GmVQ7;GmVQ13;GmVQ14;GmVQ19;GmVQ24;GmVQ25;GmVQ26;GmVQ28;GmVQ29;GmVQ30;GmVQ31;GmVQ35;GmVQ36;GmVQ44;GmVQ45;GmVQ46;GmVQ49;GmVQ50;GmVQ52;GmVQ53;GmVQ56;GmVQ57;GmVQ59;GmVQ61;GmVQ65;GmVQ70 |
| GO:0043227 | membrane-bounded organelle | CC | 27 | 27 | 1 | 1 | GmVQ5;GmVQ7;GmVQ13;GmVQ14;GmVQ19;GmVQ24;GmVQ25;GmVQ26;GmVQ28;GmVQ29;GmVQ30;GmVQ31;GmVQ35;GmVQ36;GmVQ44;GmVQ45;GmVQ46;GmVQ49;GmVQ50;GmVQ52;GmVQ53;GmVQ56;GmVQ57;GmVQ59;GmVQ61;GmVQ65;GmVQ70 |
| GO:0043229 | intracellular organelle | CC | 27 | 27 | 1 | 1 | GmVQ5;GmVQ7;GmVQ13;GmVQ14;GmVQ19;GmVQ24;GmVQ25;GmVQ26;GmVQ28;GmVQ29;GmVQ30;GmVQ31;GmVQ35;GmVQ36;GmVQ44;GmVQ45;GmVQ46;GmVQ49;GmVQ50;GmVQ52;GmVQ53;GmVQ56;GmVQ57;GmVQ59;GmVQ61;GmVQ65;GmVQ70 |
| GO:0043231 | intracellular membrane-bounded organelle | CC | 27 | 27 | 1 | 1 | GmVQ5;GmVQ7;GmVQ13;GmVQ14;GmVQ19;GmVQ24;GmVQ25;GmVQ26;GmVQ28;GmVQ29;GmVQ30;GmVQ31;GmVQ35;GmVQ36;GmVQ44;GmVQ45;GmVQ46;GmVQ49;GmVQ50;GmVQ52;GmVQ53;GmVQ56;GmVQ57;GmVQ59;GmVQ61;GmVQ65;GmVQ70 |
| GO:0044424 | intracellular part | CC | 27 | 27 | 1 | 1 | GmVQ5;GmVQ7;GmVQ13;GmVQ14;GmVQ19;GmVQ24;GmVQ25;GmVQ26;GmVQ28;GmVQ29;GmVQ30;GmVQ31;GmVQ35;GmVQ36;GmVQ44;GmVQ45;GmVQ46;GmVQ49;GmVQ50;GmVQ52;GmVQ53;GmVQ56;GmVQ57;GmVQ59;GmVQ61;GmVQ65;GmVQ70 |
| GO:0044464 | cell part | CC | 27 | 27 | 1 | 1 | GmVQ5;GmVQ7;GmVQ13;GmVQ14;GmVQ19;GmVQ24;GmVQ25;GmVQ26;GmVQ28;GmVQ29;GmVQ30;GmVQ31;GmVQ35;GmVQ36;GmVQ44;GmVQ45;GmVQ46;GmVQ49;GmVQ50;GmVQ52;GmVQ53;GmVQ56;GmVQ57;GmVQ59;GmVQ61;GmVQ65;GmVQ70 |
| GO:0005634 | nucleus | CC | 26 | 26 | 1 | 1 | GmVQ5;GmVQ7;GmVQ13;GmVQ14;GmVQ19;GmVQ24;GmVQ25;GmVQ26;GmVQ28;GmVQ29;GmVQ31;GmVQ35;GmVQ36;GmVQ44;GmVQ45;GmVQ46;GmVQ49;GmVQ50;GmVQ52;GmVQ53;GmVQ56;GmVQ57;GmVQ59;GmVQ61;GmVQ65;GmVQ70 |
| GO:0016020 | membrane | CC | 8 | 8 | 1 | 1 | GmVQ26;GmVQ29;GmVQ49;GmVQ50;GmVQ53;GmVQ56;GmVQ57;GmVQ61 |
| GO:0016021 | integral component of membrane | CC | 8 | 8 | 1 | 1 | GmVQ26;GmVQ29;GmVQ49;GmVQ50;GmVQ53;GmVQ56;GmVQ57;GmVQ61 |
| GO:0031224 | intrinsic component of membrane | CC | 8 | 8 | 1 | 1 | GmVQ26;GmVQ29;GmVQ49;GmVQ50;GmVQ53;GmVQ56;GmVQ57;GmVQ61 |
| GO:0044425 | membrane part | CC | 8 | 8 | 1 | 1 | GmVQ26;GmVQ29;GmVQ49;GmVQ50;GmVQ53;GmVQ56;GmVQ57;GmVQ61 |
| GO:0005737 | cytoplasm | CC | 1 | 1 | 1 | 1 | GmVQ30 |
| GO:0005783 | endoplasmic reticulum | CC | 1 | 1 | 1 | 1 | GmVQ30 |
| GO:0005829 | cytosol | CC | 1 | 1 | 1 | 1 | GmVQ30 |
| GO:0005911 | cell-cell junction | CC | 1 | 1 | 1 | 1 | GmVQ56 |
| GO:0009506 | plasmodesma | CC | 1 | 1 | 1 | 1 | GmVQ56 |
| GO:0012505 | endomembrane system | CC | 1 | 1 | 1 | 1 | GmVQ30 |
| GO:0030054 | cell junction | CC | 1 | 1 | 1 | 1 | GmVQ56 |
| GO:0044444 | cytoplasmic part | CC | 1 | 1 | 1 | 1 | GmVQ30 |
| GO:0048471 | perinuclear region of cytoplasm | CC | 1 | 1 | 1 | 1 | GmVQ30 |
| GO:0055044 | symplast | CC | 1 | 1 | 1 | 1 | GmVQ56 |
